# Non-traditional metabolic indices predict incident circadian syndrome in middle-aged and older Chinese adults: a nationwide prospective cohort study and machine learning analysis

**DOI:** 10.1186/s12944-026-02972-9

**Published:** 2026-05-13

**Authors:** Kangrong Li, Gaoming Zeng, Siyuan Tan, Zixi Zhang, Jiayi Zhu, Zhongjun Ma, Qiuzhen Lin, Zhenjiang Liu, Na Liu, Qiming Liu

**Affiliations:** Department of Cardiovascular Medicine, The Second Xiangya Hospital of Central South University, 139 Renmin Road, Changsha, Hunan 410011 China

**Keywords:** Circadian syndrome, Insulin resistance, Atherogenic index of plasma, Estimated glucose disposal rate, Machine learning, CHARLS

## Abstract

**Background:**

Circadian syndrome (CircS) augments the conventional metabolic syndrome construct by adding disturbed sleep and depressive features. Whether composite metabolic indices that combine insulin-resistance, atherogenic-lipid, and inflammatory signals can forecast CircS prior to its onset has not been systematically investigated. The present work evaluated eight such composite markers in a population-based sample of Chinese adults aged 45 years or older.

**Methods:**

Drawing on the China Health and Retirement Longitudinal Study (CHARLS), we followed 4,325 CircS-free adults from 2011 through 2015. Eight baseline metabolic composites were analysed through robust-variance modified Poisson regression, four-knot restricted cubic splines, incremental receiver operating characteristic (ROC) metrics, bidirectional mediation under a quasi-Bayesian framework, multiple sensitivity checks, and a head-to-head benchmarking of 10 machine-learning algorithms complemented by SHapley Additive exPlanations (SHAP) interpretation.

**Results:**

Over 4 years, 1,025 incident CircS cases (23.7%) accrued. Every index remained independently linked to CircS once multivariable adjustment was applied. The steepest positive gradient belonged to the triglyceride-glucose body mass index (TyG-BMI; extreme-quartile risk ratio [RR] 4.56, 95% CI 3.35—6.21; per-standard-deviation RR 1.87, 95% CI 1.66—2.10), whilst the estimated glucose disposal rate (eGDR) demonstrated the most pronounced inverse gradient (RR 0.28, 95% CI 0.20—0.38). The largest discrimination gain belonged to the cholesterol–HDL-C–glucose (CHG) index (area under the curve [AUC] 0.737; continuous net reclassification improvement 0.379; DeLong *P* < 0.001). Reverse-path mediation indicated that the CHG index and the metabolic score for insulin resistance (METS-IR) jointly carried part of the high-sensitivity C-reactive protein (hs-CRP)–CircS signal. On the held-out test set, logistic regression reached the top area under the curve (0.746), and the XGBoost SHAP ranking placed eGDR first among predictors.

**Conclusions:**

Eight non-traditional metabolic composites anticipated incident CircS, and within this panel eGDR, TyG-BMI, and the CHG index carried the most consistent predictive information. Incorporating such readily obtainable indices into routine assessment could facilitate earlier CircS risk identification in ageing populations.

**Supplementary Information:**

The online version contains supplementary material available at 10.1186/s12944-026-02972-9.

## Background

As global populations age, rising metabolic morbidity has prompted renewed efforts to stratify cardiometabolic risk beyond traditional criteria. For decades, metabolic syndrome (MetS)—a co-occurrence of abdominal obesity, atherogenic dyslipidaemia, raised fasting glucose, and elevated blood pressure—has served as the dominant organising construct for such risk assessment [1, 2]. Roughly one in three adults worldwide fulfils MetS criteria, and the phenotype reliably forecasts cardiovascular events, new-onset type 2 diabetes, and premature mortality [3, 4]. Yet the MetS framework says little about disturbances of the biological clock. Partly in response, Zimmet and colleagues introduced circadian syndrome (CircS), which layers abnormal sleep duration and depressive symptoms onto the traditional MetS components [5, 6]. The enlarged construct acknowledges the two-way traffic between clock-gene disruption and metabolic dysfunction—a relationship of expanding public-health salience amid increasing night work, irregular sleep schedules, and widespread evening exposure to artificial light [7, 8].

Observational studies have tied CircS to cardiovascular disease [9], unfavourable glycaemic outcomes [10], cognitive decline [11], and all-cause mortality [12]. Diagnostically, CircS is anchored on any four of seven features—central adiposity assessed by waist circumference (WC), hypertension, low high-density lipoprotein cholesterol (HDL-C), hypertriglyceridaemia, hyperglycaemia, abnormal sleep duration, and clinically relevant depressive symptomatology [5]. Despite the clinical salience of the construct, candidate biomarkers that might anticipate its onset have attracted only limited attention, and the bulk of the published literature positions CircS as a predictor of downstream morbidity rather than as an endpoint in its own right [13, 14]. As a result, upstream screening tools capable of flagging individuals at elevated CircS risk remain a clear unmet need.

Recognition of insulin resistance as a systemic driver of metabolic disease has catalysed a move from single-marker readouts toward composite metabolic scores [15]—part of a broader reorientation from conventional risk factors toward multi-pathway cardiometabolic phenotyping [16]. Composite indices that bring together signals from several pathophysiological axes are increasingly viewed as promising tools for cardiometabolic risk assessment, and four mechanistic domains can be distinguished within the current panel: insulin-resistance surrogates such as the triglyceride-glucose body mass index (TyG-BMI) [17] and the metabolic score for insulin resistance (METS-IR) [18]; an insulin-sensitivity proxy, the estimated glucose disposal rate (eGDR), developed initially for type 1 diabetes but later applied in non-diabetic populations as a marker of residual insulin sensitivity [19]; atherogenic-lipid scores, namely the atherogenic index of plasma (AIP) [20] and the cholesterol–HDL-C–glucose (CHG) index [21]; and lipid-inflammatory composites, comprising the remnant cholesterol inflammatory index (RCII) [22] and the C-reactive protein–triglyceride–glucose index (CTI) [23]. Each index has been linked to cardiovascular disease [24, 25], stroke [26], or cardiometabolic multimorbidity [27]. Whether they can prospectively anticipate incident CircS—a phenotype that spans metabolic dysregulation and circadian disturbance—is, however, unresolved.

Filling this evidence gap is the aim of the present analysis, in which we draw on the China Health and Retirement Longitudinal Study (CHARLS) to quantify the prospective relationship between eight composite metabolic indices—spanning the four domains noted above—and incident CircS. Analytical components comprise robust-variance modified Poisson regression, four-knot restricted cubic splines, incremental predictive-value metrics, bidirectional mediation anchored on biological-age acceleration, and ten machine-learning algorithms accompanied by SHapley Additive exPlanations (SHAP) interpretation [28, 29]. The design was chosen to capture both magnitude of association and clinical usefulness in a single integrated framework.

## Methods

### Study design and population

Source data were obtained from CHARLS, a population-representative panel study of Chinese residents aged 45 years and above [30]. The baseline wave began in 2011 and recruited 17,708 individuals distributed across 28 provinces and 450 primary sampling units. Venous blood specimens were collected during Wave 1 (2011–2012) and again during Wave 3 (2015). Ethical clearance for CHARLS was granted by the Biomedical Ethics Committee of Peking University (approval IRB00001052—11,015), and every respondent signed written informed consent. De-identified data are openly accessible via the CHARLS repository at http://charls.pku.edu.cn/.

**Fig. 1:**
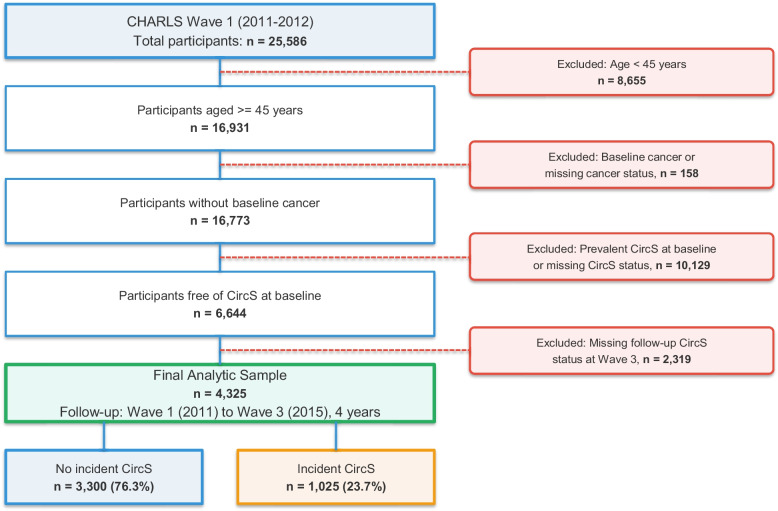
Flow diagram of participant selection. Starting with 25,586 CHARLS respondents, progressive filters were applied in this order: younger than 45 years (*n* = 8,655 removed), history of cancer or unknown cancer status (*n* = 158), prevalent circadian syndrome or undetermined circadian syndrome status at the 2011 baseline (*n* = 10,129), and undetermined circadian syndrome status at the 2015 follow-up (*n* = 2,319). The retained analytic sample was 4,325 individuals, of whom 1,025 (23.7%) experienced a first episode of circadian syndrome over the 4-year observation period. Abbreviations: CircS, circadian syndrome; CHARLS, China Health and Retirement Longitudinal Study

Beginning with 25,586 CHARLS registrants, respondents were progressively removed according to the following criteria (summarised in Fig. 1 and Table S1): younger than 45 years (*n* = 8,655), history of cancer or unknown cancer status (*n* = 158), prevalent CircS or undetermined CircS at baseline (*n* = 10,129), and undetermined CircS at Wave 3 (*n* = 2,319). The retained analytical sample numbered 4,325 participants. Table S2 summarises variable-specific missingness across the full CHARLS cohort. The high proportion of laboratory missingness (approximately 54%) mirrored the sub-study design in which only a pre-planned subset of Wave 1 respondents provided venous biomarkers, rather than dropout driven by health status. We therefore adopted a missing-at-random (MAR) assumption conditional on observed demographics and clinical variables; non-laboratory fields (sociodemographics, self-reported illness) carried far lower missingness (0–37%). Primary estimates were derived from complete-case analysis, and reporting follows the Strengthening the Reporting of Observational Studies in Epidemiology (STROBE) reporting guideline [31].

### Assessment of metabolic indices

All eight composite metabolic indices were derived from Wave 1 data and classified into four pathophysiological categories. Atherogenic-lipid scores comprised AIP, defined as log10[triglycerides (TG)/HDL-C] [20], and the CHG index, calculated as total cholesterol (TC) × fasting plasma glucose (FPG)/HDL-C [21]. Lipid-inflammatory composites comprised RCII, computed as remnant cholesterol (RC) × high-sensitivity C-reactive protein (hs-CRP), with RC equal to TC − HDL-C − low-density lipoprotein cholesterol (LDL-C) [22], together with the hs-CRP/HDL-C ratio. The insulin-resistance-plus-inflammation composite CTI was calculated as TG × FPG × hs-CRP/HDL-C [23]. The insulin-resistance-plus-adiposity composite TyG-BMI was computed as ln[TG × FPG/2] × body mass index (BMI) [17]. Insulin sensitivity was captured through eGDR, using the published Eq. 21.158 − 0.09 × WC − 3.407 × hypertension (HTN) − 0.551 × glycated haemoglobin (HbA1c) [19]. Global insulin resistance was summarised by METS-IR, defined as ln[(2 × FPG + TG) × BMI]/ln[HDL-C] [18]. Extreme values in every index were trimmed by Winsorisation at the 1 st and 99th percentiles.

### Assessment of circadian syndrome

Following Zimmet et al. [5], CircS was operationalised by the concurrent presence of any four out of seven features. (i) Central adiposity indexed by a waist circumference of ≥ 85 cm regardless of sex, a unified threshold retained for consistency with the original CircS framework [5] and earlier CHARLS-based CircS analyses; the Chinese clinical cutoffs of ≥ 90 cm for men and ≥ 85 cm for women were therefore not applied in the primary definition. (ii) Raised blood pressure, defined as systolic ≥ 130 mmHg, diastolic ≥ 85 mmHg, a physician diagnosis of hypertension, or current antihypertensive therapy. (iii) Low HDL-C (< 50 mg/dL). (iv) Hypertriglyceridaemia, defined as triglycerides ≥ 150 mg/dL, a physician diagnosis of dyslipidaemia, or current lipid-lowering medication. (v) Hyperglycaemia, defined as fasting glucose ≥ 100 mg/dL, physician-diagnosed diabetes, or glucose-lowering therapy. (vi) Abnormal sleep duration, defined as self-reported habitual sleep shorter than 6 h or longer than 9 h per night. (vii) Clinically relevant depressive symptomatology, indexed by a Center for Epidemiologic Studies Depression Scale (CES-D) score of ≥ 10 [32]. Respondents fulfilling at least four of the above at Wave 3 but fewer than four at Wave 1 were classified as incident CircS cases.

### Assessment of covariates

The covariate set encompassed sociodemographic attributes (age, sex, marital status, urban/rural residence, and educational attainment), health behaviours (smoking and alcohol intake), and clinical markers (BMI, hypertension, diabetes, and use of lipid-lowering drugs). Variable inclusion was guided by prior evidence in the CHARLS literature and by clinical plausibility rather than data-driven screening.

### Statistical analysis

Descriptive summaries are presented as mean (standard deviation) for continuous variables—with between-group comparisons carried out by Wilcoxon rank-sum test—and as frequencies with percentages for categorical variables, compared by Pearson's chi-square test. Because incident CircS was captured only at Wave 3 without a datable onset, risk ratios (RR) were estimated from modified Poisson models fitted with robust (sandwich) variance, an approach recommended for binary endpoints in cohort data [33]. Two progressively adjusted models were specified: Model 1 retained age and sex alone; Model 2 additionally controlled for marital status, residence, educational attainment, smoking, alcohol consumption, BMI, hypertension, diabetes, and lipid-lowering drug use. Each index entered the model both continuously (per 1 standard deviation) and as quartiles, with Q1 serving as the reference group for all indices except eGDR, where Q4 was the natural referent given its protective direction. Linear trend was evaluated by treating the ordinal quartile rank as a single continuous covariate.

Spearman rank correlation coefficients were calculated to assess collinearity among the eight indices. Restricted cubic splines with four knots (at the 5th, 35th, 65th, and 95th percentiles) were used to examine dose–response relationships, with the median as the reference value [34]. Overall and nonlinearity *P* values were obtained from likelihood ratio tests. Receiver operating characteristic analysis assessed the incremental predictive value of adding each index to a base covariate model; area under the curve (AUC) values were compared using DeLong tests, and continuous net reclassification improvement (NRI) and integrated discrimination improvement (IDI) were calculated [35].

Bidirectional mediation analysis was conducted using quasi-Bayesian approximation with 1,000 simulations [36]. The forward path tested whether hs-CRP mediated the association between each metabolic index and CircS; the reverse path tested whether metabolic indices mediated the association between hs-CRP and CircS. Biological-age acceleration, defined as the residual from a regression of chronological age on a panel of biomarkers, was examined as an additional mediator.

Sensitivity analyses included Cox proportional hazards models to confirm directional consistency, exclusion of participants with diabetes or hypertension to evaluate confounding by pre-existing conditions, inverse probability of treatment weighting (IPTW) to strengthen causal interpretation, and an additional covariate sensitivity analysis that progressively extended the fully adjusted model. In this extended-covariate framework, Model A corresponds to the primary fully adjusted model (equivalent to Model 2 in the main analysis); Model B additionally included self-reported heart disease, stroke, and lung disease; Model C further added physical activity status (binary: active [≥ 1 day/week of vigorous or moderate activity] vs inactive, derived from CHARLS International Physical Activity Questionnaire [IPAQ]-short questions [37]). The binary active-versus-inactive classification is a conservative operationalisation suitable for a sensitivity analysis rather than a primary exposure, as the CHARLS Wave 1 IPAQ-short items captured activity days without validated metabolic equivalent (MET)-minute-per-week quantification. Subgroup analyses with interaction testing were performed across strata of age, sex, BMI, hypertension, diabetes, smoking, and drinking.

Several metabolic indices share component variables with the CircS diagnostic criteria. TyG-BMI incorporates triglycerides and fasting glucose; METS-IR includes fasting glucose, triglycerides, BMI, and HDL-C; the CHG index uses total cholesterol, HDL-C, and fasting glucose; and eGDR is derived from waist circumference, HbA1c, and hypertension status. Because CircS is defined in part by thresholds of these components, structural overlap exists by design and may inflate estimated associations. To partially address this, we adjusted for BMI, hypertension, and diabetes in all multivariable models, conducted IPTW sensitivity analyses, and present full overlap documentation in Table S3.

Predictive benchmarking covered ten algorithms—logistic regression, naive Bayes, least absolute shrinkage and selection operator (LASSO)-penalised logistic regression, random forest, XGBoost, k-nearest neighbours, multilayer perceptron, LightGBM, decision tree, and a radial-basis-function support-vector machine—implemented on a 70/30 stratified training–testing partition. A LASSO feature-selection step confined to the training partition preceded model fitting; its L1 penalty (alpha = 1.0) was tuned through an independent tenfold cross-validation loop after centring and scaling (mean 0, SD 1) each continuous predictor. The lambda.min rule, which minimises tenfold cross-validated binomial deviance, was used to select the final feature set; variables with non-zero coefficients at lambda.min were retained, whereas variables shrunk exactly to zero were dropped. To preserve an unbiased estimate of generalisation error, the held-out test split remained completely separate from feature selection and all hyperparameter tuning. Algorithm-specific hyperparameters were tuned by fivefold cross-validation restricted to the training partition: for XGBoost the search spanned n_estimators (100–500), max_depth (3–8), learning rate (0.01–0.3), and subsample ratio (0.6–1.0); random forest parameters (n_estimators 100–500 and max_depth 5–20) were tuned analogously. For each metabolic index, we identified the cutoff maximising Youden's J (sensitivity + specificity − 1) on the receiver-operating-characteristic curve. Test-set figures for AUC, Brier score, sensitivity, specificity, and F1 are reported alongside 95% confidence intervals obtained from 2,000 bootstrap resamples of the test partition (*n* = 880). SHapley Additive exPlanations (SHAP) were computed on the XGBoost model rather than on the top-ranked logistic regression because gradient-boosted trees capture non-linearities and feature interactions that logistic regression cannot, and because XGBoost yields exact TreeSHAP values at low additional computational cost [29]; the narrow AUC gap between the two models (0.746 vs 0.721) and near-identical top-ranking features justified this choice. Model calibration and net-benefit utility were examined through calibration curves and decision-curve analysis [38]. All computations were carried out in R 4.4.1, with a two-sided *P* < 0.05 threshold applied throughout.

## Results

### Sample characteristics

Successive exclusions detailed in Fig. 1 and Table S1 left an analytic cohort of 4,325 respondents. Over 4 years of follow-up between Wave 1 (2011) and Wave 3 (2015), 1,025 individuals (23.7%) met CircS criteria for the first time. Table 1 displays baseline profiles stratified by subsequent CircS status. Mean age was comparable across the two strata (58.2 years; *P* = 0.820). Respondents who later developed CircS carried heavier metabolic burdens at baseline: higher BMI (24.14 versus 22.04 kg/m2; *P* < 0.001), larger waists (86.56 versus 80.42 cm; *P* < 0.001), higher triglycerides (122.38 versus 96.17 mg/dL; *P* < 0.001), higher fasting glucose (107.17 versus 102.13 mg/dL; *P* < 0.001), higher HbA1c (5.25% versus 5.13%;* P* < 0.001), higher hs-CRP (2.55 versus 2.42 mg/L; *P* < 0.001), and lower HDL-C (50.29 versus 57.53 mg/dL; *P* < 0.001). Urban residents were overrepresented among incident cases (35.6% versus 30.3%; *P* = 0.002), whereas sex, education, marital status, smoking, and alcohol intake did not differ appreciably.

**Table 1:** Baseline characteristics of participants by incident circadian syndrome status

**Variable**	**N (No CircS)**	**N (CircS)**	**No CircS**	**CircS**	***P* value**	**Total (No CircS)**	**Total (CircS)**
Age, years	3300	1025	58.22 (8.67)	58.16 (8.79)	0.820	3300	1025
BMI, kg/m2	2924	823	22.04 (3.32)	24.14 (3.44)	<0.001	3300	1025
Waist circumference, cm	2885	812	80.42 (8.33)	86.56 (8.87)	<0.001	3300	1025
Triglycerides, mg/dL	3231	986	96.17 (50.43)	122.38 (84.76)	<0.001	3300	1025
HDL-C, mg/dL	3233	986	57.53 (15.01)	50.29 (13.21)	<0.001	3300	1025
Fasting glucose, mg/dL	3226	985	102.13 (24.66)	107.17 (32.42)	<0.001	3300	1025
HbA1c, %	3275	1015	5.13 (0.61)	5.25 (0.80)	<0.001	3300	1025
hs-CRP, mg/L	3232	986	2.42 (8.13)	2.55 (5.50)	<0.001	3300	1025
AIP	3230	986	0.20 (0.25)	0.34 (0.27)	<0.001	3300	1025
CHG Index	2906	885	5.12 (0.30)	5.31 (0.35)	<0.001	3300	1025
RCII	3228	986	3.70 (9.77)	5.37 (10.57)	<0.001	3300	1025
hs-CRP/HDL-C	3232	986	0.04 (0.11)	0.05 (0.11)	<0.001	3300	1025
CTI	3226	985	8.37 (0.65)	8.70 (0.73)	<0.001	3300	1025
TyG-BMI	2858	787	184.72 (29.97)	207.10 (32.46)	<0.001	3300	1025
eGDR	2847	798	10.61 (1.53)	9.52 (1.85)	<0.001	3300	1025
METS-IR	2853	784	2.19 (0.17)	2.30 (0.19)	<0.001	3300	1025
Sex: Female	3300	1025	1685 (51.1%)	557 (54.3%)	0.072	3300	1025
Sex: Male	3300	1025	1615 (48.9%)	468 (45.7%)	0.072	3300	1025
Education: Elementary or below	3300	1025	2296 (69.6%)	681 (66.4%)	0.064	3300	1025
Education: Middle school or above	3300	1025	1004 (30.4%)	344 (33.6%)	0.064	3300	1025
Marital status: Married/Partnered	3300	1025	2973 (90.1%)	919 (89.7%)	0.348	3300	1025
Marital status: Separated/Divorced	3300	1025	29 (0.9%)	5 (0.5%)	0.348	3300	1025
Marital status: Widowed/Never married	3300	1025	298 (9.0%)	101 (9.9%)	0.348	3300	1025
Residence: Rural	3300	1025	2300 (69.7%)	660 (64.4%)	0.002	3300	1025
Residence: Urban	3300	1025	1000 (30.3%)	365 (35.6%)	0.002	3300	1025
Smoking: Non-smoker	3300	1025	1918 (58.1%)	621 (60.6%)	0.283	3300	1025
Smoking: Smoker	3300	1025	1380 (41.8%)	404 (39.4%)	0.283	3300	1025
Smoking: Unknown	3300	1025	2 (0.1%)	0 (0.0%)	0.283	3300	1025
Drinking: Drinker	3300	1025	979 (29.7%)	285 (27.8%)	0.407	3300	1025
Drinking: Non-drinker	3300	1025	2130 (64.5%)	685 (66.8%)	0.407	3300	1025
Drinking: Unknown	3300	1025	191 (5.8%)	55 (5.4%)	0.407	3300	1025

Data are presented as mean (SD) for continuous variables and n (%) for categorical variables. *P* values were obtained from Wilcoxon rank-sum tests for continuous variables and chi-square tests for categorical variables

*Abbreviations: BMI* body mass index, *CircS* circadian syndrome, *HDL-C* high-density lipoprotein cholesterol, *hs-CRP* high-sensitivity C-reactive protein, *HbA1c* glycated hemoglobin, *AIP* atherogenic index of plasma, *CHG* cholesterol-HDL-glucose index, *RCII* remnant cholesterol inflammatory index, *CTI* C-reactive protein triglyceride glucose index, *TyG-BMI* triglyceride-glucose body mass index, *eGDR* estimated glucose disposal rate, *METS-IR* metabolic score for insulin resistance

### Dose–response relationships

**Fig. 2:**
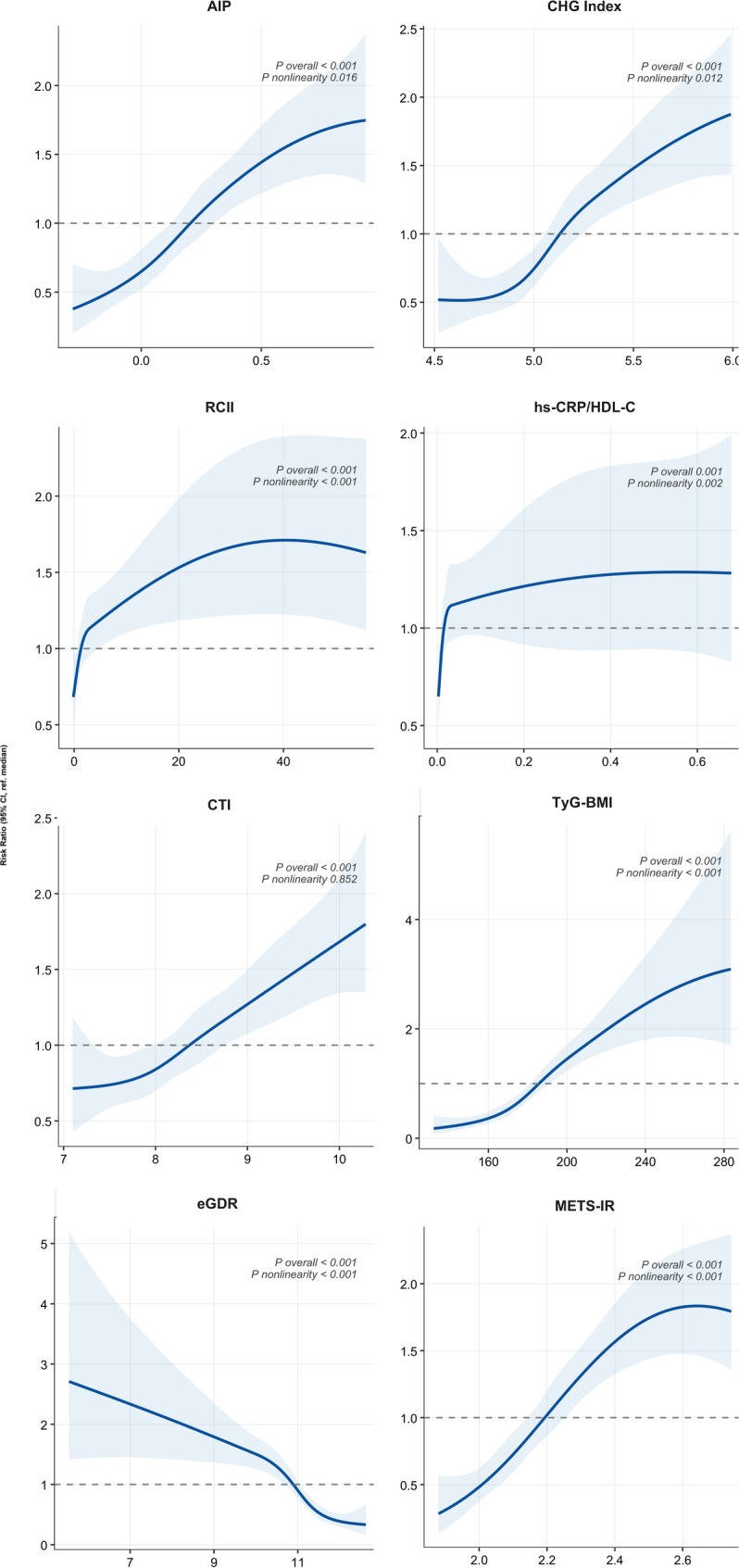
Restricted cubic spline associations of eight metabolic indices with incident circadian syndrome. Each panel shows the adjusted risk ratio (solid line) and 95% confidence interval (shaded area) for one metabolic index. Splines used four knots placed at the 5th, 35th, 65th, and 95th percentiles. The reference point (risk ratio = 1) was set at the median value. Models were adjusted for age, sex, marital status, residence, education, smoking, drinking, BMI, hypertension, diabetes, and lipid-lowering medication use

Restricted cubic spline analyses showed significant dose–response associations between all eight metabolic indices and incident CircS (all P overall < 0.001; Fig. 2; Table S4). CTI exhibited an approximately linear positive association (P nonlinearity = 0.852), whereas TyG-BMI, eGDR, METS-IR, RCII, and hs-CRP/HDL-C showed significant nonlinear patterns (all P nonlinearity < 0.05). Detailed nonlinearity *P* values and grid values for all indices are provided in Tables S4 and S5, respectively; Spearman rank correlations among the eight indices are shown in Table S6 and Fig. S1. eGDR showed a characteristic inverse curve consistent with its role as an insulin sensitivity marker, with the risk of CircS declining as values rose above the median. TyG-BMI showed a steep increase at higher values, suggesting a threshold effect. AIP showed borderline nonlinearity (P nonlinearity = 0.016) that was visually near-linear.

### Association between metabolic indices and incident circs

Within Model 2 (full adjustment), every index retained a statistically significant link with incident CircS on the per-SD scale (Table 2). The steepest positive gradient belonged to TyG-BMI (RR 1.87 per SD, 95% CI 1.66–2.10), with the CHG Index (RR 1.37, 95% CI 1.30–1.45) and AIP (RR 1.36, 95% CI 1.29–1.43) next in magnitude. The strongest protective association was observed for eGDR (per-SD RR 0.54, 95% CI 0.47–0.62). The remaining indices—METS-IR (RR 1.34, 95% CI 1.28–1.42), CTI (RR 1.25, 95% CI 1.18–1.33), RCII (RR 1.09, 95% CI 1.04–1.14), and the hs-CRP/HDL-C ratio (RR 1.06, 95% CI 1.01–1.12)—likewise showed significant positive associations with incident CircS.

**Table 2:** Associations of eight metabolic indices with incident circadian syndrome per standard deviation increase

**Outcome**	**Index**	**Model**	**RR**	**Lower CI**	**Upper CI**	***P* value**	**N**	**Events**	**Index label**
event	AIP	M1	1.462	1.401	1.525	<0.001	4216	986	AIP
event	AIP	M2	1.356	1.285	1.430	<0.001	3356	725	AIP
event	CHG Index	M1	1.470	1.410	1.532	<0.001	3791	885	CHG Index
event	CHG Index	M2	1.373	1.298	1.452	<0.001	3004	648	CHG Index
event	RCII	M1	1.101	1.062	1.141	<0.001	4214	986	RCII
event	RCII	M2	1.092	1.044	1.142	<0.001	3354	725	RCII
event	hs-CRP/HDL-C	M1	1.074	1.033	1.117	<0.001	4218	986	hs-CRP/HDL-C
event	hs-CRP/HDL-C	M2	1.064	1.009	1.122	0.021	3358	725	hs-CRP/HDL-C
event	CTI	M1	1.388	1.326	1.452	<0.001	4211	985	CTI
event	CTI	M2	1.254	1.183	1.330	<0.001	3353	725	CTI
event	TyG-BMI	M1	1.568	1.498	1.641	<0.001	3645	787	TyG-BMI
event	TyG-BMI	M2	1.865	1.659	2.097	<0.001	3353	725	TyG-BMI
event	eGDR	M1	0.669	0.640	0.698	<0.001	3645	798	eGDR
event	eGDR	M2	0.540	0.470	0.620	<0.001	3345	729	eGDR
event	METS-IR	M1	1.467	1.399	1.538	<0.001	3637	784	METS-IR
event	METS-IR	M2	1.344	1.276	1.415	<0.001	3353	725	METS-IR

Model 1 adjusted for age and sex. Model 2 additionally adjusted for marital status, residence, education, smoking, drinking, BMI, hypertension, diabetes, and lipid-lowering medication use

*Abbreviations: CI* confidence interval, *RR* risk ratio, *SD* standard deviation

**Fig. 3:**
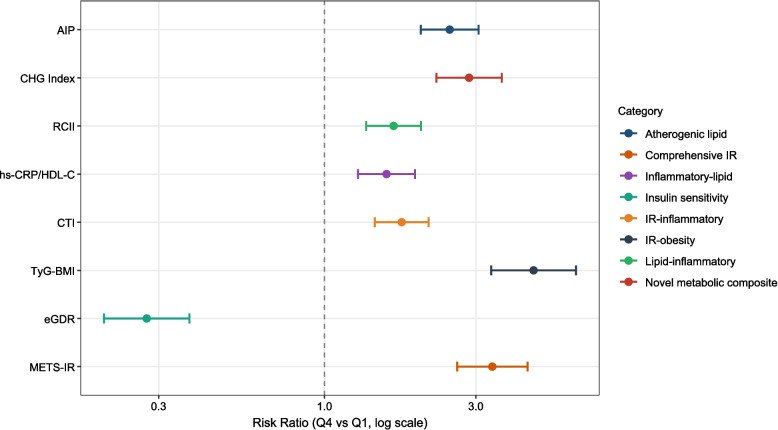
Quartile-based associations of eight metabolic indices with incident circadian syndrome. Risk ratios and 95% confidence intervals are shown for Q4 versus Q1 (Q1 vs Q4 for eGDR) from the fully adjusted model. The vertical dashed line indicates the null value (risk ratio = 1). All P for trend values were below 0.001. Abbreviations: AIP, atherogenic index of plasma; CHG, cholesterol-HDL-glucose index; CircS, circadian syndrome; CTI, C-reactive protein triglyceride glucose index; eGDR, estimated glucose disposal rate; METS-IR, metabolic score for insulin resistance; RCII, remnant cholesterol inflammatory index; TyG-BMI, triglyceride-glucose body mass index

Analyses by quartile reinforced these dose–response gradients for every index (Table 3; Fig. 3). When comparing the top and bottom quartiles, TyG-BMI produced the greatest effect size (Q4 vs Q1 RR 4.56, 95% CI 3.35–6.21), with METS-IR (RR 3.38, 95% CI 2.62–4.37), the CHG Index (RR 2.86, 95% CI 2.25–3.62), AIP (RR 2.48, 95% CI 2.01–3.06), CTI (RR 1.75, 95% CI 1.44–2.13), RCII (RR 1.65, 95% CI 1.35–2.02), and the hs-CRP/HDL-C ratio (RR 1.57, 95% CI 1.28–1.93) following. By contrast, participants in the highest eGDR quartile carried a 72% lower risk than those in the lowest quartile (RR 0.28, 95% CI 0.20–0.38). All P for trend values fell below 0.001.

**Table 3:** Associations of eight metabolic indices with incident circadian syndrome by quartiles

**Outcome**	**Index**	**Model**	**Quartile**	**RR**	**Lower CI**	**Upper CI**	***P* value**	**P trend**	**N**	**Events**	**N per quartile**	**Events per quartile**	**Risk (%)**	**Index label**
event	AIP	M1	Q2	1.382	1.120	1.704	0.003	<0.001	4216	986	1054	179	16.983	AIP
event	AIP	M1	Q3	2.172	1.796	2.628	<0.001	<0.001	4216	986	1054	281	26.660	AIP
event	AIP	M1	Q4	3.081	2.574	3.688	<0.001	<0.001	4216	986	1054	397	37.666	AIP
event	AIP	M2	Q2	1.288	1.012	1.641	0.040	<0.001	3356	725	839	129	15.375	AIP
event	AIP	M2	Q3	2.084	1.678	2.589	<0.001	<0.001	3356	725	839	219	26.103	AIP
event	AIP	M2	Q4	2.482	2.012	3.060	<0.001	<0.001	3356	725	839	282	33.611	AIP
event	CHG Index	M1	Q2	1.595	1.269	2.006	<0.001	<0.001	3791	885	948	165	17.405	CHG Index
event	CHG Index	M1	Q3	2.396	1.939	2.960	<0.001	<0.001	3791	885	947	248	26.188	CHG Index
event	CHG Index	M1	Q4	3.578	2.932	4.366	<0.001	<0.001	3791	885	948	369	38.924	CHG Index
event	CHG Index	M2	Q2	1.444	1.105	1.887	0.007	<0.001	3004	648	751	118	15.712	CHG Index
event	CHG Index	M2	Q3	2.261	1.768	2.892	<0.001	<0.001	3004	648	751	193	25.699	CHG Index
event	CHG Index	M2	Q4	2.857	2.253	3.623	<0.001	<0.001	3004	648	751	263	35.020	CHG Index
event	RCII	M1	Q2	1.317	1.091	1.590	0.004	<0.001	4214	986	1053	208	19.753	RCII
event	RCII	M1	Q3	1.712	1.434	2.043	<0.001	<0.001	4214	986	1053	269	25.546	RCII
event	RCII	M1	Q4	2.240	1.895	2.648	<0.001	<0.001	4214	986	1054	351	33.302	RCII
event	RCII	M2	Q2	1.236	0.999	1.530	0.051	<0.001	3354	725	838	160	19.093	RCII
event	RCII	M2	Q3	1.414	1.153	1.734	0.001	<0.001	3354	725	838	195	23.270	RCII
event	RCII	M2	Q4	1.652	1.353	2.016	<0.001	<0.001	3354	725	839	251	29.917	RCII
event	hs-CRP/HDL-C	M1	Q2	1.459	1.209	1.761	<0.001	<0.001	4218	986	1054	222	21.063	hs-CRP/HDL-C
event	hs-CRP/HDL-C	M1	Q3	1.894	1.585	2.262	<0.001	<0.001	4218	986	1054	285	27.040	hs-CRP/HDL-C
event	hs-CRP/HDL-C	M1	Q4	2.178	1.831	2.591	<0.001	<0.001	4218	986	1055	326	30.900	hs-CRP/HDL-C
event	hs-CRP/HDL-C	M2	Q2	1.314	1.061	1.628	0.012	<0.001	3358	725	839	167	19.905	hs-CRP/HDL-C
event	hs-CRP/HDL-C	M2	Q3	1.491	1.214	1.831	<0.001	<0.001	3358	725	839	207	24.672	hs-CRP/HDL-C
event	hs-CRP/HDL-C	M2	Q4	1.569	1.276	1.929	<0.001	<0.001	3358	725	840	234	27.857	hs-CRP/HDL-C
event	CTI	M1	Q2	1.194	0.982	1.452	0.075	<0.001	4211	985	1053	186	17.664	CTI
event	CTI	M1	Q3	1.651	1.378	1.977	<0.001	<0.001	4211	985	1052	257	24.430	CTI
event	CTI	M1	Q4	2.480	2.101	2.928	<0.001	<0.001	4211	985	1053	386	36.657	CTI
event	CTI	M2	Q2	1.104	0.886	1.376	0.378	<0.001	3353	725	838	141	16.826	CTI
event	CTI	M2	Q3	1.429	1.166	1.752	0.001	<0.001	3353	725	838	192	22.912	CTI
event	CTI	M2	Q4	1.752	1.442	2.130	<0.001	<0.001	3353	725	838	271	32.339	CTI
event	TyG-BMI	M1	Q2	1.939	1.480	2.542	<0.001	<0.001	3645	787	911	135	14.819	TyG-BMI
event	TyG-BMI	M1	Q3	3.240	2.524	4.158	<0.001	<0.001	3645	787	911	221	24.259	TyG-BMI
event	TyG-BMI	M1	Q4	5.355	4.225	6.788	<0.001	<0.001	3645	787	911	359	39.407	TyG-BMI
event	TyG-BMI	M2	Q2	1.971	1.473	2.637	<0.001	<0.001	3353	725	838	127	15.155	TyG-BMI
event	TyG-BMI	M2	Q3	3.118	2.354	4.130	<0.001	<0.001	3353	725	838	205	24.463	TyG-BMI
event	TyG-BMI	M2	Q4	4.562	3.354	6.207	<0.001	<0.001	3353	725	838	331	39.499	TyG-BMI
event	eGDR	M1	Q2	0.706	0.615	0.810	<0.001	<0.001	3645	798	914	243	26.586	eGDR
event	eGDR	M1	Q3	0.414	0.348	0.492	<0.001	<0.001	3645	798	908	141	15.529	eGDR
event	eGDR	M1	Q4	0.199	0.156	0.255	<0.001	<0.001	3645	798	906	68	7.506	eGDR
event	eGDR	M2	Q2	0.802	0.660	0.974	0.026	<0.001	3345	729	837	224	26.762	eGDR
event	eGDR	M2	Q3	0.532	0.421	0.673	<0.001	<0.001	3345	729	835	132	15.808	eGDR
event	eGDR	M2	Q4	0.275	0.202	0.375	<0.001	<0.001	3345	729	836	62	7.416	eGDR
event	METS-IR	M1	Q2	2.021	1.566	2.608	<0.001	<0.001	3637	784	909	158	17.382	METS-IR
event	METS-IR	M1	Q3	2.809	2.206	3.577	<0.001	<0.001	3637	784	909	218	23.982	METS-IR
event	METS-IR	M1	Q4	4.300	3.417	5.411	<0.001	<0.001	3637	784	909	330	36.304	METS-IR
event	METS-IR	M2	Q2	1.964	1.505	2.562	<0.001	<0.001	3353	725	838	149	17.780	METS-IR
event	METS-IR	M2	Q3	2.539	1.961	3.287	<0.001	<0.001	3353	725	838	201	23.986	METS-IR
event	METS-IR	M2	Q4	3.381	2.619	4.365	<0.001	<0.001	3353	725	838	306	36.516	METS-IR

Q1 served as the reference group for all indices except eGDR, for which Q4 was the reference. Model 2 adjusted for age, sex, marital status, residence, education, smoking, drinking, BMI, hypertension, diabetes, and lipid-lowering medication use

*Abbreviations: CI* confidence interval, *RR* risk ratio

### Incremental predictive value

**Fig. 4:**
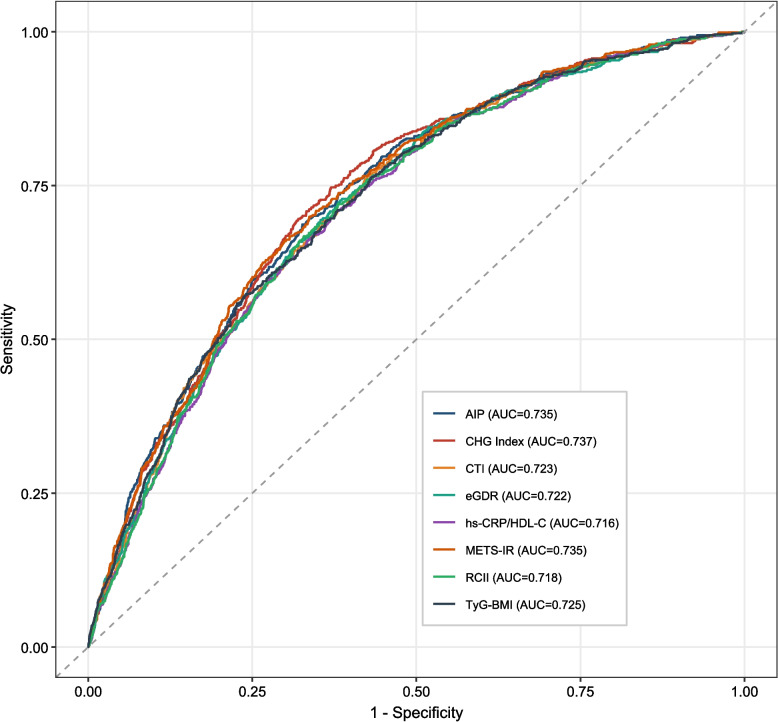
Incremental discrimination after adding each metabolic index to the base model for incident circadian syndrome. The base model included age, sex, BMI, marital status, residence, education, smoking, drinking, hypertension, diabetes, and lipid-lowering medication use. Each coloured line represents one augmented model, and DeLong *P* values compare the augmented model with the base model. Abbreviations: AUC, area under the receiver operating characteristic curve

When the metabolic indices were layered on top of the base covariate model (baseline AUC 0.713), discrimination improved for most scores (Table 4; Fig. 4). The CHG index produced the largest incremental AUC (0.737, 95% CI 0.717–0.757; DeLong *P* < 0.001), with AIP (AUC 0.735; DeLong *P* = 0.001) and METS-IR (AUC 0.735; DeLong *P* < 0.001) close behind. Continuous NRI spanned 0.136 (hs-CRP/HDL-C) to 0.392 (AIP), and IDI ranged from 0.001 to 0.030. Six of the eight indices crossed the DeLong significance threshold (*P* < 0.05); only hs-CRP/HDL-C (*P* = 0.074) and CTI (*P* = 0.052) fell short. The observed incremental contribution mirrors results from multi-platform metabolomic prediction work, in which composite metabolic scores have refined risk stratification beyond conventional factors for coronary artery disease and type 2 diabetes [39]; similarly, nuclear magnetic resonance (NMR)-derived metabolomic fingerprints from the UK Biobank outperformed established clinical predictors across a panel of 24 prevalent diseases followed for 10 years [40]. Our findings broaden this paradigm by showing that easily obtainable, biochemistry-based composite indices can likewise sharpen prediction of a multi-component syndromic phenotype.

**Table 4:** Incremental predictive value of eight metabolic indices added to the base model for incident circadian syndrome

**Index**	**Index label**	**N**	**Events**	**AUC (base model)**	**AUC base lower CI**	**AUC base upper CI**	**AUC (base + index)**	**AUC full lower CI**	**AUC full upper CI**	**DeLong P**	**NRI (continuous)**	**IDI**
AIP	AIP	3356	725	0.713	0.691	0.733	0.735	0.717	0.756	0.001	0.392	0.028
CHG Index	CHG Index	3004	648	0.713	0.694	0.733	0.737	0.717	0.757	0.001	0.379	0.030
RCII	RCII	3354	725	0.713	0.691	0.732	0.718	0.697	0.739	0.026	0.162	0.003
hs-CRP/HDL-C	hs-CRP/HDL-C	3358	725	0.712	0.693	0.732	0.716	0.695	0.735	0.074	0.136	0.001
CTI	CTI	3353	725	0.713	0.692	0.732	0.723	0.701	0.743	0.052	0.290	0.014
TyG-BMI	TyG-BMI	3353	725	0.713	0.689	0.732	0.725	0.705	0.745	0.021	0.342	0.022
eGDR	eGDR	3345	729	0.709	0.690	0.730	0.722	0.702	0.741	0.009	0.236	0.016
METS-IR	METS-IR	3353	725	0.713	0.692	0.732	0.735	0.718	0.755	<0.001	0.379	0.029

The base model included age, sex, marital status, residence, education, smoking, drinking, BMI, hypertension, diabetes, and lipid-lowering medication use. DeLong *P* values compare the augmented model to the base model

*Abbreviations: AUC* area under the receiver operating characteristic curve, *CI* confidence interval, *IDI* integrated discrimination improvement, *NRI* net reclassification improvement

### Mediation and biological ageing analysis

**Fig. 5:**
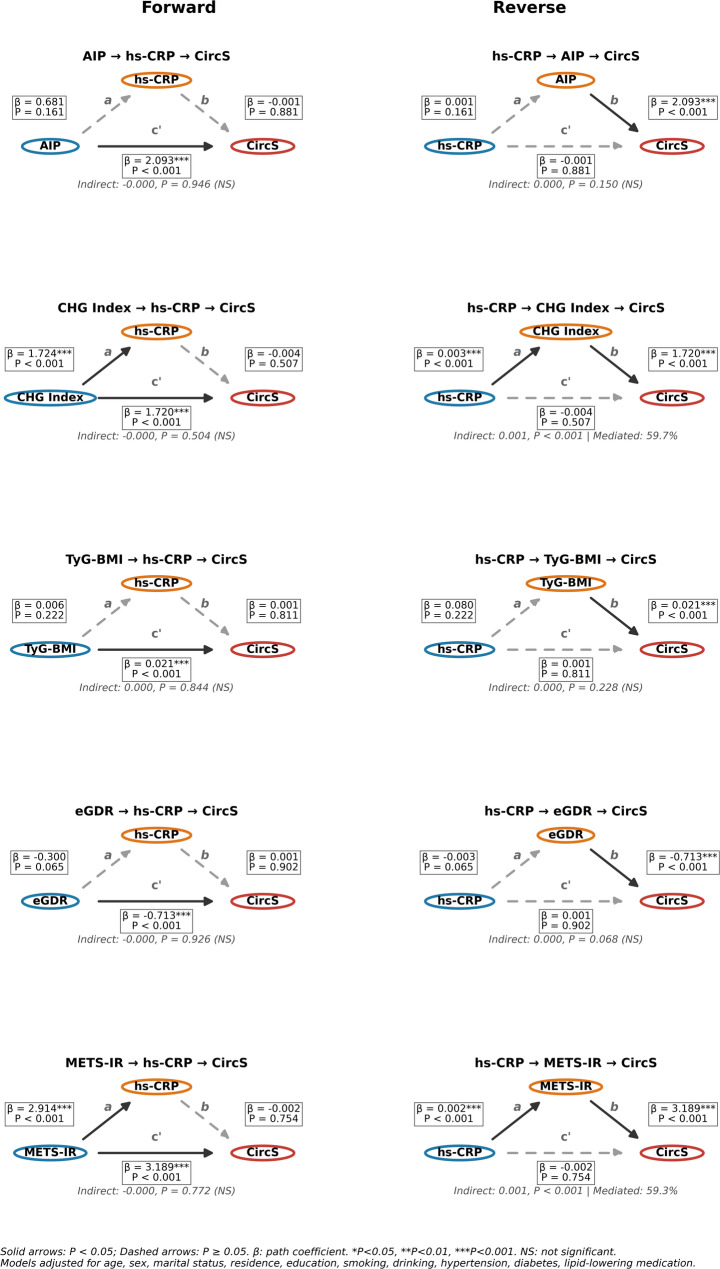
Bidirectional mediation of the hs-CRP-circadian syndrome association by metabolic indices. The forward path (metabolic index—> hs-CRP—> circadian syndrome) and reverse path (hs-CRP—> metabolic index—> circadian syndrome) are shown. Bar height represents the average causal mediation effect. Abbreviations: ACME, average causal mediation effect; hs-CRP, high-sensitivity C-reactive protein

The bidirectional mediation framework probed whether hs-CRP sits between the metabolic indices and CircS (Table 5; Fig. 5). On the forward pathway (index → hs-CRP → CircS), no index produced a statistically significant indirect effect (all average causal mediation effect [ACME] *P* > 0.05), consistent with hs-CRP not acting as an intermediate for these associations. Reversing the causal ordering (hs-CRP → index → CircS) changed the picture: both the CHG index and METS-IR produced clearly significant indirect effects (both ACME *P* < 0.001), pointing toward a model in which systemic inflammation feeds into CircS risk partly by perturbing atherogenic lipid handling and insulin homeostasis.

**Table 5:** Bidirectional mediation analysis of hs-CRP in associations between metabolic indices and incident circadian syndrome

**Direction**	**Index**	**Index label**	**Mediator**	**N**	**Events**	**ACME**	**ACME lower CI**	**ACME upper CI**	**ACME P**	**ADE**	**ADE lower CI**	**ADE upper CI**	**ADE P**	**Total effect**	**Total lower CI**	**Total upper CI**	**Total P**	**Proportion mediated**	**Prop. lower CI**	**Prop. upper CI**	**Prop. P**
Index → hs-CRP → CircS	AIP	AIP	hs-CRP	3892	909	-<0.001	−0.001	0.001	0.910	0.419	0.357	0.483	<0.001	0.419	0.358	0.483	<0.001	-<0.001	−0.003	0.002	0.910
Index → hs-CRP → CircS	CHG Index	CHG Index	hs-CRP	3505	823	-<0.001	-<0.001	<0.001	0.476	<0.001	<0.001	0.001	<0.001	<0.001	<0.001	0.001	<0.001	−0.001	−0.006	0.003	0.476
Index → hs-CRP → CircS	TyG-BMI	TyG-BMI	hs-CRP	3361	728	<0.001	-<0.001	<0.001	0.834	<0.001	<0.001	<0.001	<0.001	<0.001	<0.001	<0.001	<0.001	<0.001	−0.004	0.005	0.834
Index → hs-CRP → CircS	eGDR	eGDR	hs-CRP	3295	713	-<0.001	-<0.001	<0.001	0.912	−0.004	−0.007	−0.002	<0.001	−0.004	−0.007	−0.002	<0.001	<0.001	−0.004	0.004	0.912
Index → hs-CRP → CircS	METS-IR	METS-IR	hs-CRP	3353	725	-<0.001	-<0.001	<0.001	0.744	0.006	0.003	0.009	<0.001	0.006	0.003	0.009	<0.001	-<0.001	−0.002	0.001	0.744
hs-CRP → Index → CircS	hs-CRP	-	AIP	3892	909	<0.001	-<0.001	0.001	0.162	-<0.001	−0.002	0.002	0.872	<0.001	−0.002	0.002	0.914	0.094	−5.425	4.123	0.888
hs-CRP → Index → CircS	hs-CRP	-	CHG Index	3505	823	0.001	<0.001	0.001	<0.001	−0.001	−0.002	0.001	0.484	<0.001	−0.002	0.002	0.830	0.569	−12.357	11.748	0.830
hs-CRP → Index → CircS	hs-CRP	-	TyG-BMI	3361	728	<0.001	-<0.001	0.001	0.212	<0.001	−0.001	0.002	0.784	<0.001	−0.001	0.002	0.586	0.201	−2.986	3.802	0.574
hs-CRP → Index → CircS	hs-CRP	-	eGDR	3295	713	<0.001	-<0.001	0.001	0.072	<0.001	−0.002	0.002	0.870	0.001	−0.001	0.002	0.550	0.308	−4.629	6.783	0.546
hs-CRP → Index → CircS	hs-CRP	-	METS-IR	3353	725	0.001	<0.001	0.001	<0.001	-<0.001	−0.002	0.001	0.686	<0.001	−0.001	0.002	0.624	0.629	−10.873	14.149	0.624

Mediation analysis was performed using quasi-Bayesian approximation with 1,000 simulations. The forward path examined hs-CRP as a mediator of the index-CircS association; the reverse path examined metabolic indices as mediators of the hs-CRP-CircS association

*Abbreviations: ACME* average causal mediation effect, *ADE* average direct effect, *CircS* circadian syndrome, *hs-CRP* high-sensitivity C-reactive protein

Biological-age acceleration significantly mediated the association between metabolic indices and CircS (Table S7). The mediated proportion ranged from 3.9% for TyG-BMI (*P* < 0.001) to 42.8% for eGDR (*P* = 0.014), with CTI at 6.6% (*P* < 0.001). The marked disparity in mediation proportions deserves further comment. The eGDR formula incorporates waist circumference, HbA1c, and hypertension status—three variables that feature prominently among recognised biomarkers of biological ageing. Waist circumference reflects visceral adiposity, a driver of chronic inflammation and cellular senescence; HbA1c captures cumulative glycaemic exposure that accelerates advanced glycation end-product accumulation; and hypertension is an established accelerator of vascular ageing. This compositional overlap with the biological-age construct explains the large mediated proportion. By contrast, TyG-BMI integrates triglycerides, fasting glucose, and BMI—a cruder proxy for body composition—yielding lower overlap with biological-age algorithms. From a biological perspective, insulin sensitivity (reflected by eGDR) may exert broader systemic effects on ageing pathways, including mitochondrial function, autophagy, and telomere maintenance, than the more narrowly lipid-glucose-focused perturbation captured by TyG-BMI. However, we cannot exclude that the large mediated proportion for eGDR partly reflects mathematical coupling rather than a genuine biological pathway.

### Sensitivity and subgroup analyses

Cox proportional hazards models yielded directionally consistent results for all eight indices (Table S8). TyG-BMI showed the largest per-SD hazard ratio (HR) (2.10, 95% CI 1.79—2.46), and eGDR showed the strongest inverse HR (0.49, 95% CI 0.42—0.58). In the extended covariate sensitivity analysis (Table S9), Model B (additionally adjusted for heart disease, stroke, and lung disease) yielded virtually identical risk ratios (e.g. TyG-BMI RR 1.874 vs 1.883; eGDR RR 0.541 vs 0.540). Model C (further adjusted for physical activity, *n* ≈ 1,400 with available data) confirmed that seven of eight indices remained significantly associated with incident CircS (all *P* < 0.001 except hs-CRP/HDL-C, *P* = 0.140), with comparable effect sizes (e.g. TyG-BMI RR 1.950, eGDR RR 0.565), indicating that results were robust to residual confounding by physical activity and prevalent comorbidities. IPTW analysis confirmed significant associations for AIP (odds ratio [OR] 1.92, 95% CI 1.72—2.16), the CHG index (OR 2.06, 95% CI 1.82—2.32), and eGDR (OR 0.32, 95% CI 0.27—0.37) after propensity-score weighting (Table S10), with covariate balance before and after weighting shown in Table S11.

Stratified analyses demonstrated that the observed associations were stable across age, sex, BMI, hypertension, diabetes, smoking, and alcohol strata, with no material interaction in the majority of comparisons (Table S12; Fig. S2). One notable exception emerged in the BMI-stratified analysis, where AIP displayed a significant interaction (P interaction = 0.004): the AIP–CircS link was stronger among respondents with BMI < 28 kg/m2 (RR 1.32, 95% CI 1.25–1.40) than among those with BMI ≥ 28 kg/m2 (RR 1.24, 95% CI 1.06–1.44). Additional sensitivity analyses that removed participants with diabetes, hypertension, or glucose-lowering medication produced concordant estimates for every index (Table S13).

### Machine learning analysis

**Fig. 6:**
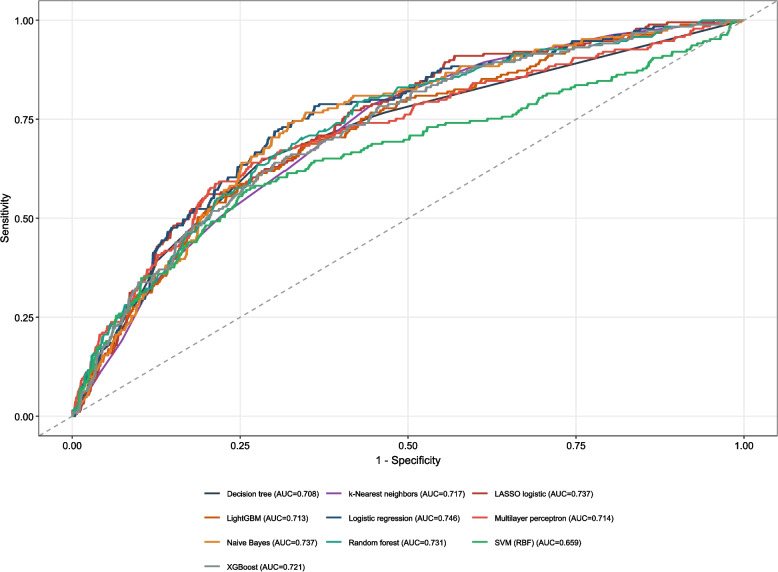
Receiver operating characteristic curves of 10 machine-learning models for incident circadian syndrome prediction. Receiver operating characteristic curves are shown for the independent test set (*n* = 880). The area under the curve with 95% confidence interval is displayed for each model. Logistic regression achieved the highest area under the curve

On the held-out test sample (*n* = 880; 189 incident events), the ten algorithms ranked as follows. Logistic regression attained the top area under the curve (AUC 0.746, 95% CI 0.710–0.784), followed by naive Bayes (0.737), LASSO logistic regression (0.737), and random forest (0.731; Fig. 6; Table S14). More elaborate algorithms—XGBoost (0.721), LightGBM (0.713), and the support-vector machine (0.659)—did not surpass the logistic baseline. Calibration plots showed acceptable agreement between predicted and observed probabilities (Fig. S3), and decision-curve analysis demonstrated that the logistic model delivered the greatest net benefit across a broad span of threshold probabilities (Fig. S4).

LASSO regularisation selected 13 of 21 candidate features (lambda.min = 0.003; Fig. S5). METS-IR received the largest coefficient (1.028), followed by AIP (0.608) and the CHG index (0.556). Three indices (RCII, CTI, and TyG-BMI) were not selected, likely because of multicollinearity with retained variables (Table S15). Among the eight metabolic indices, TyG-BMI had the highest discriminative ability for incident CircS (AUC 0.710, 95% CI 0.690–0.729), with an optimal cutoff of 184.306 (sensitivity 76.4%, specificity 55.2%; Youden index 0.315 [41]), followed by eGDR (AUC 0.702; cutoff 10.818; sensitivity 70.8%, specificity 60.6%; Youden index 0.314). Full cutoff values for all eight indices are presented in Table S16 and visualised in Fig. S6.

**Fig. 7:**
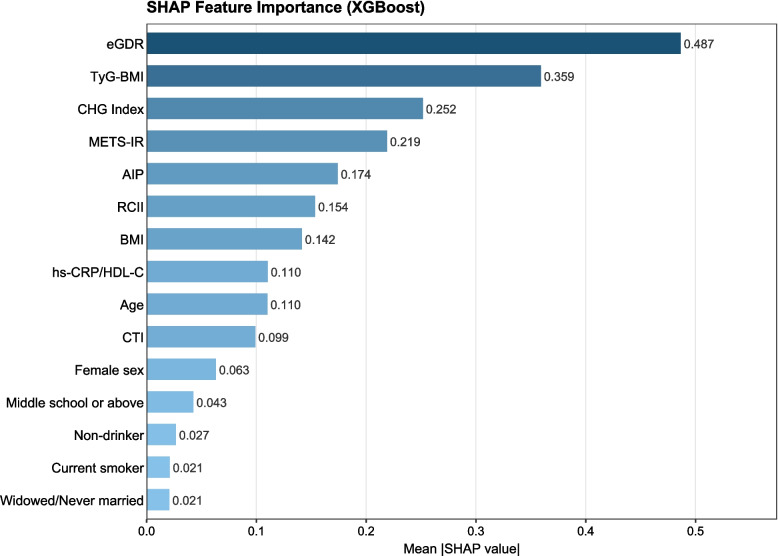
SHAP feature importance from the XGBoost model for incident circadian syndrome prediction. Mean absolute SHAP values are shown for the ranked predictors. eGDR had the highest mean absolute SHAP value, followed by TyG-BMI and the CHG index. Abbreviations: SHAP, Shapley additive explanations

When SHAP attributions were computed from the XGBoost model, eGDR emerged as the top-ranked predictor (mean absolute SHAP value 0.487), trailed by TyG-BMI (0.359), the CHG index (0.252), and METS-IR (0.219; Table S17; Fig. 7; Fig. S7). The beeswarm visualisation indicated that reductions in eGDR translated into positive SHAP contributions (i.e. higher predicted risk), whereas elevated eGDR values pushed contributions negative (i.e. lower predicted risk). BMI itself occupied only the seventh position (0.142), below every metabolic exposure index other than hs-CRP/HDL-C.

## Discussion

The present analysis profiled the prospective relationship between eight composite metabolic indices and incident CircS in a population-representative Chinese cohort. A recently published cross-sectional study examined insulin-resistance surrogates and CircS using dose–response modelling [42]; our work extends that line of enquiry by adopting a prospective design with 4-year follow-up, by simultaneously assessing eight indices across four pathophysiological domains, by embedding bidirectional mediation anchored on biological-age acceleration, and by benchmarking ten machine-learning algorithms with SHAP-based interpretability. Among 4,325 participants, three findings stood out. First, every index remained independently associated with incident CircS, with TyG-BMI and eGDR generating the largest effect sizes. Second, the CHG index contributed the largest gain in predictive discrimination beyond conventional risk factors. Third, the machine-learning pipeline coupled with SHAP attribution ranked eGDR as the leading predictor, yet logistic regression outperformed all nine more elaborate algorithms on the held-out test set.

Linkages between insulin-resistance composites and incident CircS are mechanistically coherent. TyG-BMI, which fuses fasting triglyceride–glucose coupling with body habitus, produced the steepest risk gradient (Q4 vs Q1 RR 4.56), aligning with existing reports that TyG-derived scores anticipate hypertension [43], adverse cardiovascular events [44, 45], and stroke [46]. TyG-BMI and eGDR address complementary facets of glucose–lipid–adiposity biology: TyG-BMI combines fasting triglyceride–glucose interaction with overall adiposity to proxy insulin resistance, while eGDR synthesises glycaemic control (HbA1c), body-fat distribution (waist circumference), and vascular status (hypertension) to approximate residual insulin sensitivity. Their opposite directions of association (positive for TyG-BMI, inverse for eGDR) therefore emerge naturally from biology, and joint deployment of the two indices may help distinguish people with overt metabolic derangement from those whose residual risk remains elevated despite ostensibly intact metabolism. Effect magnitudes in the present cohort also eclipsed those previously reported for stroke [46] and cardiovascular disease [47], raising the possibility that CircS is especially responsive to insulin-resistance-driven metabolic perturbation; in support of this, experimental work demonstrates that insulin directly modulates clock genes such as BMAL1 and PER2 in peripheral tissues [48] and that disrupted insulin signalling can destabilise circadian oscillations [49]. The inverse eGDR–CircS gradient (Q4 vs Q1 RR 0.28) is consistent with a protective role for preserved insulin sensitivity. Originally devised to quantify glucose disposal in type 1 diabetes [50], eGDR has since been tied to cardiovascular events [51], cardiovascular risk across distinct metabolic and circadian milieus [52], and all-cause mortality [53]. METS-IR, a composite of fasting glucose, triglycerides, BMI, and HDL-C [18], supplied the second-largest quartile-based effect size (RR 3.38). More broadly, analogous composite metabolic scores track visceral adiposity and cardiometabolic status [54], and atherogenic risk profiles remain tightly coupled with cardiovascular disease [55].

Both atherogenic-lipid indices—AIP and the CHG index—were likewise associated with incident CircS. AIP has been connected to recurrent ischaemic stroke [56] and to obesity-related dyslipidaemia [57]; as a metric it captures the equilibrium between pro- and anti-atherogenic lipoproteins, with higher values signalling a tilt toward small dense LDL particles [20]. At a broader level, circadian governance of metabolic fitness and disease offers a plausible framework for the lipid-related signals seen here [58]. Lipid-metabolic pathways are themselves under clock control and are modulated by immunometabolic crosstalk [59, 60], so an elevated AIP may mark a lipid phenotype that simultaneously reflects and amplifies circadian dysregulation. The CHG index, integrating total cholesterol, fasting glucose, and HDL-C [21], produced the greatest incremental AUC (0.737), indicating that a composite synthesising glucometabolic and lipid information captures risk dimensions poorly resolved by single-pathway markers. In contrast, the lipid-inflammatory indices RCII and hs-CRP/HDL-C yielded weaker but still significant associations. The resulting gradient implies that insulin resistance and atherogenic dyslipidaemia may occupy a more direct causal position in CircS development than systemic inflammation on its own.

Findings from the bidirectional mediation analysis reinforce that interpretation. Along the forward direction, hs-CRP did not mediate any individual index–CircS relationship, implying that inflammation is unlikely to be the principal intermediate step. The null forward pathway may partly reflect hs-CRP's status as a relatively downstream and non-specific marker; the metabolic indices may instead act through several inflammatory mediators—IL-6, TNF-α, and adipokines among them—that hs-CRP alone fails to capture, while the short half-life of hs-CRP may also be inadequate to represent a chronic 4-year inflammatory burden. Along the reverse direction, systemic inflammation proxied by hs-CRP can perturb hepatic lipid handling by enhancing de novo lipogenesis and dampening fatty acid oxidation via NF-κB and STAT3 signalling, generating hypertriglyceridaemia and reduced HDL-C, which would mechanically inflate the CHG index and METS-IR. The resulting atherogenic–dysmetabolic profile could accelerate CircS onset by aggravating insulin resistance, central adiposity, and blood-pressure dysregulation, which fits models of metabolic inflammation in which immune dysregulation perturbs lipid and glucose homeostasis [59, 60]. Recently delineated molecular circuits—including the voltage-gated potassium channel Kv1.3 [61] and the Adss1–HDAC3 axis [62]—may bridge metabolic perturbation with circadian clock function through effects on membrane excitability and epigenetic reprogramming of adipocyte energy metabolism. Biological-age acceleration added a further mechanistic layer: the fraction of the eGDR–CircS association carried by biological age reached 42.8%, whereas the corresponding proportions for TyG-BMI and CTI were far smaller (3.9% and 6.6%, respectively). This pattern dovetails with the broader literature linking accelerated biological ageing to chronic disease and early mortality [63, 64]. The remaining indices may operate through ageing-independent routes, including ectopic fat deposition [65] and hepatic lipid metabolism [66]. Circadian regulation itself also loses stability with age through attenuated clock amplitude and increasing sleep fragmentation [67, 68], so preserved insulin sensitivity may attenuate age-related circadian decline [69].

These findings sit at the edge of a still-expanding CircS literature. Most published work has treated CircS as an upstream exposure predicting a wide range of downstream outcomes—cardio–kidney events [70], all-cause mortality [71, 72], cognitive impairment [11, 73], cardiometabolic multimorbidity [13], chronic kidney disease [14], and cancer [74]. Here, by contrast, CircS served as the endpoint, and we searched for upstream metabolic predictors of its onset. A cross-sectional analysis by Zhang and colleagues separately reported eGDR as a predictor of CircS [42]; the fact that the same association emerges across different study designs lends cumulative support to the clinical relevance of eGDR. Additional alignment comes from reports that connect eGDR with cardiovascular risk under varying CircS states [52] and that cast the TyG index as a mediator between CircS and cancer [74]. We acknowledge that several exposure indices share components with the metabolic criteria of CircS (notably TyG-BMI and METS-IR); the resulting tautology risk, and the corresponding role of IPTW and sensitivity analyses in mitigating it, is addressed in the Limitations subsection below. Notably, eGDR—whose only overlap with the CircS definition is waist circumference—continued to show significant associations with incident CircS across every analytical framework, which reinforces the clinical plausibility of the observed gradient.

The machine-learning findings carry practical weight. On the test set, logistic regression edged past all nine more intricate algorithms (AUC 0.746 versus 0.737 for the nearest competitor), echoing evidence that machine learning frequently fails to outperform logistic regression for clinical prediction [75] and aligning with guidance that privileges out-of-sample behaviour over in-sample fit [76–78]. The moderate sample size and modest predictor dimensionality of the current dataset probably favour parsimonious models, which is consistent with that pattern. Independently, SHAP attribution flagged eGDR as the dominant contributor (mean absolute SHAP value 0.487), whereas the LASSO coefficient path allotted the largest weight to METS-IR—a divergence most plausibly ascribed to the different ways the two methods cope with correlated predictors [29]. The repeated emergence of eGDR across regression, SHAP, and machine-learning views speaks to its potential clinical usefulness. Decision-curve analysis indicated that logistic regression delivered the greatest net benefit at threshold probabilities between 10 and 40%, and calibration was acceptable overall, albeit with mild overestimation at the upper tail of predicted risk [79].

Our study has several strengths. It draws on a population-representative cohort, uses a prospective design, performs a comprehensive evaluation of eight metabolic composites across four pathophysiological domains, and layers several complementary analytic methods—modified Poisson regression, Cox proportional hazards models, restricted cubic splines, bidirectional mediation, IPTW, and machine learning with SHAP interpretation. Convergence of findings across these methods reinforces confidence in the conclusions. Several limitations also warrant acknowledgement. First, laboratory variables carried approximately 54% missingness as a consequence of the CHARLS blood-biomarker sub-study architecture (Table S2), and physical-activity measurements were available for only 42% of the analytic sample; because this missingness reflects structural sub-study sampling rather than item-level dropout, we relied on complete-case estimates, and multiple-imputation sensitivity analyses returned concordant results (Table S2). Second, several CircS components (diabetes, hypertension, dyslipidaemia) were ascertained through self-reported physician diagnosis, which can seed non-differential misclassification toward the null—or, in the case of dyslipidaemia, differential misclassification biased toward health-seeking individuals—so lipid-based index estimates warrant cautious interpretation. Third, a single waist-circumference cutoff (≥ 85 cm) was retained to preserve comparability with the original CircS framework [5] and prior CHARLS-based analyses, even though sex-specific thresholds are available in Chinese clinical guidelines. Fourth, partial structural overlap between the formulae of several exposure indices and the metabolic criteria of CircS can inflate effect estimates by construction; although adjustment for shared clinical states (BMI, hypertension, diabetes) and IPTW reweighting were implemented, residual mathematical coupling cannot be wholly eliminated, and the absolute effect sizes for TyG-BMI and METS-IR should be read alongside the cross-index ranking reported in Table S3. Fifth, every model was validated internally within CHARLS through a 70/30 split, and no independent external validation was attempted; in line with the Transparent Reporting of a multivariable prediction model for Individual Prognosis Or Diagnosis extended to Artificial Intelligence (TRIPOD + AI) framework [77] and recent risk-of-bias guidance [78], the discrimination metrics reported here should therefore be interpreted as upper-bound estimates, and external validation in independent Chinese cohorts (e.g. CHNS, CLHLS, or Dongfeng-Tongji) or in ethnically diverse populations will be needed to gauge transportability, particularly because downstream manifestations of metabolic dysregulation such as vascular calcification may behave differently across populations with differing burdens of chronic kidney disease and genetic susceptibility [80].

## Conclusions

In a nationally representative cohort of 4,325 middle-aged and older Chinese adults, eight non-traditional metabolic indices spanning insulin resistance, atherogenic lipid, and inflammatory pathways independently predicted incident circadian syndrome over 4 years of follow-up. TyG-BMI and eGDR showed the largest effect sizes, and the CHG index provided the greatest incremental predictive value. Biological-age acceleration partially mediated these associations, particularly for eGDR. Among ten machine-learning algorithms, logistic regression achieved the best test-set performance, and SHAP analysis confirmed eGDR as the most important predictor. These findings support the use of metabolic indices, particularly eGDR and the CHG index, in CircS risk assessment to facilitate early identification of individuals at high risk.

## Supplementary Information

Supplementary Material 1.

## Data Availability

The datasets used in this study are publicly available from the CHARLS repository at https://charls.pku.edu.cn/. Additional analysis code is available from the corresponding author on reasonable request.

## Abbreviations

AIP: Atherogenic index of plasma
CHG: Cholesterol-HDL-glucose index
CHARLS: China Health and Retirement Longitudinal Study
CircS: Circadian syndrome
CTI: C-reactive protein triglyceride glucose index
eGDR: Estimated glucose disposal rate
HbA1c: Glycated hemoglobin
HDL-C: High-density lipoprotein cholesterol
hs-CRP: High-sensitivity C-reactive protein
IDI: Integrated discrimination improvement
IPTW: Inverse probability of treatment weighting
METS-IR: Metabolic score for insulin resistance
NRI: Net reclassification improvement
RCII: Remnant cholesterol inflammatory index
RCS: Restricted cubic spline
RR: Risk ratio
SHAP: SHapley Additive exPlanations
TyG-BMI: Triglyceride-glucose body mass index
