# Supplementary material for: Non-traditional metabolic indices predict incident circadian syndrome in middle-aged and older Chinese adults: a nationwide prospective cohort study and machine learning analysis
Source: Lipids Health Dis. 2026 May 13;25:167. doi: 10.1186/s12944-026-02972-9 (PMC13339493; doi:10.1186/s12944-026-02972-9)
Supplement: Supplementary file 1 — Supplementary Material 1. [file 12944_2026_2972_MOESM1_ESM.zip › Table_S01.docx]

Table S1. Sequential exclusion criteria and sample derivation

| **Step** | **Reason** | **N.excluded** | **N.remaining** | **Cohort** |
| --- | --- | --- | --- | --- |
| S1 | Age < 45 years | 8,655 | 16,931 | CHARLS |
| S2 | Baseline cancer or missing cancer status | 158 | 16,773 | CHARLS |
| S3 | Prevalent CircS at baseline or missing | 10,129 | 6,644 | CHARLS |
| S4 | Missing follow-up CircS status (W3) | 2,319 | 4,325 | CHARLS |
| S5 | Final analytic sample | 0 | 4,325 | CHARLS |
| *CHARLS, China Health and Retirement Longitudinal Study; CircS, circadian syndrome.* | | | | |
