# Supplementary material for: Non-traditional metabolic indices predict incident circadian syndrome in middle-aged and older Chinese adults: a nationwide prospective cohort study and machine learning analysis
Source: Lipids Health Dis. 2026 May 13;25:167. doi: 10.1186/s12944-026-02972-9 (PMC13339493; doi:10.1186/s12944-026-02972-9)
Supplement: Supplementary file 1 — Supplementary Material 1. [file 12944_2026_2972_MOESM1_ESM.zip › Table_S02.docx]

**Table S2. Missing data patterns for key variables in the full CHARLS cohort (N = 25,586)**

| **Variable** | **N total** | **N available** | **N missing** | **Missing (%)** |
| --- | --- | --- | --- | --- |
| Triglycerides | 25,586 | 11,656 | 13,930 | 54.4 |
| Total cholesterol | 25,586 | 11,655 | 13,931 | 54.4 |
| HDL-C | 25,586 | 11,663 | 13,923 | 54.4 |
| LDL-C | 25,586 | 11,642 | 13,944 | 54.5 |
| hs-CRP | 25,586 | 11,664 | 13,922 | 54.4 |
| Fasting plasma glucose | 25,586 | 11,636 | 13,950 | 54.5 |
| HbA1c | 25,586 | 11,706 | 13,880 | 54.2 |
| BMI | 25,586 | 13,631 | 11,955 | 46.7 |
| Waist circumference | 25,586 | 13,769 | 11,817 | 46.2 |
| Age | 25,586 | 17,533 | 8,053 | 31.5 |
| Sex | 25,586 | 25,578 | 8 | 0.0 |
| Residence | 25,586 | 17,708 | 7,878 | 30.8 |
| Marital status | 25,586 | 17,675 | 7,911 | 30.9 |
| Education level | 25,586 | 25,542 | 44 | 0.2 |
| Smoking status | 25,586 | 17,559 | 8,027 | 31.4 |
| Alcohol consumption | 25,586 | 16,354 | 9,232 | 36.1 |
| Hypertension | 25,586 | 17,470 | 8,116 | 31.7 |
| Diabetes | 25,586 | 17,408 | 8,178 | 32.0 |
| Lipid-lowering medication | 25,586 | 17,201 | 8,385 | 32.8 |
| *Missing rates are reported for the total CHARLS Wave 1 sample before application of exclusion criteria.* | | | | |
