# Supplementary material for: Non-traditional metabolic indices predict incident circadian syndrome in middle-aged and older Chinese adults: a nationwide prospective cohort study and machine learning analysis
Source: Lipids Health Dis. 2026 May 13;25:167. doi: 10.1186/s12944-026-02972-9 (PMC13339493; doi:10.1186/s12944-026-02972-9)
Supplement: Supplementary file 1 — Supplementary Material 1. [file 12944_2026_2972_MOESM1_ESM.zip › Table_S03.docx]

**Table S3. Overlap documentation between metabolic index components and CircS criteria**

| **Index** | **Index label** | **Overlapping CircS components** | **Overlap degree** |
| --- | --- | --- | --- |
| AIP | AIP | TG, HDL-C | Moderate |
| CHG Index | CHG Index | FPG, HDL-C | Moderate |
| RCII | RCII | HDL-C (via RC) | Low |
| hs-CRP/HDL-C | hs-CRP/HDL-C | HDL-C | Low |
| CTI | CTI | TG, FPG, HDL-C | High |
| TyG-BMI | TyG-BMI | TG, FPG | Moderate |
| eGDR | eGDR | WC (HbA1c is proxy, not FPG directly) | Low |
| METS-IR | METS-IR | FPG, TG, HDL-C | High |
| *CircS, circadian syndrome.* | | | |
