# Supplementary material for: Non-traditional metabolic indices predict incident circadian syndrome in middle-aged and older Chinese adults: a nationwide prospective cohort study and machine learning analysis
Source: Lipids Health Dis. 2026 May 13;25:167. doi: 10.1186/s12944-026-02972-9 (PMC13339493; doi:10.1186/s12944-026-02972-9)
Supplement: Supplementary file 1 — Supplementary Material 1. [file 12944_2026_2972_MOESM1_ESM.zip › Table_S04.docx]

**Table S4. P-values for overall association and non-linearity from restricted cubic spline analyses**

| **Index** | **N** | **Events** | **P overall** | **P nonlinear** | **Index label** |
| --- | --- | --- | --- | --- | --- |
| AIP | 3,356 | 725 | <0.001 | 0.016 | AIP |
| CHG Index | 3,004 | 648 | <0.001 | 0.012 | CHG Index |
| RCII | 3,354 | 725 | <0.001 | 0.001 | RCII |
| hs-CRP/HDL-C | 3,358 | 725 | 0.001 | 0.002 | hs-CRP/HDL-C |
| CTI | 3,353 | 725 | <0.001 | 0.852 | CTI |
| TyG-BMI | 3,353 | 725 | <0.001 | <0.001 | TyG-BMI |
| eGDR | 3,345 | 729 | <0.001 | <0.001 | eGDR |
| METS-IR | 3,353 | 725 | <0.001 | <0.001 | METS-IR |
| *P overall tests overall association; P nonlinearity tests departure from linearity.* | | | | | |
