# Supplementary material for: Non-traditional metabolic indices predict incident circadian syndrome in middle-aged and older Chinese adults: a nationwide prospective cohort study and machine learning analysis
Source: Lipids Health Dis. 2026 May 13;25:167. doi: 10.1186/s12944-026-02972-9 (PMC13339493; doi:10.1186/s12944-026-02972-9)
Supplement: Supplementary file 1 — Supplementary Material 1. [file 12944_2026_2972_MOESM1_ESM.zip › Table_S05.docx]

**Table S5. Restricted cubic spline analysis: risk ratios at selected values**

| **Index** | **Index value** | **RR** | **Lower CI** | **Upper CI** |
| --- | --- | --- | --- | --- |
| AIP | -0.285 | 0.377 | 0.203 | 0.702 |
| AIP | -0.279 | 0.382 | 0.209 | 0.697 |
| AIP | -0.273 | 0.386 | 0.215 | 0.693 |
| AIP | -0.267 | 0.390 | 0.221 | 0.689 |
| AIP | -0.261 | 0.395 | 0.228 | 0.685 |
| AIP | -0.254 | 0.399 | 0.234 | 0.681 |
| AIP | -0.248 | 0.404 | 0.241 | 0.677 |
| AIP | -0.242 | 0.408 | 0.247 | 0.674 |
| AIP | -0.236 | 0.413 | 0.254 | 0.671 |
| AIP | -0.230 | 0.418 | 0.261 | 0.667 |
| AIP | -0.224 | 0.422 | 0.268 | 0.664 |
| AIP | -0.218 | 0.427 | 0.276 | 0.662 |
| AIP | -0.212 | 0.432 | 0.283 | 0.659 |
| AIP | -0.205 | 0.437 | 0.290 | 0.657 |
| AIP | -0.199 | 0.442 | 0.298 | 0.655 |
| AIP | -0.193 | 0.447 | 0.305 | 0.654 |
| AIP | -0.187 | 0.452 | 0.313 | 0.652 |
| AIP | -0.181 | 0.457 | 0.321 | 0.651 |
| AIP | -0.175 | 0.462 | 0.328 | 0.651 |
| AIP | -0.169 | 0.468 | 0.336 | 0.651 |
| AIP | -0.162 | 0.473 | 0.344 | 0.651 |
| AIP | -0.156 | 0.478 | 0.351 | 0.651 |
| AIP | -0.150 | 0.484 | 0.359 | 0.652 |
| AIP | -0.144 | 0.490 | 0.367 | 0.653 |
| AIP | -0.138 | 0.495 | 0.374 | 0.655 |
| AIP | -0.132 | 0.501 | 0.382 | 0.658 |
| AIP | -0.126 | 0.507 | 0.389 | 0.660 |
| AIP | -0.120 | 0.513 | 0.396 | 0.664 |
| AIP | -0.113 | 0.519 | 0.403 | 0.667 |
| AIP | -0.107 | 0.525 | 0.410 | 0.672 |
| AIP | -0.101 | 0.531 | 0.417 | 0.676 |
| AIP | -0.095 | 0.537 | 0.424 | 0.682 |
| AIP | -0.089 | 0.544 | 0.430 | 0.687 |
| AIP | -0.083 | 0.550 | 0.437 | 0.693 |
| AIP | -0.077 | 0.557 | 0.443 | 0.700 |
| AIP | -0.071 | 0.564 | 0.449 | 0.707 |
| AIP | -0.064 | 0.571 | 0.456 | 0.715 |
| AIP | -0.058 | 0.578 | 0.462 | 0.723 |
| AIP | -0.052 | 0.585 | 0.468 | 0.731 |
| AIP | -0.046 | 0.592 | 0.474 | 0.739 |
| AIP | -0.040 | 0.599 | 0.480 | 0.748 |
| AIP | -0.034 | 0.606 | 0.486 | 0.757 |
| AIP | -0.028 | 0.614 | 0.492 | 0.766 |
| AIP | -0.021 | 0.622 | 0.498 | 0.776 |
| AIP | -0.015 | 0.630 | 0.505 | 0.785 |
| AIP | -0.009 | 0.637 | 0.511 | 0.795 |
| AIP | -0.003 | 0.646 | 0.518 | 0.804 |
| AIP | 0.003 | 0.654 | 0.525 | 0.814 |
| AIP | 0.009 | 0.662 | 0.532 | 0.824 |
| AIP | 0.015 | 0.671 | 0.540 | 0.833 |
| AIP | 0.021 | 0.679 | 0.547 | 0.843 |
| AIP | 0.028 | 0.688 | 0.555 | 0.853 |
| AIP | 0.034 | 0.697 | 0.564 | 0.862 |
| AIP | 0.040 | 0.706 | 0.573 | 0.871 |
| AIP | 0.046 | 0.716 | 0.582 | 0.881 |
| AIP | 0.052 | 0.725 | 0.591 | 0.890 |
| AIP | 0.058 | 0.735 | 0.601 | 0.899 |
| AIP | 0.064 | 0.745 | 0.611 | 0.908 |
| AIP | 0.070 | 0.755 | 0.622 | 0.917 |
| AIP | 0.077 | 0.765 | 0.632 | 0.926 |
| AIP | 0.083 | 0.775 | 0.643 | 0.935 |
| AIP | 0.089 | 0.786 | 0.654 | 0.944 |
| AIP | 0.095 | 0.797 | 0.666 | 0.953 |
| AIP | 0.101 | 0.807 | 0.677 | 0.963 |
| AIP | 0.107 | 0.818 | 0.688 | 0.973 |
| AIP | 0.113 | 0.829 | 0.699 | 0.983 |
| AIP | 0.120 | 0.840 | 0.711 | 0.994 |
| AIP | 0.126 | 0.851 | 0.722 | 1.005 |
| AIP | 0.132 | 0.863 | 0.732 | 1.016 |
| AIP | 0.138 | 0.874 | 0.743 | 1.028 |
| AIP | 0.144 | 0.886 | 0.753 | 1.041 |
| AIP | 0.150 | 0.897 | 0.763 | 1.054 |
| AIP | 0.156 | 0.909 | 0.773 | 1.068 |
| AIP | 0.162 | 0.920 | 0.783 | 1.082 |
| AIP | 0.169 | 0.932 | 0.792 | 1.096 |
| AIP | 0.175 | 0.943 | 0.801 | 1.110 |
| AIP | 0.181 | 0.955 | 0.810 | 1.125 |
| AIP | 0.187 | 0.966 | 0.819 | 1.140 |
| AIP | 0.193 | 0.978 | 0.828 | 1.155 |
| AIP | 0.199 | 0.989 | 0.837 | 1.170 |
| AIP | 0.205 | 1.001 | 0.845 | 1.184 |
| AIP | 0.211 | 1.012 | 0.854 | 1.199 |
| AIP | 0.218 | 1.023 | 0.863 | 1.213 |
| AIP | 0.224 | 1.034 | 0.872 | 1.226 |
| AIP | 0.230 | 1.045 | 0.881 | 1.239 |
| AIP | 0.236 | 1.056 | 0.891 | 1.252 |
| AIP | 0.242 | 1.067 | 0.900 | 1.264 |
| AIP | 0.248 | 1.077 | 0.910 | 1.276 |
| AIP | 0.254 | 1.088 | 0.919 | 1.288 |
| AIP | 0.261 | 1.098 | 0.929 | 1.299 |
| AIP | 0.267 | 1.109 | 0.939 | 1.310 |
| AIP | 0.273 | 1.119 | 0.949 | 1.320 |
| AIP | 0.279 | 1.129 | 0.959 | 1.330 |
| AIP | 0.285 | 1.139 | 0.969 | 1.340 |
| AIP | 0.291 | 1.149 | 0.979 | 1.350 |
| AIP | 0.297 | 1.159 | 0.988 | 1.359 |
| AIP | 0.303 | 1.169 | 0.998 | 1.369 |
| AIP | 0.310 | 1.179 | 1.008 | 1.378 |
| AIP | 0.316 | 1.188 | 1.018 | 1.387 |
| AIP | 0.322 | 1.198 | 1.027 | 1.396 |
| AIP | 0.328 | 1.207 | 1.037 | 1.405 |
| AIP | 0.334 | 1.216 | 1.046 | 1.415 |
| AIP | 0.340 | 1.226 | 1.055 | 1.424 |
| AIP | 0.346 | 1.235 | 1.064 | 1.434 |
| AIP | 0.352 | 1.244 | 1.072 | 1.443 |
| AIP | 0.359 | 1.253 | 1.080 | 1.453 |
| AIP | 0.365 | 1.262 | 1.088 | 1.463 |
| AIP | 0.371 | 1.271 | 1.096 | 1.474 |
| AIP | 0.377 | 1.280 | 1.103 | 1.484 |
| AIP | 0.383 | 1.288 | 1.110 | 1.495 |
| AIP | 0.389 | 1.297 | 1.117 | 1.506 |
| AIP | 0.395 | 1.306 | 1.124 | 1.516 |
| AIP | 0.402 | 1.314 | 1.131 | 1.528 |
| AIP | 0.408 | 1.323 | 1.137 | 1.539 |
| AIP | 0.414 | 1.331 | 1.143 | 1.550 |
| AIP | 0.420 | 1.339 | 1.149 | 1.561 |
| AIP | 0.426 | 1.348 | 1.155 | 1.573 |
| AIP | 0.432 | 1.356 | 1.161 | 1.584 |
| AIP | 0.438 | 1.364 | 1.166 | 1.595 |
| AIP | 0.444 | 1.372 | 1.172 | 1.607 |
| AIP | 0.451 | 1.380 | 1.177 | 1.618 |
| AIP | 0.457 | 1.388 | 1.182 | 1.630 |
| AIP | 0.463 | 1.396 | 1.188 | 1.641 |
| AIP | 0.469 | 1.404 | 1.193 | 1.652 |
| AIP | 0.475 | 1.412 | 1.198 | 1.663 |
| AIP | 0.481 | 1.419 | 1.203 | 1.675 |
| AIP | 0.487 | 1.427 | 1.208 | 1.686 |
| AIP | 0.493 | 1.434 | 1.212 | 1.697 |
| AIP | 0.500 | 1.442 | 1.217 | 1.707 |
| AIP | 0.506 | 1.449 | 1.222 | 1.718 |
| AIP | 0.512 | 1.456 | 1.227 | 1.729 |
| AIP | 0.518 | 1.463 | 1.231 | 1.739 |
| AIP | 0.524 | 1.470 | 1.236 | 1.750 |
| AIP | 0.530 | 1.477 | 1.240 | 1.760 |
| AIP | 0.536 | 1.484 | 1.245 | 1.770 |
| AIP | 0.543 | 1.491 | 1.249 | 1.780 |
| AIP | 0.549 | 1.498 | 1.254 | 1.790 |
| AIP | 0.555 | 1.505 | 1.258 | 1.799 |
| AIP | 0.561 | 1.511 | 1.262 | 1.809 |
| AIP | 0.567 | 1.518 | 1.266 | 1.819 |
| AIP | 0.573 | 1.524 | 1.271 | 1.828 |
| AIP | 0.579 | 1.530 | 1.275 | 1.837 |
| AIP | 0.585 | 1.536 | 1.279 | 1.846 |
| AIP | 0.592 | 1.543 | 1.283 | 1.855 |
| AIP | 0.598 | 1.549 | 1.287 | 1.864 |
| AIP | 0.604 | 1.555 | 1.291 | 1.872 |
| AIP | 0.610 | 1.561 | 1.295 | 1.881 |
| AIP | 0.616 | 1.566 | 1.298 | 1.889 |
| AIP | 0.622 | 1.572 | 1.302 | 1.898 |
| AIP | 0.628 | 1.578 | 1.306 | 1.906 |
| AIP | 0.635 | 1.583 | 1.309 | 1.914 |
| AIP | 0.641 | 1.589 | 1.313 | 1.922 |
| AIP | 0.647 | 1.594 | 1.316 | 1.930 |
| AIP | 0.653 | 1.599 | 1.320 | 1.938 |
| AIP | 0.659 | 1.604 | 1.323 | 1.946 |
| AIP | 0.665 | 1.609 | 1.326 | 1.953 |
| AIP | 0.671 | 1.614 | 1.329 | 1.961 |
| AIP | 0.677 | 1.619 | 1.332 | 1.969 |
| AIP | 0.684 | 1.624 | 1.334 | 1.976 |
| AIP | 0.690 | 1.629 | 1.337 | 1.984 |
| AIP | 0.696 | 1.633 | 1.340 | 1.991 |
| AIP | 0.702 | 1.638 | 1.342 | 1.999 |
| AIP | 0.708 | 1.642 | 1.344 | 2.007 |
| AIP | 0.714 | 1.647 | 1.346 | 2.014 |
| AIP | 0.720 | 1.651 | 1.348 | 2.022 |
| AIP | 0.726 | 1.655 | 1.350 | 2.029 |
| AIP | 0.733 | 1.659 | 1.351 | 2.037 |
| AIP | 0.739 | 1.663 | 1.353 | 2.045 |
| AIP | 0.745 | 1.667 | 1.354 | 2.053 |
| AIP | 0.751 | 1.671 | 1.355 | 2.060 |
| AIP | 0.757 | 1.675 | 1.356 | 2.068 |
| AIP | 0.763 | 1.678 | 1.356 | 2.076 |
| AIP | 0.769 | 1.682 | 1.357 | 2.085 |
| AIP | 0.776 | 1.685 | 1.357 | 2.093 |
| AIP | 0.782 | 1.689 | 1.357 | 2.101 |
| AIP | 0.788 | 1.692 | 1.357 | 2.110 |
| AIP | 0.794 | 1.695 | 1.356 | 2.119 |
| AIP | 0.800 | 1.698 | 1.356 | 2.127 |
| AIP | 0.806 | 1.701 | 1.355 | 2.136 |
| AIP | 0.812 | 1.704 | 1.354 | 2.146 |
| AIP | 0.818 | 1.707 | 1.353 | 2.155 |
| AIP | 0.825 | 1.710 | 1.351 | 2.164 |
| AIP | 0.831 | 1.713 | 1.349 | 2.174 |
| AIP | 0.837 | 1.715 | 1.347 | 2.184 |
| AIP | 0.843 | 1.718 | 1.345 | 2.194 |
| AIP | 0.849 | 1.721 | 1.343 | 2.204 |
| AIP | 0.855 | 1.723 | 1.340 | 2.215 |
| AIP | 0.861 | 1.725 | 1.337 | 2.226 |
| AIP | 0.867 | 1.728 | 1.334 | 2.237 |
| AIP | 0.874 | 1.730 | 1.331 | 2.248 |
| AIP | 0.880 | 1.732 | 1.328 | 2.259 |
| AIP | 0.886 | 1.734 | 1.324 | 2.271 |
| AIP | 0.892 | 1.736 | 1.320 | 2.283 |
| AIP | 0.898 | 1.738 | 1.316 | 2.295 |
| AIP | 0.904 | 1.740 | 1.312 | 2.307 |
| AIP | 0.910 | 1.741 | 1.307 | 2.320 |
| AIP | 0.917 | 1.743 | 1.302 | 2.333 |
| AIP | 0.923 | 1.745 | 1.298 | 2.346 |
| AIP | 0.929 | 1.746 | 1.292 | 2.359 |
| AIP | 0.935 | 1.748 | 1.287 | 2.373 |
| CHG Index | 4.518 | 0.518 | 0.275 | 0.978 |
| CHG Index | 4.525 | 0.518 | 0.279 | 0.960 |
| CHG Index | 4.533 | 0.517 | 0.284 | 0.943 |
| CHG Index | 4.540 | 0.517 | 0.288 | 0.926 |
| CHG Index | 4.548 | 0.516 | 0.293 | 0.910 |
| CHG Index | 4.555 | 0.516 | 0.298 | 0.894 |
| CHG Index | 4.562 | 0.515 | 0.302 | 0.878 |
| CHG Index | 4.570 | 0.515 | 0.307 | 0.864 |
| CHG Index | 4.577 | 0.515 | 0.312 | 0.849 |
| CHG Index | 4.585 | 0.514 | 0.317 | 0.835 |
| CHG Index | 4.592 | 0.514 | 0.322 | 0.822 |
| CHG Index | 4.599 | 0.514 | 0.326 | 0.809 |
| CHG Index | 4.607 | 0.514 | 0.331 | 0.797 |
| CHG Index | 4.614 | 0.514 | 0.336 | 0.785 |
| CHG Index | 4.622 | 0.514 | 0.341 | 0.774 |
| CHG Index | 4.629 | 0.514 | 0.345 | 0.764 |
| CHG Index | 4.636 | 0.514 | 0.350 | 0.754 |
| CHG Index | 4.644 | 0.514 | 0.355 | 0.744 |
| CHG Index | 4.651 | 0.514 | 0.359 | 0.736 |
| CHG Index | 4.659 | 0.514 | 0.364 | 0.727 |
| CHG Index | 4.666 | 0.515 | 0.368 | 0.720 |
| CHG Index | 4.673 | 0.515 | 0.372 | 0.713 |
| CHG Index | 4.681 | 0.516 | 0.377 | 0.707 |
| CHG Index | 4.688 | 0.517 | 0.381 | 0.701 |
| CHG Index | 4.696 | 0.517 | 0.385 | 0.696 |
| CHG Index | 4.703 | 0.518 | 0.389 | 0.692 |
| CHG Index | 4.710 | 0.519 | 0.392 | 0.688 |
| CHG Index | 4.718 | 0.521 | 0.396 | 0.685 |
| CHG Index | 4.725 | 0.522 | 0.399 | 0.683 |
| CHG Index | 4.732 | 0.524 | 0.403 | 0.681 |
| CHG Index | 4.740 | 0.525 | 0.406 | 0.680 |
| CHG Index | 4.747 | 0.527 | 0.409 | 0.679 |
| CHG Index | 4.755 | 0.529 | 0.412 | 0.679 |
| CHG Index | 4.762 | 0.531 | 0.415 | 0.680 |
| CHG Index | 4.769 | 0.533 | 0.417 | 0.681 |
| CHG Index | 4.777 | 0.536 | 0.420 | 0.683 |
| CHG Index | 4.784 | 0.538 | 0.423 | 0.685 |
| CHG Index | 4.792 | 0.541 | 0.426 | 0.688 |
| CHG Index | 4.799 | 0.544 | 0.428 | 0.691 |
| CHG Index | 4.806 | 0.547 | 0.431 | 0.695 |
| CHG Index | 4.814 | 0.551 | 0.434 | 0.699 |
| CHG Index | 4.821 | 0.555 | 0.437 | 0.704 |
| CHG Index | 4.829 | 0.559 | 0.440 | 0.709 |
| CHG Index | 4.836 | 0.563 | 0.444 | 0.714 |
| CHG Index | 4.843 | 0.567 | 0.447 | 0.720 |
| CHG Index | 4.851 | 0.572 | 0.451 | 0.725 |
| CHG Index | 4.858 | 0.577 | 0.455 | 0.732 |
| CHG Index | 4.866 | 0.582 | 0.459 | 0.738 |
| CHG Index | 4.873 | 0.588 | 0.464 | 0.745 |
| CHG Index | 4.880 | 0.594 | 0.469 | 0.752 |
| CHG Index | 4.888 | 0.600 | 0.474 | 0.759 |
| CHG Index | 4.895 | 0.607 | 0.480 | 0.766 |
| CHG Index | 4.903 | 0.614 | 0.487 | 0.774 |
| CHG Index | 4.910 | 0.621 | 0.493 | 0.782 |
| CHG Index | 4.917 | 0.629 | 0.501 | 0.790 |
| CHG Index | 4.925 | 0.637 | 0.508 | 0.798 |
| CHG Index | 4.932 | 0.645 | 0.517 | 0.806 |
| CHG Index | 4.940 | 0.655 | 0.526 | 0.815 |
| CHG Index | 4.947 | 0.664 | 0.535 | 0.824 |
| CHG Index | 4.954 | 0.674 | 0.545 | 0.833 |
| CHG Index | 4.962 | 0.684 | 0.556 | 0.843 |
| CHG Index | 4.969 | 0.695 | 0.567 | 0.852 |
| CHG Index | 4.977 | 0.707 | 0.579 | 0.863 |
| CHG Index | 4.984 | 0.718 | 0.591 | 0.873 |
| CHG Index | 4.991 | 0.730 | 0.603 | 0.884 |
| CHG Index | 4.999 | 0.743 | 0.616 | 0.896 |
| CHG Index | 5.006 | 0.756 | 0.629 | 0.907 |
| CHG Index | 5.014 | 0.769 | 0.643 | 0.920 |
| CHG Index | 5.021 | 0.782 | 0.656 | 0.933 |
| CHG Index | 5.028 | 0.796 | 0.670 | 0.947 |
| CHG Index | 5.036 | 0.810 | 0.683 | 0.961 |
| CHG Index | 5.043 | 0.825 | 0.697 | 0.976 |
| CHG Index | 5.051 | 0.839 | 0.710 | 0.992 |
| CHG Index | 5.058 | 0.854 | 0.723 | 1.008 |
| CHG Index | 5.065 | 0.869 | 0.736 | 1.025 |
| CHG Index | 5.073 | 0.884 | 0.749 | 1.042 |
| CHG Index | 5.080 | 0.899 | 0.762 | 1.060 |
| CHG Index | 5.088 | 0.914 | 0.775 | 1.079 |
| CHG Index | 5.095 | 0.929 | 0.787 | 1.098 |
| CHG Index | 5.102 | 0.944 | 0.799 | 1.116 |
| CHG Index | 5.110 | 0.959 | 0.810 | 1.136 |
| CHG Index | 5.117 | 0.974 | 0.822 | 1.154 |
| CHG Index | 5.125 | 0.989 | 0.833 | 1.173 |
| CHG Index | 5.132 | 1.003 | 0.845 | 1.192 |
| CHG Index | 5.139 | 1.017 | 0.856 | 1.209 |
| CHG Index | 5.147 | 1.031 | 0.867 | 1.227 |
| CHG Index | 5.154 | 1.045 | 0.878 | 1.243 |
| CHG Index | 5.161 | 1.058 | 0.888 | 1.259 |
| CHG Index | 5.169 | 1.071 | 0.899 | 1.275 |
| CHG Index | 5.176 | 1.083 | 0.910 | 1.290 |
| CHG Index | 5.184 | 1.096 | 0.920 | 1.304 |
| CHG Index | 5.191 | 1.108 | 0.931 | 1.318 |
| CHG Index | 5.198 | 1.119 | 0.942 | 1.331 |
| CHG Index | 5.206 | 1.131 | 0.952 | 1.343 |
| CHG Index | 5.213 | 1.142 | 0.962 | 1.355 |
| CHG Index | 5.221 | 1.153 | 0.973 | 1.367 |
| CHG Index | 5.228 | 1.164 | 0.983 | 1.378 |
| CHG Index | 5.235 | 1.174 | 0.993 | 1.388 |
| CHG Index | 5.243 | 1.184 | 1.003 | 1.399 |
| CHG Index | 5.250 | 1.194 | 1.012 | 1.409 |
| CHG Index | 5.258 | 1.204 | 1.022 | 1.418 |
| CHG Index | 5.265 | 1.214 | 1.031 | 1.428 |
| CHG Index | 5.272 | 1.223 | 1.041 | 1.437 |
| CHG Index | 5.280 | 1.232 | 1.049 | 1.447 |
| CHG Index | 5.287 | 1.241 | 1.058 | 1.456 |
| CHG Index | 5.295 | 1.250 | 1.067 | 1.465 |
| CHG Index | 5.302 | 1.259 | 1.075 | 1.475 |
| CHG Index | 5.309 | 1.268 | 1.083 | 1.484 |
| CHG Index | 5.317 | 1.276 | 1.091 | 1.494 |
| CHG Index | 5.324 | 1.285 | 1.098 | 1.504 |
| CHG Index | 5.332 | 1.294 | 1.106 | 1.513 |
| CHG Index | 5.339 | 1.302 | 1.113 | 1.524 |
| CHG Index | 5.346 | 1.311 | 1.120 | 1.534 |
| CHG Index | 5.354 | 1.319 | 1.127 | 1.545 |
| CHG Index | 5.361 | 1.328 | 1.133 | 1.555 |
| CHG Index | 5.369 | 1.336 | 1.140 | 1.566 |
| CHG Index | 5.376 | 1.344 | 1.146 | 1.577 |
| CHG Index | 5.383 | 1.353 | 1.152 | 1.588 |
| CHG Index | 5.391 | 1.361 | 1.158 | 1.599 |
| CHG Index | 5.398 | 1.369 | 1.164 | 1.611 |
| CHG Index | 5.406 | 1.377 | 1.170 | 1.622 |
| CHG Index | 5.413 | 1.386 | 1.176 | 1.633 |
| CHG Index | 5.420 | 1.394 | 1.181 | 1.645 |
| CHG Index | 5.428 | 1.402 | 1.187 | 1.656 |
| CHG Index | 5.435 | 1.410 | 1.192 | 1.668 |
| CHG Index | 5.443 | 1.418 | 1.197 | 1.680 |
| CHG Index | 5.450 | 1.426 | 1.203 | 1.691 |
| CHG Index | 5.457 | 1.434 | 1.208 | 1.703 |
| CHG Index | 5.465 | 1.442 | 1.213 | 1.715 |
| CHG Index | 5.472 | 1.450 | 1.218 | 1.726 |
| CHG Index | 5.480 | 1.458 | 1.223 | 1.738 |
| CHG Index | 5.487 | 1.466 | 1.228 | 1.749 |
| CHG Index | 5.494 | 1.473 | 1.233 | 1.761 |
| CHG Index | 5.502 | 1.481 | 1.238 | 1.773 |
| CHG Index | 5.509 | 1.489 | 1.242 | 1.784 |
| CHG Index | 5.517 | 1.496 | 1.247 | 1.796 |
| CHG Index | 5.524 | 1.504 | 1.252 | 1.807 |
| CHG Index | 5.531 | 1.512 | 1.257 | 1.818 |
| CHG Index | 5.539 | 1.519 | 1.261 | 1.830 |
| CHG Index | 5.546 | 1.527 | 1.266 | 1.841 |
| CHG Index | 5.554 | 1.534 | 1.271 | 1.852 |
| CHG Index | 5.561 | 1.541 | 1.275 | 1.863 |
| CHG Index | 5.568 | 1.549 | 1.280 | 1.874 |
| CHG Index | 5.576 | 1.556 | 1.284 | 1.885 |
| CHG Index | 5.583 | 1.563 | 1.289 | 1.896 |
| CHG Index | 5.591 | 1.570 | 1.293 | 1.907 |
| CHG Index | 5.598 | 1.577 | 1.298 | 1.917 |
| CHG Index | 5.605 | 1.584 | 1.302 | 1.928 |
| CHG Index | 5.613 | 1.591 | 1.306 | 1.939 |
| CHG Index | 5.620 | 1.598 | 1.311 | 1.949 |
| CHG Index | 5.627 | 1.605 | 1.315 | 1.960 |
| CHG Index | 5.635 | 1.612 | 1.319 | 1.970 |
| CHG Index | 5.642 | 1.619 | 1.323 | 1.980 |
| CHG Index | 5.650 | 1.626 | 1.328 | 1.990 |
| CHG Index | 5.657 | 1.632 | 1.332 | 2.001 |
| CHG Index | 5.664 | 1.639 | 1.336 | 2.011 |
| CHG Index | 5.672 | 1.646 | 1.340 | 2.021 |
| CHG Index | 5.679 | 1.652 | 1.344 | 2.031 |
| CHG Index | 5.687 | 1.659 | 1.348 | 2.041 |
| CHG Index | 5.694 | 1.665 | 1.352 | 2.050 |
| CHG Index | 5.701 | 1.671 | 1.356 | 2.060 |
| CHG Index | 5.709 | 1.678 | 1.360 | 2.070 |
| CHG Index | 5.716 | 1.684 | 1.363 | 2.080 |
| CHG Index | 5.724 | 1.690 | 1.367 | 2.089 |
| CHG Index | 5.731 | 1.696 | 1.371 | 2.099 |
| CHG Index | 5.738 | 1.702 | 1.374 | 2.108 |
| CHG Index | 5.746 | 1.708 | 1.378 | 2.118 |
| CHG Index | 5.753 | 1.714 | 1.381 | 2.128 |
| CHG Index | 5.761 | 1.720 | 1.385 | 2.137 |
| CHG Index | 5.768 | 1.726 | 1.388 | 2.147 |
| CHG Index | 5.775 | 1.732 | 1.391 | 2.156 |
| CHG Index | 5.783 | 1.738 | 1.394 | 2.166 |
| CHG Index | 5.790 | 1.743 | 1.397 | 2.175 |
| CHG Index | 5.798 | 1.749 | 1.400 | 2.185 |
| CHG Index | 5.805 | 1.754 | 1.403 | 2.194 |
| CHG Index | 5.812 | 1.760 | 1.405 | 2.204 |
| CHG Index | 5.820 | 1.765 | 1.408 | 2.214 |
| CHG Index | 5.827 | 1.771 | 1.410 | 2.223 |
| CHG Index | 5.835 | 1.776 | 1.413 | 2.233 |
| CHG Index | 5.842 | 1.781 | 1.415 | 2.243 |
| CHG Index | 5.849 | 1.787 | 1.417 | 2.253 |
| CHG Index | 5.857 | 1.792 | 1.419 | 2.263 |
| CHG Index | 5.864 | 1.797 | 1.421 | 2.273 |
| CHG Index | 5.872 | 1.802 | 1.422 | 2.283 |
| CHG Index | 5.879 | 1.807 | 1.424 | 2.293 |
| CHG Index | 5.886 | 1.812 | 1.425 | 2.303 |
| CHG Index | 5.894 | 1.817 | 1.427 | 2.314 |
| CHG Index | 5.901 | 1.822 | 1.428 | 2.324 |
| CHG Index | 5.909 | 1.826 | 1.429 | 2.335 |
| CHG Index | 5.916 | 1.831 | 1.430 | 2.346 |
| CHG Index | 5.923 | 1.836 | 1.430 | 2.356 |
| CHG Index | 5.931 | 1.840 | 1.431 | 2.367 |
| CHG Index | 5.938 | 1.845 | 1.431 | 2.379 |
| CHG Index | 5.946 | 1.849 | 1.431 | 2.390 |
| CHG Index | 5.953 | 1.854 | 1.431 | 2.401 |
| CHG Index | 5.960 | 1.858 | 1.431 | 2.413 |
| CHG Index | 5.968 | 1.863 | 1.431 | 2.425 |
| CHG Index | 5.975 | 1.867 | 1.430 | 2.437 |
| CHG Index | 5.983 | 1.871 | 1.430 | 2.449 |
| CHG Index | 5.990 | 1.875 | 1.429 | 2.461 |
| RCII | -0.146 | 0.682 | 0.441 | 1.055 |
| RCII | 0.135 | 0.750 | 0.582 | 0.966 |
| RCII | 0.416 | 0.820 | 0.693 | 0.970 |
| RCII | 0.697 | 0.886 | 0.743 | 1.056 |
| RCII | 0.978 | 0.944 | 0.798 | 1.116 |
| RCII | 1.259 | 0.993 | 0.855 | 1.154 |
| RCII | 1.540 | 1.034 | 0.890 | 1.200 |
| RCII | 1.821 | 1.066 | 0.910 | 1.248 |
| RCII | 2.102 | 1.091 | 0.923 | 1.289 |
| RCII | 2.383 | 1.109 | 0.933 | 1.319 |
| RCII | 2.664 | 1.123 | 0.942 | 1.338 |
| RCII | 2.945 | 1.133 | 0.951 | 1.349 |
| RCII | 3.226 | 1.141 | 0.960 | 1.356 |
| RCII | 3.507 | 1.148 | 0.968 | 1.362 |
| RCII | 3.788 | 1.156 | 0.976 | 1.369 |
| RCII | 4.069 | 1.164 | 0.984 | 1.375 |
| RCII | 4.350 | 1.171 | 0.992 | 1.383 |
| RCII | 4.631 | 1.179 | 1.000 | 1.390 |
| RCII | 4.912 | 1.186 | 1.007 | 1.397 |
| RCII | 5.193 | 1.194 | 1.014 | 1.405 |
| RCII | 5.474 | 1.201 | 1.021 | 1.413 |
| RCII | 5.755 | 1.209 | 1.028 | 1.422 |
| RCII | 6.036 | 1.216 | 1.034 | 1.430 |
| RCII | 6.317 | 1.224 | 1.041 | 1.439 |
| RCII | 6.598 | 1.231 | 1.047 | 1.448 |
| RCII | 6.879 | 1.238 | 1.052 | 1.457 |
| RCII | 7.160 | 1.246 | 1.058 | 1.467 |
| RCII | 7.441 | 1.253 | 1.063 | 1.476 |
| RCII | 7.722 | 1.260 | 1.068 | 1.486 |
| RCII | 8.003 | 1.267 | 1.073 | 1.496 |
| RCII | 8.284 | 1.274 | 1.078 | 1.507 |
| RCII | 8.565 | 1.282 | 1.083 | 1.517 |
| RCII | 8.846 | 1.289 | 1.087 | 1.528 |
| RCII | 9.127 | 1.296 | 1.091 | 1.538 |
| RCII | 9.408 | 1.303 | 1.095 | 1.549 |
| RCII | 9.689 | 1.310 | 1.099 | 1.560 |
| RCII | 9.970 | 1.317 | 1.103 | 1.571 |
| RCII | 10.251 | 1.323 | 1.107 | 1.583 |
| RCII | 10.532 | 1.330 | 1.110 | 1.594 |
| RCII | 10.813 | 1.337 | 1.114 | 1.606 |
| RCII | 11.094 | 1.344 | 1.117 | 1.617 |
| RCII | 11.375 | 1.351 | 1.120 | 1.629 |
| RCII | 11.656 | 1.357 | 1.123 | 1.640 |
| RCII | 11.937 | 1.364 | 1.126 | 1.652 |
| RCII | 12.218 | 1.370 | 1.129 | 1.664 |
| RCII | 12.499 | 1.377 | 1.132 | 1.676 |
| RCII | 12.780 | 1.383 | 1.134 | 1.687 |
| RCII | 13.061 | 1.390 | 1.137 | 1.699 |
| RCII | 13.342 | 1.396 | 1.139 | 1.711 |
| RCII | 13.623 | 1.402 | 1.142 | 1.723 |
| RCII | 13.904 | 1.409 | 1.144 | 1.735 |
| RCII | 14.185 | 1.415 | 1.146 | 1.747 |
| RCII | 14.466 | 1.421 | 1.148 | 1.758 |
| RCII | 14.747 | 1.427 | 1.151 | 1.770 |
| RCII | 15.028 | 1.433 | 1.153 | 1.782 |
| RCII | 15.309 | 1.439 | 1.155 | 1.794 |
| RCII | 15.590 | 1.445 | 1.157 | 1.806 |
| RCII | 15.871 | 1.451 | 1.159 | 1.817 |
| RCII | 16.152 | 1.457 | 1.161 | 1.829 |
| RCII | 16.433 | 1.463 | 1.162 | 1.840 |
| RCII | 16.714 | 1.468 | 1.164 | 1.852 |
| RCII | 16.995 | 1.474 | 1.166 | 1.863 |
| RCII | 17.276 | 1.480 | 1.168 | 1.875 |
| RCII | 17.557 | 1.485 | 1.169 | 1.886 |
| RCII | 17.838 | 1.491 | 1.171 | 1.897 |
| RCII | 18.119 | 1.496 | 1.173 | 1.909 |
| RCII | 18.400 | 1.501 | 1.174 | 1.920 |
| RCII | 18.681 | 1.507 | 1.176 | 1.931 |
| RCII | 18.962 | 1.512 | 1.177 | 1.942 |
| RCII | 19.243 | 1.517 | 1.179 | 1.952 |
| RCII | 19.524 | 1.522 | 1.180 | 1.963 |
| RCII | 19.805 | 1.527 | 1.182 | 1.974 |
| RCII | 20.086 | 1.532 | 1.183 | 1.984 |
| RCII | 20.367 | 1.537 | 1.184 | 1.995 |
| RCII | 20.648 | 1.542 | 1.186 | 2.005 |
| RCII | 20.929 | 1.547 | 1.187 | 2.015 |
| RCII | 21.210 | 1.551 | 1.188 | 2.025 |
| RCII | 21.491 | 1.556 | 1.190 | 2.035 |
| RCII | 21.772 | 1.561 | 1.191 | 2.045 |
| RCII | 22.053 | 1.565 | 1.192 | 2.055 |
| RCII | 22.334 | 1.570 | 1.193 | 2.065 |
| RCII | 22.615 | 1.574 | 1.195 | 2.074 |
| RCII | 22.896 | 1.578 | 1.196 | 2.084 |
| RCII | 23.177 | 1.583 | 1.197 | 2.093 |
| RCII | 23.458 | 1.587 | 1.198 | 2.102 |
| RCII | 23.739 | 1.591 | 1.199 | 2.111 |
| RCII | 24.020 | 1.595 | 1.200 | 2.120 |
| RCII | 24.301 | 1.599 | 1.201 | 2.128 |
| RCII | 24.582 | 1.603 | 1.202 | 2.137 |
| RCII | 24.863 | 1.607 | 1.203 | 2.145 |
| RCII | 25.144 | 1.610 | 1.204 | 2.154 |
| RCII | 25.425 | 1.614 | 1.205 | 2.162 |
| RCII | 25.706 | 1.618 | 1.206 | 2.170 |
| RCII | 25.987 | 1.621 | 1.207 | 2.178 |
| RCII | 26.268 | 1.625 | 1.208 | 2.186 |
| RCII | 26.549 | 1.628 | 1.209 | 2.193 |
| RCII | 26.830 | 1.632 | 1.210 | 2.201 |
| RCII | 27.111 | 1.635 | 1.211 | 2.208 |
| RCII | 27.392 | 1.638 | 1.212 | 2.215 |
| RCII | 27.673 | 1.641 | 1.212 | 2.222 |
| RCII | 27.954 | 1.644 | 1.213 | 2.229 |
| RCII | 28.235 | 1.647 | 1.214 | 2.236 |
| RCII | 28.516 | 1.650 | 1.215 | 2.242 |
| RCII | 28.797 | 1.653 | 1.215 | 2.249 |
| RCII | 29.078 | 1.656 | 1.216 | 2.255 |
| RCII | 29.359 | 1.659 | 1.217 | 2.261 |
| RCII | 29.640 | 1.661 | 1.218 | 2.267 |
| RCII | 29.921 | 1.664 | 1.218 | 2.273 |
| RCII | 30.202 | 1.666 | 1.219 | 2.279 |
| RCII | 30.483 | 1.669 | 1.219 | 2.284 |
| RCII | 30.764 | 1.671 | 1.220 | 2.289 |
| RCII | 31.045 | 1.674 | 1.221 | 2.295 |
| RCII | 31.326 | 1.676 | 1.221 | 2.300 |
| RCII | 31.607 | 1.678 | 1.222 | 2.305 |
| RCII | 31.888 | 1.680 | 1.222 | 2.310 |
| RCII | 32.169 | 1.682 | 1.223 | 2.314 |
| RCII | 32.450 | 1.684 | 1.223 | 2.319 |
| RCII | 32.731 | 1.686 | 1.223 | 2.323 |
| RCII | 33.012 | 1.688 | 1.224 | 2.327 |
| RCII | 33.293 | 1.689 | 1.224 | 2.332 |
| RCII | 33.574 | 1.691 | 1.225 | 2.335 |
| RCII | 33.855 | 1.693 | 1.225 | 2.339 |
| RCII | 34.136 | 1.694 | 1.225 | 2.343 |
| RCII | 34.417 | 1.696 | 1.225 | 2.347 |
| RCII | 34.698 | 1.697 | 1.226 | 2.350 |
| RCII | 34.979 | 1.698 | 1.226 | 2.353 |
| RCII | 35.260 | 1.700 | 1.226 | 2.356 |
| RCII | 35.541 | 1.701 | 1.226 | 2.359 |
| RCII | 35.822 | 1.702 | 1.226 | 2.362 |
| RCII | 36.103 | 1.703 | 1.226 | 2.365 |
| RCII | 36.384 | 1.704 | 1.226 | 2.368 |
| RCII | 36.665 | 1.705 | 1.226 | 2.370 |
| RCII | 36.946 | 1.706 | 1.226 | 2.373 |
| RCII | 37.227 | 1.707 | 1.226 | 2.375 |
| RCII | 37.508 | 1.707 | 1.226 | 2.377 |
| RCII | 37.789 | 1.708 | 1.226 | 2.379 |
| RCII | 38.070 | 1.709 | 1.226 | 2.381 |
| RCII | 38.351 | 1.709 | 1.226 | 2.383 |
| RCII | 38.632 | 1.710 | 1.226 | 2.385 |
| RCII | 38.913 | 1.710 | 1.225 | 2.386 |
| RCII | 39.194 | 1.710 | 1.225 | 2.388 |
| RCII | 39.475 | 1.711 | 1.225 | 2.389 |
| RCII | 39.756 | 1.711 | 1.224 | 2.390 |
| RCII | 40.037 | 1.711 | 1.224 | 2.392 |
| RCII | 40.318 | 1.711 | 1.223 | 2.393 |
| RCII | 40.599 | 1.711 | 1.223 | 2.394 |
| RCII | 40.880 | 1.711 | 1.222 | 2.395 |
| RCII | 41.161 | 1.711 | 1.222 | 2.395 |
| RCII | 41.442 | 1.711 | 1.221 | 2.396 |
| RCII | 41.723 | 1.710 | 1.220 | 2.397 |
| RCII | 42.004 | 1.710 | 1.220 | 2.397 |
| RCII | 42.285 | 1.710 | 1.219 | 2.398 |
| RCII | 42.566 | 1.709 | 1.218 | 2.398 |
| RCII | 42.847 | 1.709 | 1.217 | 2.398 |
| RCII | 43.128 | 1.708 | 1.216 | 2.399 |
| RCII | 43.409 | 1.708 | 1.215 | 2.399 |
| RCII | 43.690 | 1.707 | 1.214 | 2.399 |
| RCII | 43.971 | 1.706 | 1.213 | 2.399 |
| RCII | 44.252 | 1.705 | 1.212 | 2.399 |
| RCII | 44.533 | 1.705 | 1.211 | 2.399 |
| RCII | 44.814 | 1.704 | 1.210 | 2.399 |
| RCII | 45.095 | 1.703 | 1.209 | 2.399 |
| RCII | 45.376 | 1.702 | 1.207 | 2.398 |
| RCII | 45.657 | 1.701 | 1.206 | 2.398 |
| RCII | 45.938 | 1.700 | 1.205 | 2.398 |
| RCII | 46.219 | 1.698 | 1.203 | 2.397 |
| RCII | 46.500 | 1.697 | 1.202 | 2.397 |
| RCII | 46.781 | 1.696 | 1.200 | 2.396 |
| RCII | 47.062 | 1.694 | 1.198 | 2.396 |
| RCII | 47.343 | 1.693 | 1.197 | 2.395 |
| RCII | 47.624 | 1.692 | 1.195 | 2.395 |
| RCII | 47.905 | 1.690 | 1.193 | 2.394 |
| RCII | 48.186 | 1.689 | 1.191 | 2.394 |
| RCII | 48.467 | 1.687 | 1.189 | 2.393 |
| RCII | 48.748 | 1.685 | 1.187 | 2.392 |
| RCII | 49.029 | 1.684 | 1.185 | 2.392 |
| RCII | 49.310 | 1.682 | 1.183 | 2.391 |
| RCII | 49.591 | 1.680 | 1.181 | 2.390 |
| RCII | 49.872 | 1.678 | 1.179 | 2.389 |
| RCII | 50.153 | 1.676 | 1.177 | 2.389 |
| RCII | 50.434 | 1.674 | 1.174 | 2.388 |
| RCII | 50.715 | 1.672 | 1.172 | 2.387 |
| RCII | 50.996 | 1.670 | 1.169 | 2.386 |
| RCII | 51.277 | 1.668 | 1.167 | 2.386 |
| RCII | 51.558 | 1.666 | 1.164 | 2.385 |
| RCII | 51.839 | 1.664 | 1.161 | 2.384 |
| RCII | 52.120 | 1.662 | 1.159 | 2.383 |
| RCII | 52.401 | 1.660 | 1.156 | 2.383 |
| RCII | 52.682 | 1.657 | 1.153 | 2.382 |
| RCII | 52.963 | 1.655 | 1.150 | 2.381 |
| RCII | 53.244 | 1.653 | 1.147 | 2.380 |
| RCII | 53.525 | 1.650 | 1.144 | 2.380 |
| RCII | 53.806 | 1.648 | 1.141 | 2.379 |
| RCII | 54.087 | 1.645 | 1.138 | 2.378 |
| RCII | 54.368 | 1.643 | 1.135 | 2.378 |
| RCII | 54.649 | 1.640 | 1.131 | 2.377 |
| RCII | 54.930 | 1.637 | 1.128 | 2.377 |
| RCII | 55.211 | 1.635 | 1.125 | 2.376 |
| RCII | 55.492 | 1.632 | 1.121 | 2.376 |
| RCII | 55.773 | 1.629 | 1.118 | 2.375 |
| hs-CRP/HDL-C | 0.003 | 0.649 | 0.457 | 0.922 |
| hs-CRP/HDL-C | 0.006 | 0.765 | 0.642 | 0.911 |
| hs-CRP/HDL-C | 0.010 | 0.874 | 0.733 | 1.042 |
| hs-CRP/HDL-C | 0.013 | 0.956 | 0.810 | 1.128 |
| hs-CRP/HDL-C | 0.016 | 1.014 | 0.874 | 1.177 |
| hs-CRP/HDL-C | 0.020 | 1.057 | 0.906 | 1.234 |
| hs-CRP/HDL-C | 0.023 | 1.086 | 0.920 | 1.281 |
| hs-CRP/HDL-C | 0.027 | 1.103 | 0.929 | 1.311 |
| hs-CRP/HDL-C | 0.030 | 1.112 | 0.935 | 1.324 |
| hs-CRP/HDL-C | 0.033 | 1.116 | 0.940 | 1.327 |
| hs-CRP/HDL-C | 0.037 | 1.119 | 0.944 | 1.327 |
| hs-CRP/HDL-C | 0.040 | 1.121 | 0.947 | 1.327 |
| hs-CRP/HDL-C | 0.044 | 1.124 | 0.951 | 1.328 |
| hs-CRP/HDL-C | 0.047 | 1.126 | 0.954 | 1.329 |
| hs-CRP/HDL-C | 0.050 | 1.128 | 0.957 | 1.331 |
| hs-CRP/HDL-C | 0.054 | 1.131 | 0.959 | 1.333 |
| hs-CRP/HDL-C | 0.057 | 1.133 | 0.961 | 1.336 |
| hs-CRP/HDL-C | 0.061 | 1.135 | 0.963 | 1.339 |
| hs-CRP/HDL-C | 0.064 | 1.138 | 0.964 | 1.343 |
| hs-CRP/HDL-C | 0.067 | 1.140 | 0.965 | 1.347 |
| hs-CRP/HDL-C | 0.071 | 1.142 | 0.966 | 1.351 |
| hs-CRP/HDL-C | 0.074 | 1.144 | 0.966 | 1.356 |
| hs-CRP/HDL-C | 0.078 | 1.147 | 0.966 | 1.360 |
| hs-CRP/HDL-C | 0.081 | 1.149 | 0.966 | 1.366 |
| hs-CRP/HDL-C | 0.084 | 1.151 | 0.966 | 1.371 |
| hs-CRP/HDL-C | 0.088 | 1.153 | 0.966 | 1.377 |
| hs-CRP/HDL-C | 0.091 | 1.155 | 0.965 | 1.383 |
| hs-CRP/HDL-C | 0.095 | 1.157 | 0.964 | 1.389 |
| hs-CRP/HDL-C | 0.098 | 1.159 | 0.963 | 1.395 |
| hs-CRP/HDL-C | 0.101 | 1.162 | 0.962 | 1.402 |
| hs-CRP/HDL-C | 0.105 | 1.164 | 0.961 | 1.409 |
| hs-CRP/HDL-C | 0.108 | 1.166 | 0.960 | 1.416 |
| hs-CRP/HDL-C | 0.111 | 1.168 | 0.959 | 1.422 |
| hs-CRP/HDL-C | 0.115 | 1.170 | 0.957 | 1.430 |
| hs-CRP/HDL-C | 0.118 | 1.172 | 0.956 | 1.437 |
| hs-CRP/HDL-C | 0.122 | 1.174 | 0.954 | 1.444 |
| hs-CRP/HDL-C | 0.125 | 1.176 | 0.952 | 1.451 |
| hs-CRP/HDL-C | 0.128 | 1.178 | 0.951 | 1.458 |
| hs-CRP/HDL-C | 0.132 | 1.179 | 0.949 | 1.466 |
| hs-CRP/HDL-C | 0.135 | 1.181 | 0.947 | 1.473 |
| hs-CRP/HDL-C | 0.139 | 1.183 | 0.946 | 1.481 |
| hs-CRP/HDL-C | 0.142 | 1.185 | 0.944 | 1.488 |
| hs-CRP/HDL-C | 0.145 | 1.187 | 0.942 | 1.495 |
| hs-CRP/HDL-C | 0.149 | 1.189 | 0.940 | 1.503 |
| hs-CRP/HDL-C | 0.152 | 1.191 | 0.939 | 1.510 |
| hs-CRP/HDL-C | 0.156 | 1.192 | 0.937 | 1.518 |
| hs-CRP/HDL-C | 0.159 | 1.194 | 0.935 | 1.525 |
| hs-CRP/HDL-C | 0.162 | 1.196 | 0.933 | 1.532 |
| hs-CRP/HDL-C | 0.166 | 1.198 | 0.932 | 1.539 |
| hs-CRP/HDL-C | 0.169 | 1.199 | 0.930 | 1.547 |
| hs-CRP/HDL-C | 0.173 | 1.201 | 0.928 | 1.554 |
| hs-CRP/HDL-C | 0.176 | 1.203 | 0.927 | 1.561 |
| hs-CRP/HDL-C | 0.179 | 1.204 | 0.925 | 1.568 |
| hs-CRP/HDL-C | 0.183 | 1.206 | 0.923 | 1.575 |
| hs-CRP/HDL-C | 0.186 | 1.208 | 0.922 | 1.582 |
| hs-CRP/HDL-C | 0.190 | 1.209 | 0.920 | 1.589 |
| hs-CRP/HDL-C | 0.193 | 1.211 | 0.919 | 1.596 |
| hs-CRP/HDL-C | 0.196 | 1.212 | 0.917 | 1.602 |
| hs-CRP/HDL-C | 0.200 | 1.214 | 0.916 | 1.609 |
| hs-CRP/HDL-C | 0.203 | 1.215 | 0.914 | 1.616 |
| hs-CRP/HDL-C | 0.207 | 1.217 | 0.913 | 1.622 |
| hs-CRP/HDL-C | 0.210 | 1.218 | 0.912 | 1.628 |
| hs-CRP/HDL-C | 0.213 | 1.220 | 0.910 | 1.635 |
| hs-CRP/HDL-C | 0.217 | 1.221 | 0.909 | 1.641 |
| hs-CRP/HDL-C | 0.220 | 1.223 | 0.908 | 1.647 |
| hs-CRP/HDL-C | 0.223 | 1.224 | 0.907 | 1.653 |
| hs-CRP/HDL-C | 0.227 | 1.226 | 0.905 | 1.659 |
| hs-CRP/HDL-C | 0.230 | 1.227 | 0.904 | 1.665 |
| hs-CRP/HDL-C | 0.234 | 1.228 | 0.903 | 1.671 |
| hs-CRP/HDL-C | 0.237 | 1.230 | 0.902 | 1.676 |
| hs-CRP/HDL-C | 0.240 | 1.231 | 0.901 | 1.682 |
| hs-CRP/HDL-C | 0.244 | 1.232 | 0.900 | 1.687 |
| hs-CRP/HDL-C | 0.247 | 1.234 | 0.899 | 1.693 |
| hs-CRP/HDL-C | 0.251 | 1.235 | 0.898 | 1.698 |
| hs-CRP/HDL-C | 0.254 | 1.236 | 0.897 | 1.703 |
| hs-CRP/HDL-C | 0.257 | 1.237 | 0.897 | 1.708 |
| hs-CRP/HDL-C | 0.261 | 1.239 | 0.896 | 1.713 |
| hs-CRP/HDL-C | 0.264 | 1.240 | 0.895 | 1.718 |
| hs-CRP/HDL-C | 0.268 | 1.241 | 0.894 | 1.723 |
| hs-CRP/HDL-C | 0.271 | 1.242 | 0.894 | 1.727 |
| hs-CRP/HDL-C | 0.274 | 1.243 | 0.893 | 1.732 |
| hs-CRP/HDL-C | 0.278 | 1.245 | 0.892 | 1.736 |
| hs-CRP/HDL-C | 0.281 | 1.246 | 0.892 | 1.741 |
| hs-CRP/HDL-C | 0.285 | 1.247 | 0.891 | 1.745 |
| hs-CRP/HDL-C | 0.288 | 1.248 | 0.891 | 1.749 |
| hs-CRP/HDL-C | 0.291 | 1.249 | 0.890 | 1.753 |
| hs-CRP/HDL-C | 0.295 | 1.250 | 0.890 | 1.757 |
| hs-CRP/HDL-C | 0.298 | 1.251 | 0.889 | 1.761 |
| hs-CRP/HDL-C | 0.302 | 1.252 | 0.889 | 1.764 |
| hs-CRP/HDL-C | 0.305 | 1.253 | 0.888 | 1.768 |
| hs-CRP/HDL-C | 0.308 | 1.254 | 0.888 | 1.771 |
| hs-CRP/HDL-C | 0.312 | 1.255 | 0.888 | 1.775 |
| hs-CRP/HDL-C | 0.315 | 1.256 | 0.887 | 1.778 |
| hs-CRP/HDL-C | 0.318 | 1.257 | 0.887 | 1.781 |
| hs-CRP/HDL-C | 0.322 | 1.258 | 0.887 | 1.784 |
| hs-CRP/HDL-C | 0.325 | 1.259 | 0.887 | 1.787 |
| hs-CRP/HDL-C | 0.329 | 1.260 | 0.886 | 1.790 |
| hs-CRP/HDL-C | 0.332 | 1.261 | 0.886 | 1.793 |
| hs-CRP/HDL-C | 0.335 | 1.261 | 0.886 | 1.796 |
| hs-CRP/HDL-C | 0.339 | 1.262 | 0.886 | 1.798 |
| hs-CRP/HDL-C | 0.342 | 1.263 | 0.886 | 1.801 |
| hs-CRP/HDL-C | 0.346 | 1.264 | 0.886 | 1.804 |
| hs-CRP/HDL-C | 0.349 | 1.265 | 0.886 | 1.806 |
| hs-CRP/HDL-C | 0.352 | 1.266 | 0.886 | 1.808 |
| hs-CRP/HDL-C | 0.356 | 1.266 | 0.886 | 1.810 |
| hs-CRP/HDL-C | 0.359 | 1.267 | 0.886 | 1.812 |
| hs-CRP/HDL-C | 0.363 | 1.268 | 0.886 | 1.815 |
| hs-CRP/HDL-C | 0.366 | 1.269 | 0.886 | 1.816 |
| hs-CRP/HDL-C | 0.369 | 1.269 | 0.886 | 1.818 |
| hs-CRP/HDL-C | 0.373 | 1.270 | 0.886 | 1.820 |
| hs-CRP/HDL-C | 0.376 | 1.271 | 0.886 | 1.822 |
| hs-CRP/HDL-C | 0.380 | 1.271 | 0.886 | 1.824 |
| hs-CRP/HDL-C | 0.383 | 1.272 | 0.886 | 1.825 |
| hs-CRP/HDL-C | 0.386 | 1.273 | 0.887 | 1.827 |
| hs-CRP/HDL-C | 0.390 | 1.273 | 0.887 | 1.828 |
| hs-CRP/HDL-C | 0.393 | 1.274 | 0.887 | 1.829 |
| hs-CRP/HDL-C | 0.397 | 1.274 | 0.887 | 1.831 |
| hs-CRP/HDL-C | 0.400 | 1.275 | 0.887 | 1.832 |
| hs-CRP/HDL-C | 0.403 | 1.275 | 0.887 | 1.833 |
| hs-CRP/HDL-C | 0.407 | 1.276 | 0.888 | 1.834 |
| hs-CRP/HDL-C | 0.410 | 1.277 | 0.888 | 1.836 |
| hs-CRP/HDL-C | 0.414 | 1.277 | 0.888 | 1.837 |
| hs-CRP/HDL-C | 0.417 | 1.278 | 0.888 | 1.838 |
| hs-CRP/HDL-C | 0.420 | 1.278 | 0.888 | 1.839 |
| hs-CRP/HDL-C | 0.424 | 1.278 | 0.889 | 1.839 |
| hs-CRP/HDL-C | 0.427 | 1.279 | 0.889 | 1.840 |
| hs-CRP/HDL-C | 0.430 | 1.279 | 0.889 | 1.841 |
| hs-CRP/HDL-C | 0.434 | 1.280 | 0.889 | 1.842 |
| hs-CRP/HDL-C | 0.437 | 1.280 | 0.889 | 1.843 |
| hs-CRP/HDL-C | 0.441 | 1.281 | 0.890 | 1.843 |
| hs-CRP/HDL-C | 0.444 | 1.281 | 0.890 | 1.844 |
| hs-CRP/HDL-C | 0.447 | 1.281 | 0.890 | 1.845 |
| hs-CRP/HDL-C | 0.451 | 1.282 | 0.890 | 1.846 |
| hs-CRP/HDL-C | 0.454 | 1.282 | 0.890 | 1.846 |
| hs-CRP/HDL-C | 0.458 | 1.282 | 0.891 | 1.847 |
| hs-CRP/HDL-C | 0.461 | 1.283 | 0.891 | 1.847 |
| hs-CRP/HDL-C | 0.464 | 1.283 | 0.891 | 1.848 |
| hs-CRP/HDL-C | 0.468 | 1.283 | 0.891 | 1.849 |
| hs-CRP/HDL-C | 0.471 | 1.284 | 0.891 | 1.849 |
| hs-CRP/HDL-C | 0.475 | 1.284 | 0.891 | 1.850 |
| hs-CRP/HDL-C | 0.478 | 1.284 | 0.891 | 1.850 |
| hs-CRP/HDL-C | 0.481 | 1.284 | 0.891 | 1.851 |
| hs-CRP/HDL-C | 0.485 | 1.285 | 0.891 | 1.852 |
| hs-CRP/HDL-C | 0.488 | 1.285 | 0.891 | 1.852 |
| hs-CRP/HDL-C | 0.492 | 1.285 | 0.891 | 1.853 |
| hs-CRP/HDL-C | 0.495 | 1.285 | 0.891 | 1.854 |
| hs-CRP/HDL-C | 0.498 | 1.286 | 0.891 | 1.854 |
| hs-CRP/HDL-C | 0.502 | 1.286 | 0.891 | 1.855 |
| hs-CRP/HDL-C | 0.505 | 1.286 | 0.891 | 1.856 |
| hs-CRP/HDL-C | 0.509 | 1.286 | 0.891 | 1.857 |
| hs-CRP/HDL-C | 0.512 | 1.286 | 0.891 | 1.857 |
| hs-CRP/HDL-C | 0.515 | 1.286 | 0.890 | 1.858 |
| hs-CRP/HDL-C | 0.519 | 1.286 | 0.890 | 1.859 |
| hs-CRP/HDL-C | 0.522 | 1.287 | 0.890 | 1.860 |
| hs-CRP/HDL-C | 0.525 | 1.287 | 0.890 | 1.861 |
| hs-CRP/HDL-C | 0.529 | 1.287 | 0.889 | 1.862 |
| hs-CRP/HDL-C | 0.532 | 1.287 | 0.889 | 1.863 |
| hs-CRP/HDL-C | 0.536 | 1.287 | 0.888 | 1.864 |
| hs-CRP/HDL-C | 0.539 | 1.287 | 0.888 | 1.865 |
| hs-CRP/HDL-C | 0.542 | 1.287 | 0.887 | 1.866 |
| hs-CRP/HDL-C | 0.546 | 1.287 | 0.887 | 1.868 |
| hs-CRP/HDL-C | 0.549 | 1.287 | 0.886 | 1.869 |
| hs-CRP/HDL-C | 0.553 | 1.287 | 0.886 | 1.870 |
| hs-CRP/HDL-C | 0.556 | 1.287 | 0.885 | 1.872 |
| hs-CRP/HDL-C | 0.559 | 1.287 | 0.884 | 1.873 |
| hs-CRP/HDL-C | 0.563 | 1.287 | 0.883 | 1.875 |
| hs-CRP/HDL-C | 0.566 | 1.287 | 0.883 | 1.877 |
| hs-CRP/HDL-C | 0.570 | 1.287 | 0.882 | 1.879 |
| hs-CRP/HDL-C | 0.573 | 1.287 | 0.881 | 1.880 |
| hs-CRP/HDL-C | 0.576 | 1.287 | 0.880 | 1.882 |
| hs-CRP/HDL-C | 0.580 | 1.287 | 0.879 | 1.884 |
| hs-CRP/HDL-C | 0.583 | 1.287 | 0.878 | 1.887 |
| hs-CRP/HDL-C | 0.587 | 1.287 | 0.877 | 1.889 |
| hs-CRP/HDL-C | 0.590 | 1.287 | 0.875 | 1.891 |
| hs-CRP/HDL-C | 0.593 | 1.287 | 0.874 | 1.893 |
| hs-CRP/HDL-C | 0.597 | 1.286 | 0.873 | 1.896 |
| hs-CRP/HDL-C | 0.600 | 1.286 | 0.872 | 1.899 |
| hs-CRP/HDL-C | 0.604 | 1.286 | 0.870 | 1.901 |
| hs-CRP/HDL-C | 0.607 | 1.286 | 0.869 | 1.904 |
| hs-CRP/HDL-C | 0.610 | 1.286 | 0.867 | 1.907 |
| hs-CRP/HDL-C | 0.614 | 1.286 | 0.866 | 1.910 |
| hs-CRP/HDL-C | 0.617 | 1.286 | 0.864 | 1.913 |
| hs-CRP/HDL-C | 0.621 | 1.286 | 0.862 | 1.916 |
| hs-CRP/HDL-C | 0.624 | 1.285 | 0.861 | 1.920 |
| hs-CRP/HDL-C | 0.627 | 1.285 | 0.859 | 1.923 |
| hs-CRP/HDL-C | 0.631 | 1.285 | 0.857 | 1.927 |
| hs-CRP/HDL-C | 0.634 | 1.285 | 0.855 | 1.930 |
| hs-CRP/HDL-C | 0.637 | 1.285 | 0.853 | 1.934 |
| hs-CRP/HDL-C | 0.641 | 1.284 | 0.851 | 1.938 |
| hs-CRP/HDL-C | 0.644 | 1.284 | 0.849 | 1.942 |
| hs-CRP/HDL-C | 0.648 | 1.284 | 0.847 | 1.946 |
| hs-CRP/HDL-C | 0.651 | 1.284 | 0.845 | 1.951 |
| hs-CRP/HDL-C | 0.654 | 1.283 | 0.843 | 1.955 |
| hs-CRP/HDL-C | 0.658 | 1.283 | 0.840 | 1.960 |
| hs-CRP/HDL-C | 0.661 | 1.283 | 0.838 | 1.964 |
| hs-CRP/HDL-C | 0.665 | 1.283 | 0.836 | 1.969 |
| hs-CRP/HDL-C | 0.668 | 1.283 | 0.833 | 1.974 |
| hs-CRP/HDL-C | 0.671 | 1.282 | 0.831 | 1.979 |
| hs-CRP/HDL-C | 0.675 | 1.282 | 0.828 | 1.984 |
| hs-CRP/HDL-C | 0.678 | 1.282 | 0.826 | 1.989 |
| CTI | 7.100 | 0.714 | 0.428 | 1.188 |
| CTI | 7.116 | 0.714 | 0.435 | 1.172 |
| CTI | 7.132 | 0.715 | 0.442 | 1.156 |
| CTI | 7.148 | 0.716 | 0.449 | 1.141 |
| CTI | 7.164 | 0.716 | 0.456 | 1.126 |
| CTI | 7.180 | 0.717 | 0.463 | 1.111 |
| CTI | 7.196 | 0.718 | 0.470 | 1.097 |
| CTI | 7.212 | 0.719 | 0.477 | 1.084 |
| CTI | 7.228 | 0.719 | 0.483 | 1.070 |
| CTI | 7.244 | 0.720 | 0.490 | 1.058 |
| CTI | 7.260 | 0.721 | 0.497 | 1.045 |
| CTI | 7.276 | 0.722 | 0.504 | 1.033 |
| CTI | 7.292 | 0.723 | 0.511 | 1.022 |
| CTI | 7.308 | 0.724 | 0.518 | 1.011 |
| CTI | 7.324 | 0.724 | 0.524 | 1.001 |
| CTI | 7.340 | 0.725 | 0.531 | 0.992 |
| CTI | 7.356 | 0.726 | 0.537 | 0.983 |
| CTI | 7.372 | 0.728 | 0.543 | 0.974 |
| CTI | 7.388 | 0.729 | 0.549 | 0.966 |
| CTI | 7.404 | 0.730 | 0.555 | 0.959 |
| CTI | 7.420 | 0.731 | 0.561 | 0.953 |
| CTI | 7.436 | 0.732 | 0.566 | 0.947 |
| CTI | 7.452 | 0.734 | 0.572 | 0.941 |
| CTI | 7.468 | 0.735 | 0.577 | 0.937 |
| CTI | 7.484 | 0.736 | 0.581 | 0.933 |
| CTI | 7.500 | 0.738 | 0.586 | 0.930 |
| CTI | 7.516 | 0.740 | 0.590 | 0.927 |
| CTI | 7.532 | 0.741 | 0.594 | 0.925 |
| CTI | 7.548 | 0.743 | 0.598 | 0.924 |
| CTI | 7.564 | 0.745 | 0.601 | 0.923 |
| CTI | 7.580 | 0.747 | 0.604 | 0.923 |
| CTI | 7.596 | 0.749 | 0.607 | 0.923 |
| CTI | 7.612 | 0.751 | 0.610 | 0.924 |
| CTI | 7.628 | 0.753 | 0.613 | 0.925 |
| CTI | 7.644 | 0.755 | 0.615 | 0.927 |
| CTI | 7.660 | 0.758 | 0.618 | 0.929 |
| CTI | 7.676 | 0.760 | 0.620 | 0.932 |
| CTI | 7.692 | 0.763 | 0.622 | 0.935 |
| CTI | 7.708 | 0.765 | 0.625 | 0.938 |
| CTI | 7.724 | 0.768 | 0.627 | 0.942 |
| CTI | 7.740 | 0.771 | 0.629 | 0.945 |
| CTI | 7.756 | 0.774 | 0.632 | 0.949 |
| CTI | 7.772 | 0.778 | 0.634 | 0.953 |
| CTI | 7.788 | 0.781 | 0.637 | 0.957 |
| CTI | 7.804 | 0.784 | 0.640 | 0.961 |
| CTI | 7.820 | 0.788 | 0.643 | 0.965 |
| CTI | 7.836 | 0.792 | 0.646 | 0.970 |
| CTI | 7.852 | 0.796 | 0.650 | 0.974 |
| CTI | 7.868 | 0.800 | 0.654 | 0.978 |
| CTI | 7.884 | 0.804 | 0.658 | 0.982 |
| CTI | 7.900 | 0.808 | 0.663 | 0.986 |
| CTI | 7.916 | 0.813 | 0.668 | 0.990 |
| CTI | 7.932 | 0.818 | 0.673 | 0.994 |
| CTI | 7.948 | 0.823 | 0.678 | 0.997 |
| CTI | 7.964 | 0.828 | 0.684 | 1.001 |
| CTI | 7.980 | 0.833 | 0.691 | 1.005 |
| CTI | 7.996 | 0.839 | 0.697 | 1.009 |
| CTI | 8.012 | 0.844 | 0.704 | 1.012 |
| CTI | 8.028 | 0.850 | 0.711 | 1.016 |
| CTI | 8.044 | 0.856 | 0.718 | 1.020 |
| CTI | 8.060 | 0.862 | 0.725 | 1.025 |
| CTI | 8.076 | 0.869 | 0.733 | 1.029 |
| CTI | 8.092 | 0.875 | 0.740 | 1.034 |
| CTI | 8.108 | 0.882 | 0.747 | 1.040 |
| CTI | 8.124 | 0.888 | 0.755 | 1.046 |
| CTI | 8.141 | 0.895 | 0.762 | 1.052 |
| CTI | 8.157 | 0.902 | 0.769 | 1.059 |
| CTI | 8.173 | 0.909 | 0.775 | 1.066 |
| CTI | 8.189 | 0.916 | 0.782 | 1.074 |
| CTI | 8.205 | 0.923 | 0.788 | 1.082 |
| CTI | 8.221 | 0.931 | 0.794 | 1.091 |
| CTI | 8.237 | 0.938 | 0.800 | 1.101 |
| CTI | 8.253 | 0.945 | 0.805 | 1.110 |
| CTI | 8.269 | 0.953 | 0.810 | 1.120 |
| CTI | 8.285 | 0.960 | 0.816 | 1.131 |
| CTI | 8.301 | 0.968 | 0.821 | 1.141 |
| CTI | 8.317 | 0.975 | 0.826 | 1.152 |
| CTI | 8.333 | 0.983 | 0.831 | 1.163 |
| CTI | 8.349 | 0.990 | 0.836 | 1.173 |
| CTI | 8.365 | 0.998 | 0.841 | 1.184 |
| CTI | 8.381 | 1.005 | 0.846 | 1.194 |
| CTI | 8.397 | 1.013 | 0.852 | 1.204 |
| CTI | 8.413 | 1.020 | 0.857 | 1.213 |
| CTI | 8.429 | 1.027 | 0.863 | 1.222 |
| CTI | 8.445 | 1.034 | 0.869 | 1.231 |
| CTI | 8.461 | 1.042 | 0.875 | 1.240 |
| CTI | 8.477 | 1.049 | 0.881 | 1.248 |
| CTI | 8.493 | 1.056 | 0.888 | 1.256 |
| CTI | 8.509 | 1.063 | 0.894 | 1.263 |
| CTI | 8.525 | 1.070 | 0.901 | 1.271 |
| CTI | 8.541 | 1.077 | 0.908 | 1.278 |
| CTI | 8.557 | 1.084 | 0.915 | 1.284 |
| CTI | 8.573 | 1.091 | 0.922 | 1.291 |
| CTI | 8.589 | 1.098 | 0.929 | 1.298 |
| CTI | 8.605 | 1.105 | 0.936 | 1.304 |
| CTI | 8.621 | 1.112 | 0.943 | 1.310 |
| CTI | 8.637 | 1.118 | 0.950 | 1.317 |
| CTI | 8.653 | 1.125 | 0.957 | 1.323 |
| CTI | 8.669 | 1.132 | 0.964 | 1.329 |
| CTI | 8.685 | 1.139 | 0.970 | 1.336 |
| CTI | 8.701 | 1.145 | 0.977 | 1.343 |
| CTI | 8.717 | 1.152 | 0.984 | 1.349 |
| CTI | 8.733 | 1.159 | 0.990 | 1.356 |
| CTI | 8.749 | 1.165 | 0.996 | 1.363 |
| CTI | 8.765 | 1.172 | 1.002 | 1.371 |
| CTI | 8.781 | 1.179 | 1.008 | 1.378 |
| CTI | 8.797 | 1.185 | 1.014 | 1.386 |
| CTI | 8.813 | 1.192 | 1.019 | 1.394 |
| CTI | 8.829 | 1.199 | 1.025 | 1.402 |
| CTI | 8.845 | 1.205 | 1.030 | 1.411 |
| CTI | 8.861 | 1.212 | 1.035 | 1.419 |
| CTI | 8.877 | 1.219 | 1.040 | 1.428 |
| CTI | 8.893 | 1.225 | 1.045 | 1.437 |
| CTI | 8.909 | 1.232 | 1.049 | 1.446 |
| CTI | 8.925 | 1.239 | 1.054 | 1.455 |
| CTI | 8.941 | 1.245 | 1.059 | 1.465 |
| CTI | 8.957 | 1.252 | 1.063 | 1.474 |
| CTI | 8.973 | 1.259 | 1.068 | 1.484 |
| CTI | 8.989 | 1.265 | 1.072 | 1.493 |
| CTI | 9.005 | 1.272 | 1.076 | 1.503 |
| CTI | 9.021 | 1.279 | 1.081 | 1.512 |
| CTI | 9.037 | 1.285 | 1.085 | 1.522 |
| CTI | 9.053 | 1.292 | 1.090 | 1.532 |
| CTI | 9.069 | 1.298 | 1.094 | 1.541 |
| CTI | 9.085 | 1.305 | 1.098 | 1.551 |
| CTI | 9.101 | 1.312 | 1.103 | 1.561 |
| CTI | 9.117 | 1.318 | 1.107 | 1.570 |
| CTI | 9.133 | 1.325 | 1.111 | 1.580 |
| CTI | 9.149 | 1.332 | 1.116 | 1.589 |
| CTI | 9.165 | 1.338 | 1.120 | 1.599 |
| CTI | 9.181 | 1.345 | 1.125 | 1.608 |
| CTI | 9.197 | 1.352 | 1.129 | 1.618 |
| CTI | 9.213 | 1.358 | 1.134 | 1.627 |
| CTI | 9.229 | 1.365 | 1.138 | 1.637 |
| CTI | 9.245 | 1.371 | 1.143 | 1.646 |
| CTI | 9.261 | 1.378 | 1.147 | 1.655 |
| CTI | 9.277 | 1.385 | 1.152 | 1.665 |
| CTI | 9.293 | 1.391 | 1.157 | 1.674 |
| CTI | 9.309 | 1.398 | 1.161 | 1.683 |
| CTI | 9.325 | 1.405 | 1.166 | 1.692 |
| CTI | 9.341 | 1.411 | 1.171 | 1.701 |
| CTI | 9.357 | 1.418 | 1.176 | 1.710 |
| CTI | 9.373 | 1.424 | 1.180 | 1.719 |
| CTI | 9.389 | 1.431 | 1.185 | 1.728 |
| CTI | 9.405 | 1.438 | 1.190 | 1.737 |
| CTI | 9.421 | 1.444 | 1.195 | 1.745 |
| CTI | 9.437 | 1.451 | 1.200 | 1.754 |
| CTI | 9.453 | 1.457 | 1.205 | 1.763 |
| CTI | 9.469 | 1.464 | 1.210 | 1.772 |
| CTI | 9.485 | 1.471 | 1.215 | 1.780 |
| CTI | 9.501 | 1.477 | 1.220 | 1.789 |
| CTI | 9.517 | 1.484 | 1.225 | 1.798 |
| CTI | 9.533 | 1.490 | 1.230 | 1.807 |
| CTI | 9.550 | 1.497 | 1.235 | 1.815 |
| CTI | 9.566 | 1.504 | 1.239 | 1.824 |
| CTI | 9.582 | 1.510 | 1.244 | 1.833 |
| CTI | 9.598 | 1.517 | 1.249 | 1.842 |
| CTI | 9.614 | 1.523 | 1.254 | 1.851 |
| CTI | 9.630 | 1.530 | 1.259 | 1.859 |
| CTI | 9.646 | 1.537 | 1.264 | 1.868 |
| CTI | 9.662 | 1.543 | 1.268 | 1.877 |
| CTI | 9.678 | 1.550 | 1.273 | 1.887 |
| CTI | 9.694 | 1.556 | 1.277 | 1.896 |
| CTI | 9.710 | 1.563 | 1.282 | 1.905 |
| CTI | 9.726 | 1.569 | 1.286 | 1.915 |
| CTI | 9.742 | 1.576 | 1.291 | 1.924 |
| CTI | 9.758 | 1.583 | 1.295 | 1.934 |
| CTI | 9.774 | 1.589 | 1.299 | 1.944 |
| CTI | 9.790 | 1.596 | 1.303 | 1.954 |
| CTI | 9.806 | 1.602 | 1.307 | 1.964 |
| CTI | 9.822 | 1.609 | 1.311 | 1.975 |
| CTI | 9.838 | 1.615 | 1.314 | 1.985 |
| CTI | 9.854 | 1.622 | 1.318 | 1.996 |
| CTI | 9.870 | 1.628 | 1.321 | 2.007 |
| CTI | 9.886 | 1.635 | 1.324 | 2.018 |
| CTI | 9.902 | 1.642 | 1.328 | 2.030 |
| CTI | 9.918 | 1.648 | 1.330 | 2.042 |
| CTI | 9.934 | 1.655 | 1.333 | 2.054 |
| CTI | 9.950 | 1.661 | 1.336 | 2.066 |
| CTI | 9.966 | 1.668 | 1.338 | 2.078 |
| CTI | 9.982 | 1.674 | 1.340 | 2.091 |
| CTI | 9.998 | 1.681 | 1.342 | 2.105 |
| CTI | 10.014 | 1.687 | 1.344 | 2.118 |
| CTI | 10.030 | 1.694 | 1.346 | 2.132 |
| CTI | 10.046 | 1.701 | 1.348 | 2.146 |
| CTI | 10.062 | 1.707 | 1.349 | 2.160 |
| CTI | 10.078 | 1.714 | 1.350 | 2.175 |
| CTI | 10.094 | 1.720 | 1.351 | 2.190 |
| CTI | 10.110 | 1.727 | 1.352 | 2.206 |
| CTI | 10.126 | 1.733 | 1.352 | 2.222 |
| CTI | 10.142 | 1.740 | 1.353 | 2.238 |
| CTI | 10.158 | 1.746 | 1.353 | 2.255 |
| CTI | 10.174 | 1.753 | 1.353 | 2.272 |
| CTI | 10.190 | 1.760 | 1.353 | 2.289 |
| CTI | 10.206 | 1.766 | 1.352 | 2.307 |
| CTI | 10.222 | 1.773 | 1.352 | 2.325 |
| CTI | 10.238 | 1.779 | 1.351 | 2.343 |
| CTI | 10.254 | 1.786 | 1.350 | 2.362 |
| CTI | 10.270 | 1.792 | 1.349 | 2.382 |
| CTI | 10.286 | 1.799 | 1.348 | 2.402 |
| TyG-BMI | 132.169 | 0.179 | 0.079 | 0.404 |
| TyG-BMI | 132.929 | 0.182 | 0.083 | 0.401 |
| TyG-BMI | 133.689 | 0.185 | 0.086 | 0.399 |
| TyG-BMI | 134.450 | 0.188 | 0.090 | 0.396 |
| TyG-BMI | 135.210 | 0.192 | 0.093 | 0.393 |
| TyG-BMI | 135.970 | 0.195 | 0.097 | 0.391 |
| TyG-BMI | 136.730 | 0.198 | 0.101 | 0.388 |
| TyG-BMI | 137.491 | 0.201 | 0.105 | 0.386 |
| TyG-BMI | 138.251 | 0.205 | 0.109 | 0.384 |
| TyG-BMI | 139.011 | 0.208 | 0.114 | 0.382 |
| TyG-BMI | 139.772 | 0.212 | 0.118 | 0.381 |
| TyG-BMI | 140.532 | 0.216 | 0.123 | 0.379 |
| TyG-BMI | 141.292 | 0.220 | 0.128 | 0.378 |
| TyG-BMI | 142.053 | 0.224 | 0.132 | 0.377 |
| TyG-BMI | 142.813 | 0.228 | 0.137 | 0.377 |
| TyG-BMI | 143.573 | 0.232 | 0.142 | 0.377 |
| TyG-BMI | 144.334 | 0.236 | 0.148 | 0.377 |
| TyG-BMI | 145.094 | 0.240 | 0.153 | 0.377 |
| TyG-BMI | 145.854 | 0.245 | 0.158 | 0.378 |
| TyG-BMI | 146.615 | 0.249 | 0.164 | 0.380 |
| TyG-BMI | 147.375 | 0.254 | 0.169 | 0.381 |
| TyG-BMI | 148.135 | 0.259 | 0.175 | 0.383 |
| TyG-BMI | 148.896 | 0.264 | 0.181 | 0.386 |
| TyG-BMI | 149.656 | 0.269 | 0.187 | 0.389 |
| TyG-BMI | 150.416 | 0.275 | 0.192 | 0.393 |
| TyG-BMI | 151.177 | 0.280 | 0.198 | 0.397 |
| TyG-BMI | 151.937 | 0.286 | 0.204 | 0.401 |
| TyG-BMI | 152.697 | 0.292 | 0.210 | 0.406 |
| TyG-BMI | 153.458 | 0.299 | 0.216 | 0.412 |
| TyG-BMI | 154.218 | 0.305 | 0.223 | 0.418 |
| TyG-BMI | 154.978 | 0.312 | 0.229 | 0.425 |
| TyG-BMI | 155.738 | 0.319 | 0.235 | 0.433 |
| TyG-BMI | 156.499 | 0.326 | 0.242 | 0.441 |
| TyG-BMI | 157.259 | 0.334 | 0.248 | 0.449 |
| TyG-BMI | 158.019 | 0.342 | 0.255 | 0.458 |
| TyG-BMI | 158.780 | 0.350 | 0.262 | 0.468 |
| TyG-BMI | 159.540 | 0.359 | 0.269 | 0.478 |
| TyG-BMI | 160.300 | 0.368 | 0.276 | 0.489 |
| TyG-BMI | 161.061 | 0.377 | 0.284 | 0.500 |
| TyG-BMI | 161.821 | 0.387 | 0.292 | 0.512 |
| TyG-BMI | 162.581 | 0.397 | 0.300 | 0.525 |
| TyG-BMI | 163.342 | 0.408 | 0.309 | 0.537 |
| TyG-BMI | 164.102 | 0.419 | 0.318 | 0.551 |
| TyG-BMI | 164.862 | 0.430 | 0.328 | 0.564 |
| TyG-BMI | 165.623 | 0.443 | 0.339 | 0.579 |
| TyG-BMI | 166.383 | 0.455 | 0.350 | 0.593 |
| TyG-BMI | 167.143 | 0.469 | 0.362 | 0.608 |
| TyG-BMI | 167.904 | 0.483 | 0.374 | 0.624 |
| TyG-BMI | 168.664 | 0.498 | 0.388 | 0.640 |
| TyG-BMI | 169.424 | 0.513 | 0.402 | 0.656 |
| TyG-BMI | 170.185 | 0.530 | 0.417 | 0.673 |
| TyG-BMI | 170.945 | 0.547 | 0.433 | 0.690 |
| TyG-BMI | 171.705 | 0.564 | 0.450 | 0.708 |
| TyG-BMI | 172.466 | 0.583 | 0.468 | 0.726 |
| TyG-BMI | 173.226 | 0.602 | 0.486 | 0.745 |
| TyG-BMI | 173.986 | 0.622 | 0.505 | 0.765 |
| TyG-BMI | 174.746 | 0.642 | 0.525 | 0.785 |
| TyG-BMI | 175.507 | 0.664 | 0.546 | 0.806 |
| TyG-BMI | 176.267 | 0.685 | 0.568 | 0.828 |
| TyG-BMI | 177.027 | 0.708 | 0.589 | 0.851 |
| TyG-BMI | 177.788 | 0.731 | 0.612 | 0.874 |
| TyG-BMI | 178.548 | 0.755 | 0.634 | 0.899 |
| TyG-BMI | 179.308 | 0.779 | 0.657 | 0.924 |
| TyG-BMI | 180.069 | 0.804 | 0.680 | 0.951 |
| TyG-BMI | 180.829 | 0.829 | 0.703 | 0.979 |
| TyG-BMI | 181.589 | 0.855 | 0.726 | 1.007 |
| TyG-BMI | 182.350 | 0.881 | 0.749 | 1.037 |
| TyG-BMI | 183.110 | 0.907 | 0.771 | 1.067 |
| TyG-BMI | 183.870 | 0.934 | 0.794 | 1.098 |
| TyG-BMI | 184.631 | 0.960 | 0.816 | 1.130 |
| TyG-BMI | 185.391 | 0.987 | 0.838 | 1.162 |
| TyG-BMI | 186.151 | 1.013 | 0.859 | 1.194 |
| TyG-BMI | 186.912 | 1.039 | 0.881 | 1.226 |
| TyG-BMI | 187.672 | 1.065 | 0.902 | 1.258 |
| TyG-BMI | 188.432 | 1.091 | 0.923 | 1.289 |
| TyG-BMI | 189.193 | 1.117 | 0.945 | 1.320 |
| TyG-BMI | 189.953 | 1.142 | 0.966 | 1.350 |
| TyG-BMI | 190.713 | 1.167 | 0.986 | 1.380 |
| TyG-BMI | 191.474 | 1.192 | 1.007 | 1.410 |
| TyG-BMI | 192.234 | 1.216 | 1.028 | 1.439 |
| TyG-BMI | 192.994 | 1.240 | 1.049 | 1.467 |
| TyG-BMI | 193.754 | 1.264 | 1.069 | 1.495 |
| TyG-BMI | 194.515 | 1.288 | 1.089 | 1.522 |
| TyG-BMI | 195.275 | 1.311 | 1.110 | 1.549 |
| TyG-BMI | 196.035 | 1.334 | 1.130 | 1.575 |
| TyG-BMI | 196.796 | 1.356 | 1.149 | 1.601 |
| TyG-BMI | 197.556 | 1.379 | 1.169 | 1.626 |
| TyG-BMI | 198.316 | 1.401 | 1.188 | 1.651 |
| TyG-BMI | 199.077 | 1.422 | 1.207 | 1.676 |
| TyG-BMI | 199.837 | 1.444 | 1.226 | 1.701 |
| TyG-BMI | 200.597 | 1.465 | 1.244 | 1.726 |
| TyG-BMI | 201.358 | 1.486 | 1.262 | 1.751 |
| TyG-BMI | 202.118 | 1.507 | 1.280 | 1.775 |
| TyG-BMI | 202.878 | 1.528 | 1.297 | 1.800 |
| TyG-BMI | 203.639 | 1.548 | 1.313 | 1.825 |
| TyG-BMI | 204.399 | 1.569 | 1.330 | 1.851 |
| TyG-BMI | 205.159 | 1.589 | 1.345 | 1.877 |
| TyG-BMI | 205.920 | 1.609 | 1.361 | 1.903 |
| TyG-BMI | 206.680 | 1.629 | 1.376 | 1.929 |
| TyG-BMI | 207.440 | 1.649 | 1.391 | 1.956 |
| TyG-BMI | 208.201 | 1.670 | 1.405 | 1.984 |
| TyG-BMI | 208.961 | 1.690 | 1.419 | 2.012 |
| TyG-BMI | 209.721 | 1.710 | 1.433 | 2.040 |
| TyG-BMI | 210.482 | 1.730 | 1.447 | 2.069 |
| TyG-BMI | 211.242 | 1.750 | 1.460 | 2.098 |
| TyG-BMI | 212.002 | 1.770 | 1.473 | 2.127 |
| TyG-BMI | 212.762 | 1.790 | 1.486 | 2.157 |
| TyG-BMI | 213.523 | 1.810 | 1.498 | 2.187 |
| TyG-BMI | 214.283 | 1.830 | 1.510 | 2.218 |
| TyG-BMI | 215.043 | 1.850 | 1.522 | 2.248 |
| TyG-BMI | 215.804 | 1.870 | 1.534 | 2.279 |
| TyG-BMI | 216.564 | 1.890 | 1.545 | 2.310 |
| TyG-BMI | 217.324 | 1.909 | 1.557 | 2.342 |
| TyG-BMI | 218.085 | 1.929 | 1.568 | 2.373 |
| TyG-BMI | 218.845 | 1.949 | 1.579 | 2.405 |
| TyG-BMI | 219.605 | 1.968 | 1.589 | 2.437 |
| TyG-BMI | 220.366 | 1.988 | 1.600 | 2.470 |
| TyG-BMI | 221.126 | 2.007 | 1.610 | 2.502 |
| TyG-BMI | 221.886 | 2.026 | 1.620 | 2.534 |
| TyG-BMI | 222.647 | 2.046 | 1.630 | 2.567 |
| TyG-BMI | 223.407 | 2.065 | 1.640 | 2.600 |
| TyG-BMI | 224.167 | 2.084 | 1.649 | 2.633 |
| TyG-BMI | 224.928 | 2.103 | 1.659 | 2.666 |
| TyG-BMI | 225.688 | 2.122 | 1.668 | 2.699 |
| TyG-BMI | 226.448 | 2.140 | 1.677 | 2.732 |
| TyG-BMI | 227.209 | 2.159 | 1.685 | 2.765 |
| TyG-BMI | 227.969 | 2.177 | 1.694 | 2.799 |
| TyG-BMI | 228.729 | 2.196 | 1.702 | 2.832 |
| TyG-BMI | 229.490 | 2.214 | 1.711 | 2.866 |
| TyG-BMI | 230.250 | 2.232 | 1.719 | 2.899 |
| TyG-BMI | 231.010 | 2.250 | 1.726 | 2.933 |
| TyG-BMI | 231.770 | 2.268 | 1.734 | 2.967 |
| TyG-BMI | 232.531 | 2.286 | 1.741 | 3.001 |
| TyG-BMI | 233.291 | 2.304 | 1.749 | 3.035 |
| TyG-BMI | 234.051 | 2.321 | 1.756 | 3.069 |
| TyG-BMI | 234.812 | 2.338 | 1.762 | 3.103 |
| TyG-BMI | 235.572 | 2.356 | 1.769 | 3.137 |
| TyG-BMI | 236.332 | 2.373 | 1.775 | 3.171 |
| TyG-BMI | 237.093 | 2.390 | 1.782 | 3.205 |
| TyG-BMI | 237.853 | 2.406 | 1.788 | 3.240 |
| TyG-BMI | 238.613 | 2.423 | 1.793 | 3.274 |
| TyG-BMI | 239.374 | 2.440 | 1.799 | 3.308 |
| TyG-BMI | 240.134 | 2.456 | 1.804 | 3.343 |
| TyG-BMI | 240.894 | 2.472 | 1.809 | 3.378 |
| TyG-BMI | 241.655 | 2.488 | 1.814 | 3.412 |
| TyG-BMI | 242.415 | 2.504 | 1.819 | 3.447 |
| TyG-BMI | 243.175 | 2.519 | 1.823 | 3.482 |
| TyG-BMI | 243.936 | 2.535 | 1.827 | 3.517 |
| TyG-BMI | 244.696 | 2.550 | 1.831 | 3.552 |
| TyG-BMI | 245.456 | 2.565 | 1.834 | 3.587 |
| TyG-BMI | 246.217 | 2.580 | 1.838 | 3.623 |
| TyG-BMI | 246.977 | 2.595 | 1.841 | 3.658 |
| TyG-BMI | 247.737 | 2.610 | 1.844 | 3.694 |
| TyG-BMI | 248.498 | 2.624 | 1.847 | 3.729 |
| TyG-BMI | 249.258 | 2.638 | 1.849 | 3.765 |
| TyG-BMI | 250.018 | 2.653 | 1.851 | 3.801 |
| TyG-BMI | 250.778 | 2.666 | 1.853 | 3.837 |
| TyG-BMI | 251.539 | 2.680 | 1.855 | 3.873 |
| TyG-BMI | 252.299 | 2.694 | 1.856 | 3.910 |
| TyG-BMI | 253.059 | 2.707 | 1.857 | 3.946 |
| TyG-BMI | 253.820 | 2.720 | 1.858 | 3.983 |
| TyG-BMI | 254.580 | 2.733 | 1.858 | 4.020 |
| TyG-BMI | 255.340 | 2.746 | 1.859 | 4.057 |
| TyG-BMI | 256.101 | 2.759 | 1.859 | 4.094 |
| TyG-BMI | 256.861 | 2.771 | 1.859 | 4.131 |
| TyG-BMI | 257.621 | 2.783 | 1.858 | 4.169 |
| TyG-BMI | 258.382 | 2.795 | 1.857 | 4.207 |
| TyG-BMI | 259.142 | 2.807 | 1.856 | 4.245 |
| TyG-BMI | 259.902 | 2.819 | 1.855 | 4.283 |
| TyG-BMI | 260.663 | 2.830 | 1.854 | 4.322 |
| TyG-BMI | 261.423 | 2.842 | 1.852 | 4.361 |
| TyG-BMI | 262.183 | 2.853 | 1.850 | 4.400 |
| TyG-BMI | 262.944 | 2.864 | 1.848 | 4.439 |
| TyG-BMI | 263.704 | 2.875 | 1.845 | 4.478 |
| TyG-BMI | 264.464 | 2.885 | 1.842 | 4.518 |
| TyG-BMI | 265.225 | 2.896 | 1.839 | 4.558 |
| TyG-BMI | 265.985 | 2.906 | 1.836 | 4.599 |
| TyG-BMI | 266.745 | 2.916 | 1.833 | 4.639 |
| TyG-BMI | 267.506 | 2.926 | 1.829 | 4.680 |
| TyG-BMI | 268.266 | 2.935 | 1.825 | 4.721 |
| TyG-BMI | 269.026 | 2.945 | 1.821 | 4.763 |
| TyG-BMI | 269.786 | 2.954 | 1.816 | 4.805 |
| TyG-BMI | 270.547 | 2.963 | 1.812 | 4.847 |
| TyG-BMI | 271.307 | 2.972 | 1.807 | 4.890 |
| TyG-BMI | 272.067 | 2.981 | 1.802 | 4.932 |
| TyG-BMI | 272.828 | 2.990 | 1.796 | 4.976 |
| TyG-BMI | 273.588 | 2.998 | 1.791 | 5.019 |
| TyG-BMI | 274.348 | 3.006 | 1.785 | 5.063 |
| TyG-BMI | 275.109 | 3.014 | 1.779 | 5.108 |
| TyG-BMI | 275.869 | 3.022 | 1.773 | 5.152 |
| TyG-BMI | 276.629 | 3.030 | 1.766 | 5.197 |
| TyG-BMI | 277.390 | 3.038 | 1.760 | 5.243 |
| TyG-BMI | 278.150 | 3.045 | 1.753 | 5.289 |
| TyG-BMI | 278.910 | 3.052 | 1.746 | 5.335 |
| TyG-BMI | 279.671 | 3.059 | 1.739 | 5.382 |
| TyG-BMI | 280.431 | 3.066 | 1.732 | 5.429 |
| TyG-BMI | 281.191 | 3.073 | 1.724 | 5.477 |
| TyG-BMI | 281.952 | 3.080 | 1.717 | 5.525 |
| TyG-BMI | 282.712 | 3.086 | 1.709 | 5.573 |
| TyG-BMI | 283.472 | 3.093 | 1.701 | 5.622 |
| eGDR | 5.541 | 2.710 | 1.414 | 5.194 |
| eGDR | 5.577 | 2.701 | 1.416 | 5.151 |
| eGDR | 5.613 | 2.692 | 1.418 | 5.109 |
| eGDR | 5.648 | 2.683 | 1.420 | 5.067 |
| eGDR | 5.684 | 2.674 | 1.422 | 5.025 |
| eGDR | 5.719 | 2.664 | 1.424 | 4.984 |
| eGDR | 5.755 | 2.655 | 1.426 | 4.943 |
| eGDR | 5.791 | 2.646 | 1.428 | 4.903 |
| eGDR | 5.826 | 2.637 | 1.430 | 4.864 |
| eGDR | 5.862 | 2.628 | 1.432 | 4.824 |
| eGDR | 5.897 | 2.619 | 1.433 | 4.785 |
| eGDR | 5.933 | 2.610 | 1.435 | 4.747 |
| eGDR | 5.969 | 2.601 | 1.436 | 4.709 |
| eGDR | 6.004 | 2.592 | 1.438 | 4.671 |
| eGDR | 6.040 | 2.583 | 1.439 | 4.634 |
| eGDR | 6.075 | 2.574 | 1.441 | 4.597 |
| eGDR | 6.111 | 2.564 | 1.442 | 4.561 |
| eGDR | 6.146 | 2.555 | 1.443 | 4.525 |
| eGDR | 6.182 | 2.546 | 1.444 | 4.489 |
| eGDR | 6.218 | 2.537 | 1.445 | 4.454 |
| eGDR | 6.253 | 2.528 | 1.446 | 4.419 |
| eGDR | 6.289 | 2.519 | 1.447 | 4.385 |
| eGDR | 6.324 | 2.510 | 1.448 | 4.350 |
| eGDR | 6.360 | 2.501 | 1.449 | 4.317 |
| eGDR | 6.396 | 2.492 | 1.449 | 4.283 |
| eGDR | 6.431 | 2.482 | 1.450 | 4.250 |
| eGDR | 6.467 | 2.473 | 1.451 | 4.217 |
| eGDR | 6.502 | 2.464 | 1.451 | 4.185 |
| eGDR | 6.538 | 2.455 | 1.451 | 4.152 |
| eGDR | 6.574 | 2.446 | 1.452 | 4.120 |
| eGDR | 6.609 | 2.437 | 1.452 | 4.089 |
| eGDR | 6.645 | 2.427 | 1.452 | 4.057 |
| eGDR | 6.680 | 2.418 | 1.452 | 4.026 |
| eGDR | 6.716 | 2.409 | 1.453 | 3.996 |
| eGDR | 6.752 | 2.400 | 1.453 | 3.965 |
| eGDR | 6.787 | 2.391 | 1.452 | 3.935 |
| eGDR | 6.823 | 2.381 | 1.452 | 3.905 |
| eGDR | 6.858 | 2.372 | 1.452 | 3.875 |
| eGDR | 6.894 | 2.363 | 1.452 | 3.846 |
| eGDR | 6.929 | 2.354 | 1.452 | 3.816 |
| eGDR | 6.965 | 2.344 | 1.451 | 3.787 |
| eGDR | 7.001 | 2.335 | 1.451 | 3.759 |
| eGDR | 7.036 | 2.326 | 1.450 | 3.730 |
| eGDR | 7.072 | 2.317 | 1.450 | 3.702 |
| eGDR | 7.107 | 2.307 | 1.449 | 3.674 |
| eGDR | 7.143 | 2.298 | 1.448 | 3.646 |
| eGDR | 7.179 | 2.289 | 1.448 | 3.618 |
| eGDR | 7.214 | 2.279 | 1.447 | 3.590 |
| eGDR | 7.250 | 2.270 | 1.446 | 3.563 |
| eGDR | 7.285 | 2.260 | 1.445 | 3.536 |
| eGDR | 7.321 | 2.251 | 1.444 | 3.509 |
| eGDR | 7.357 | 2.242 | 1.443 | 3.482 |
| eGDR | 7.392 | 2.232 | 1.442 | 3.455 |
| eGDR | 7.428 | 2.223 | 1.441 | 3.429 |
| eGDR | 7.463 | 2.213 | 1.440 | 3.402 |
| eGDR | 7.499 | 2.204 | 1.439 | 3.376 |
| eGDR | 7.535 | 2.194 | 1.437 | 3.350 |
| eGDR | 7.570 | 2.185 | 1.436 | 3.324 |
| eGDR | 7.606 | 2.175 | 1.435 | 3.298 |
| eGDR | 7.641 | 2.166 | 1.433 | 3.272 |
| eGDR | 7.677 | 2.156 | 1.432 | 3.247 |
| eGDR | 7.712 | 2.147 | 1.430 | 3.221 |
| eGDR | 7.748 | 2.137 | 1.429 | 3.196 |
| eGDR | 7.784 | 2.127 | 1.427 | 3.171 |
| eGDR | 7.819 | 2.118 | 1.426 | 3.145 |
| eGDR | 7.855 | 2.108 | 1.424 | 3.120 |
| eGDR | 7.890 | 2.099 | 1.423 | 3.095 |
| eGDR | 7.926 | 2.089 | 1.421 | 3.071 |
| eGDR | 7.962 | 2.079 | 1.419 | 3.046 |
| eGDR | 7.997 | 2.070 | 1.418 | 3.021 |
| eGDR | 8.033 | 2.060 | 1.416 | 2.996 |
| eGDR | 8.068 | 2.050 | 1.414 | 2.972 |
| eGDR | 8.104 | 2.040 | 1.412 | 2.947 |
| eGDR | 8.140 | 2.031 | 1.411 | 2.923 |
| eGDR | 8.175 | 2.021 | 1.409 | 2.899 |
| eGDR | 8.211 | 2.011 | 1.407 | 2.874 |
| eGDR | 8.246 | 2.001 | 1.405 | 2.850 |
| eGDR | 8.282 | 1.991 | 1.403 | 2.826 |
| eGDR | 8.317 | 1.982 | 1.401 | 2.802 |
| eGDR | 8.353 | 1.972 | 1.400 | 2.778 |
| eGDR | 8.389 | 1.962 | 1.398 | 2.754 |
| eGDR | 8.424 | 1.952 | 1.396 | 2.730 |
| eGDR | 8.460 | 1.942 | 1.394 | 2.706 |
| eGDR | 8.495 | 1.932 | 1.392 | 2.683 |
| eGDR | 8.531 | 1.922 | 1.390 | 2.659 |
| eGDR | 8.567 | 1.912 | 1.388 | 2.635 |
| eGDR | 8.602 | 1.903 | 1.386 | 2.612 |
| eGDR | 8.638 | 1.893 | 1.384 | 2.588 |
| eGDR | 8.673 | 1.883 | 1.382 | 2.565 |
| eGDR | 8.709 | 1.873 | 1.380 | 2.541 |
| eGDR | 8.745 | 1.863 | 1.378 | 2.518 |
| eGDR | 8.780 | 1.853 | 1.376 | 2.495 |
| eGDR | 8.816 | 1.843 | 1.374 | 2.472 |
| eGDR | 8.851 | 1.833 | 1.372 | 2.448 |
| eGDR | 8.887 | 1.823 | 1.370 | 2.425 |
| eGDR | 8.923 | 1.813 | 1.368 | 2.402 |
| eGDR | 8.958 | 1.802 | 1.365 | 2.379 |
| eGDR | 8.994 | 1.792 | 1.363 | 2.357 |
| eGDR | 9.029 | 1.782 | 1.361 | 2.334 |
| eGDR | 9.065 | 1.772 | 1.359 | 2.311 |
| eGDR | 9.100 | 1.762 | 1.357 | 2.289 |
| eGDR | 9.136 | 1.752 | 1.354 | 2.266 |
| eGDR | 9.172 | 1.742 | 1.352 | 2.244 |
| eGDR | 9.207 | 1.732 | 1.350 | 2.222 |
| eGDR | 9.243 | 1.722 | 1.347 | 2.200 |
| eGDR | 9.278 | 1.711 | 1.345 | 2.178 |
| eGDR | 9.314 | 1.701 | 1.342 | 2.156 |
| eGDR | 9.350 | 1.691 | 1.340 | 2.135 |
| eGDR | 9.385 | 1.681 | 1.337 | 2.114 |
| eGDR | 9.421 | 1.671 | 1.334 | 2.092 |
| eGDR | 9.456 | 1.661 | 1.331 | 2.071 |
| eGDR | 9.492 | 1.650 | 1.328 | 2.051 |
| eGDR | 9.528 | 1.640 | 1.325 | 2.030 |
| eGDR | 9.563 | 1.630 | 1.322 | 2.010 |
| eGDR | 9.599 | 1.620 | 1.318 | 1.990 |
| eGDR | 9.634 | 1.610 | 1.315 | 1.970 |
| eGDR | 9.670 | 1.599 | 1.311 | 1.951 |
| eGDR | 9.706 | 1.589 | 1.307 | 1.932 |
| eGDR | 9.741 | 1.579 | 1.303 | 1.913 |
| eGDR | 9.777 | 1.569 | 1.298 | 1.895 |
| eGDR | 9.812 | 1.558 | 1.294 | 1.877 |
| eGDR | 9.848 | 1.548 | 1.289 | 1.860 |
| eGDR | 9.883 | 1.538 | 1.283 | 1.843 |
| eGDR | 9.919 | 1.528 | 1.278 | 1.826 |
| eGDR | 9.955 | 1.517 | 1.272 | 1.810 |
| eGDR | 9.990 | 1.507 | 1.265 | 1.795 |
| eGDR | 10.026 | 1.496 | 1.258 | 1.780 |
| eGDR | 10.061 | 1.485 | 1.250 | 1.765 |
| eGDR | 10.097 | 1.474 | 1.242 | 1.749 |
| eGDR | 10.133 | 1.462 | 1.233 | 1.734 |
| eGDR | 10.168 | 1.450 | 1.223 | 1.719 |
| eGDR | 10.204 | 1.437 | 1.212 | 1.703 |
| eGDR | 10.239 | 1.423 | 1.201 | 1.687 |
| eGDR | 10.275 | 1.409 | 1.189 | 1.670 |
| eGDR | 10.311 | 1.393 | 1.176 | 1.652 |
| eGDR | 10.346 | 1.377 | 1.162 | 1.633 |
| eGDR | 10.382 | 1.360 | 1.147 | 1.613 |
| eGDR | 10.417 | 1.342 | 1.132 | 1.592 |
| eGDR | 10.453 | 1.323 | 1.116 | 1.569 |
| eGDR | 10.488 | 1.303 | 1.099 | 1.545 |
| eGDR | 10.524 | 1.282 | 1.081 | 1.519 |
| eGDR | 10.560 | 1.259 | 1.063 | 1.492 |
| eGDR | 10.595 | 1.236 | 1.044 | 1.463 |
| eGDR | 10.631 | 1.212 | 1.024 | 1.433 |
| eGDR | 10.666 | 1.186 | 1.004 | 1.401 |
| eGDR | 10.702 | 1.159 | 0.983 | 1.367 |
| eGDR | 10.738 | 1.132 | 0.961 | 1.332 |
| eGDR | 10.773 | 1.103 | 0.939 | 1.296 |
| eGDR | 10.809 | 1.073 | 0.915 | 1.259 |
| eGDR | 10.844 | 1.043 | 0.891 | 1.221 |
| eGDR | 10.880 | 1.011 | 0.865 | 1.182 |
| eGDR | 10.916 | 0.979 | 0.839 | 1.143 |
| eGDR | 10.951 | 0.946 | 0.812 | 1.103 |
| eGDR | 10.987 | 0.913 | 0.783 | 1.065 |
| eGDR | 11.022 | 0.881 | 0.755 | 1.028 |
| eGDR | 11.058 | 0.848 | 0.725 | 0.992 |
| eGDR | 11.094 | 0.816 | 0.696 | 0.957 |
| eGDR | 11.129 | 0.785 | 0.666 | 0.924 |
| eGDR | 11.165 | 0.754 | 0.637 | 0.893 |
| eGDR | 11.200 | 0.725 | 0.609 | 0.863 |
| eGDR | 11.236 | 0.697 | 0.581 | 0.835 |
| eGDR | 11.271 | 0.670 | 0.555 | 0.808 |
| eGDR | 11.307 | 0.644 | 0.530 | 0.783 |
| eGDR | 11.343 | 0.620 | 0.507 | 0.759 |
| eGDR | 11.378 | 0.597 | 0.485 | 0.735 |
| eGDR | 11.414 | 0.576 | 0.465 | 0.713 |
| eGDR | 11.449 | 0.556 | 0.447 | 0.692 |
| eGDR | 11.485 | 0.538 | 0.431 | 0.672 |
| eGDR | 11.521 | 0.521 | 0.416 | 0.653 |
| eGDR | 11.556 | 0.506 | 0.402 | 0.635 |
| eGDR | 11.592 | 0.491 | 0.390 | 0.618 |
| eGDR | 11.627 | 0.478 | 0.380 | 0.602 |
| eGDR | 11.663 | 0.466 | 0.370 | 0.587 |
| eGDR | 11.699 | 0.455 | 0.361 | 0.573 |
| eGDR | 11.734 | 0.444 | 0.352 | 0.561 |
| eGDR | 11.770 | 0.435 | 0.344 | 0.549 |
| eGDR | 11.805 | 0.426 | 0.336 | 0.539 |
| eGDR | 11.841 | 0.418 | 0.329 | 0.531 |
| eGDR | 11.877 | 0.410 | 0.322 | 0.523 |
| eGDR | 11.912 | 0.403 | 0.314 | 0.518 |
| eGDR | 11.948 | 0.397 | 0.307 | 0.513 |
| eGDR | 11.983 | 0.391 | 0.300 | 0.510 |
| eGDR | 12.019 | 0.386 | 0.292 | 0.509 |
| eGDR | 12.054 | 0.381 | 0.284 | 0.509 |
| eGDR | 12.090 | 0.376 | 0.277 | 0.511 |
| eGDR | 12.126 | 0.372 | 0.269 | 0.513 |
| eGDR | 12.161 | 0.367 | 0.261 | 0.518 |
| eGDR | 12.197 | 0.364 | 0.253 | 0.523 |
| eGDR | 12.232 | 0.360 | 0.245 | 0.530 |
| eGDR | 12.268 | 0.357 | 0.237 | 0.538 |
| eGDR | 12.304 | 0.354 | 0.229 | 0.547 |
| eGDR | 12.339 | 0.351 | 0.222 | 0.557 |
| eGDR | 12.375 | 0.349 | 0.214 | 0.568 |
| eGDR | 12.410 | 0.346 | 0.206 | 0.580 |
| eGDR | 12.446 | 0.344 | 0.199 | 0.593 |
| eGDR | 12.482 | 0.341 | 0.192 | 0.607 |
| eGDR | 12.517 | 0.339 | 0.185 | 0.621 |
| eGDR | 12.553 | 0.337 | 0.178 | 0.637 |
| eGDR | 12.588 | 0.335 | 0.172 | 0.653 |
| eGDR | 12.624 | 0.333 | 0.165 | 0.670 |
| METS-IR | 1.880 | 0.283 | 0.141 | 0.569 |
| METS-IR | 1.884 | 0.289 | 0.147 | 0.568 |
| METS-IR | 1.889 | 0.294 | 0.153 | 0.567 |
| METS-IR | 1.893 | 0.300 | 0.159 | 0.566 |
| METS-IR | 1.897 | 0.306 | 0.166 | 0.565 |
| METS-IR | 1.902 | 0.312 | 0.173 | 0.564 |
| METS-IR | 1.906 | 0.318 | 0.180 | 0.563 |
| METS-IR | 1.910 | 0.325 | 0.187 | 0.563 |
| METS-IR | 1.915 | 0.331 | 0.195 | 0.563 |
| METS-IR | 1.919 | 0.337 | 0.202 | 0.563 |
| METS-IR | 1.923 | 0.344 | 0.210 | 0.563 |
| METS-IR | 1.928 | 0.351 | 0.219 | 0.563 |
| METS-IR | 1.932 | 0.358 | 0.227 | 0.564 |
| METS-IR | 1.936 | 0.365 | 0.236 | 0.564 |
| METS-IR | 1.941 | 0.372 | 0.245 | 0.565 |
| METS-IR | 1.945 | 0.379 | 0.254 | 0.567 |
| METS-IR | 1.949 | 0.387 | 0.263 | 0.569 |
| METS-IR | 1.954 | 0.394 | 0.272 | 0.571 |
| METS-IR | 1.958 | 0.402 | 0.282 | 0.573 |
| METS-IR | 1.962 | 0.409 | 0.291 | 0.576 |
| METS-IR | 1.967 | 0.417 | 0.301 | 0.579 |
| METS-IR | 1.971 | 0.425 | 0.310 | 0.583 |
| METS-IR | 1.975 | 0.434 | 0.320 | 0.587 |
| METS-IR | 1.980 | 0.442 | 0.330 | 0.592 |
| METS-IR | 1.984 | 0.450 | 0.339 | 0.598 |
| METS-IR | 1.988 | 0.459 | 0.349 | 0.603 |
| METS-IR | 1.993 | 0.468 | 0.359 | 0.610 |
| METS-IR | 1.997 | 0.476 | 0.368 | 0.617 |
| METS-IR | 2.001 | 0.485 | 0.377 | 0.625 |
| METS-IR | 2.006 | 0.495 | 0.386 | 0.633 |
| METS-IR | 2.010 | 0.504 | 0.396 | 0.642 |
| METS-IR | 2.014 | 0.513 | 0.404 | 0.651 |
| METS-IR | 2.019 | 0.523 | 0.413 | 0.661 |
| METS-IR | 2.023 | 0.532 | 0.422 | 0.672 |
| METS-IR | 2.027 | 0.542 | 0.430 | 0.683 |
| METS-IR | 2.032 | 0.552 | 0.439 | 0.694 |
| METS-IR | 2.036 | 0.562 | 0.447 | 0.706 |
| METS-IR | 2.040 | 0.572 | 0.456 | 0.718 |
| METS-IR | 2.045 | 0.583 | 0.465 | 0.731 |
| METS-IR | 2.049 | 0.593 | 0.473 | 0.744 |
| METS-IR | 2.053 | 0.604 | 0.482 | 0.756 |
| METS-IR | 2.058 | 0.614 | 0.491 | 0.769 |
| METS-IR | 2.062 | 0.625 | 0.500 | 0.782 |
| METS-IR | 2.066 | 0.636 | 0.509 | 0.795 |
| METS-IR | 2.071 | 0.647 | 0.518 | 0.808 |
| METS-IR | 2.075 | 0.659 | 0.528 | 0.821 |
| METS-IR | 2.079 | 0.670 | 0.538 | 0.833 |
| METS-IR | 2.084 | 0.681 | 0.549 | 0.846 |
| METS-IR | 2.088 | 0.693 | 0.560 | 0.858 |
| METS-IR | 2.092 | 0.705 | 0.571 | 0.869 |
| METS-IR | 2.097 | 0.716 | 0.583 | 0.881 |
| METS-IR | 2.101 | 0.728 | 0.595 | 0.892 |
| METS-IR | 2.105 | 0.740 | 0.607 | 0.903 |
| METS-IR | 2.110 | 0.752 | 0.620 | 0.914 |
| METS-IR | 2.114 | 0.765 | 0.632 | 0.924 |
| METS-IR | 2.118 | 0.777 | 0.645 | 0.935 |
| METS-IR | 2.123 | 0.789 | 0.659 | 0.946 |
| METS-IR | 2.127 | 0.802 | 0.672 | 0.957 |
| METS-IR | 2.132 | 0.814 | 0.685 | 0.968 |
| METS-IR | 2.136 | 0.827 | 0.699 | 0.979 |
| METS-IR | 2.140 | 0.840 | 0.712 | 0.991 |
| METS-IR | 2.145 | 0.852 | 0.725 | 1.003 |
| METS-IR | 2.149 | 0.865 | 0.737 | 1.016 |
| METS-IR | 2.153 | 0.878 | 0.750 | 1.029 |
| METS-IR | 2.158 | 0.891 | 0.762 | 1.043 |
| METS-IR | 2.162 | 0.904 | 0.773 | 1.057 |
| METS-IR | 2.166 | 0.917 | 0.785 | 1.072 |
| METS-IR | 2.171 | 0.930 | 0.796 | 1.088 |
| METS-IR | 2.175 | 0.944 | 0.806 | 1.104 |
| METS-IR | 2.179 | 0.957 | 0.817 | 1.121 |
| METS-IR | 2.184 | 0.970 | 0.827 | 1.137 |
| METS-IR | 2.188 | 0.983 | 0.837 | 1.155 |
| METS-IR | 2.192 | 0.996 | 0.847 | 1.172 |
| METS-IR | 2.197 | 1.009 | 0.857 | 1.189 |
| METS-IR | 2.201 | 1.023 | 0.867 | 1.206 |
| METS-IR | 2.205 | 1.036 | 0.878 | 1.223 |
| METS-IR | 2.210 | 1.049 | 0.888 | 1.239 |
| METS-IR | 2.214 | 1.062 | 0.899 | 1.255 |
| METS-IR | 2.218 | 1.075 | 0.909 | 1.272 |
| METS-IR | 2.223 | 1.088 | 0.920 | 1.287 |
| METS-IR | 2.227 | 1.101 | 0.931 | 1.303 |
| METS-IR | 2.231 | 1.115 | 0.943 | 1.318 |
| METS-IR | 2.236 | 1.128 | 0.954 | 1.332 |
| METS-IR | 2.240 | 1.141 | 0.966 | 1.347 |
| METS-IR | 2.244 | 1.153 | 0.978 | 1.361 |
| METS-IR | 2.249 | 1.166 | 0.989 | 1.375 |
| METS-IR | 2.253 | 1.179 | 1.001 | 1.388 |
| METS-IR | 2.257 | 1.192 | 1.013 | 1.402 |
| METS-IR | 2.262 | 1.205 | 1.026 | 1.415 |
| METS-IR | 2.266 | 1.217 | 1.038 | 1.428 |
| METS-IR | 2.270 | 1.230 | 1.050 | 1.441 |
| METS-IR | 2.275 | 1.243 | 1.062 | 1.454 |
| METS-IR | 2.279 | 1.255 | 1.074 | 1.467 |
| METS-IR | 2.283 | 1.268 | 1.085 | 1.480 |
| METS-IR | 2.288 | 1.280 | 1.097 | 1.493 |
| METS-IR | 2.292 | 1.292 | 1.109 | 1.506 |
| METS-IR | 2.296 | 1.304 | 1.120 | 1.519 |
| METS-IR | 2.301 | 1.317 | 1.131 | 1.533 |
| METS-IR | 2.305 | 1.329 | 1.142 | 1.546 |
| METS-IR | 2.309 | 1.341 | 1.152 | 1.560 |
| METS-IR | 2.314 | 1.352 | 1.163 | 1.573 |
| METS-IR | 2.318 | 1.364 | 1.173 | 1.587 |
| METS-IR | 2.322 | 1.376 | 1.183 | 1.601 |
| METS-IR | 2.327 | 1.388 | 1.192 | 1.615 |
| METS-IR | 2.331 | 1.399 | 1.202 | 1.629 |
| METS-IR | 2.335 | 1.410 | 1.211 | 1.643 |
| METS-IR | 2.340 | 1.422 | 1.220 | 1.657 |
| METS-IR | 2.344 | 1.433 | 1.229 | 1.671 |
| METS-IR | 2.348 | 1.444 | 1.237 | 1.685 |
| METS-IR | 2.353 | 1.455 | 1.245 | 1.700 |
| METS-IR | 2.357 | 1.466 | 1.254 | 1.714 |
| METS-IR | 2.361 | 1.476 | 1.262 | 1.728 |
| METS-IR | 2.366 | 1.487 | 1.269 | 1.742 |
| METS-IR | 2.370 | 1.498 | 1.277 | 1.756 |
| METS-IR | 2.374 | 1.508 | 1.284 | 1.770 |
| METS-IR | 2.379 | 1.518 | 1.292 | 1.784 |
| METS-IR | 2.383 | 1.528 | 1.299 | 1.798 |
| METS-IR | 2.387 | 1.538 | 1.306 | 1.812 |
| METS-IR | 2.392 | 1.548 | 1.312 | 1.826 |
| METS-IR | 2.396 | 1.558 | 1.319 | 1.839 |
| METS-IR | 2.400 | 1.567 | 1.325 | 1.853 |
| METS-IR | 2.405 | 1.576 | 1.332 | 1.866 |
| METS-IR | 2.409 | 1.586 | 1.338 | 1.879 |
| METS-IR | 2.413 | 1.595 | 1.344 | 1.892 |
| METS-IR | 2.418 | 1.604 | 1.350 | 1.905 |
| METS-IR | 2.422 | 1.612 | 1.356 | 1.918 |
| METS-IR | 2.426 | 1.621 | 1.361 | 1.930 |
| METS-IR | 2.431 | 1.630 | 1.367 | 1.942 |
| METS-IR | 2.435 | 1.638 | 1.372 | 1.955 |
| METS-IR | 2.439 | 1.646 | 1.378 | 1.966 |
| METS-IR | 2.444 | 1.654 | 1.383 | 1.978 |
| METS-IR | 2.448 | 1.662 | 1.388 | 1.990 |
| METS-IR | 2.452 | 1.669 | 1.393 | 2.001 |
| METS-IR | 2.457 | 1.677 | 1.398 | 2.012 |
| METS-IR | 2.461 | 1.684 | 1.402 | 2.023 |
| METS-IR | 2.466 | 1.691 | 1.407 | 2.034 |
| METS-IR | 2.470 | 1.698 | 1.411 | 2.044 |
| METS-IR | 2.474 | 1.705 | 1.415 | 2.054 |
| METS-IR | 2.479 | 1.712 | 1.420 | 2.064 |
| METS-IR | 2.483 | 1.718 | 1.424 | 2.074 |
| METS-IR | 2.487 | 1.725 | 1.428 | 2.084 |
| METS-IR | 2.492 | 1.731 | 1.431 | 2.093 |
| METS-IR | 2.496 | 1.737 | 1.435 | 2.102 |
| METS-IR | 2.500 | 1.742 | 1.438 | 2.111 |
| METS-IR | 2.505 | 1.748 | 1.442 | 2.119 |
| METS-IR | 2.509 | 1.753 | 1.445 | 2.128 |
| METS-IR | 2.513 | 1.759 | 1.448 | 2.136 |
| METS-IR | 2.518 | 1.764 | 1.451 | 2.144 |
| METS-IR | 2.522 | 1.769 | 1.454 | 2.152 |
| METS-IR | 2.526 | 1.773 | 1.457 | 2.159 |
| METS-IR | 2.531 | 1.778 | 1.459 | 2.166 |
| METS-IR | 2.535 | 1.782 | 1.461 | 2.173 |
| METS-IR | 2.539 | 1.786 | 1.464 | 2.180 |
| METS-IR | 2.544 | 1.790 | 1.466 | 2.187 |
| METS-IR | 2.548 | 1.794 | 1.468 | 2.193 |
| METS-IR | 2.552 | 1.798 | 1.469 | 2.200 |
| METS-IR | 2.557 | 1.801 | 1.471 | 2.206 |
| METS-IR | 2.561 | 1.805 | 1.472 | 2.212 |
| METS-IR | 2.565 | 1.808 | 1.474 | 2.217 |
| METS-IR | 2.570 | 1.811 | 1.475 | 2.223 |
| METS-IR | 2.574 | 1.813 | 1.476 | 2.228 |
| METS-IR | 2.578 | 1.816 | 1.476 | 2.234 |
| METS-IR | 2.583 | 1.818 | 1.477 | 2.239 |
| METS-IR | 2.587 | 1.821 | 1.477 | 2.244 |
| METS-IR | 2.591 | 1.823 | 1.477 | 2.248 |
| METS-IR | 2.596 | 1.824 | 1.477 | 2.253 |
| METS-IR | 2.600 | 1.826 | 1.477 | 2.258 |
| METS-IR | 2.604 | 1.828 | 1.477 | 2.262 |
| METS-IR | 2.609 | 1.829 | 1.476 | 2.266 |
| METS-IR | 2.613 | 1.830 | 1.475 | 2.270 |
| METS-IR | 2.617 | 1.831 | 1.474 | 2.274 |
| METS-IR | 2.622 | 1.832 | 1.473 | 2.278 |
| METS-IR | 2.626 | 1.833 | 1.472 | 2.282 |
| METS-IR | 2.630 | 1.833 | 1.470 | 2.286 |
| METS-IR | 2.635 | 1.834 | 1.469 | 2.289 |
| METS-IR | 2.639 | 1.834 | 1.466 | 2.293 |
| METS-IR | 2.643 | 1.834 | 1.464 | 2.297 |
| METS-IR | 2.648 | 1.834 | 1.462 | 2.300 |
| METS-IR | 2.652 | 1.833 | 1.459 | 2.304 |
| METS-IR | 2.656 | 1.833 | 1.456 | 2.307 |
| METS-IR | 2.661 | 1.832 | 1.453 | 2.310 |
| METS-IR | 2.665 | 1.832 | 1.450 | 2.314 |
| METS-IR | 2.669 | 1.831 | 1.446 | 2.317 |
| METS-IR | 2.674 | 1.830 | 1.443 | 2.320 |
| METS-IR | 2.678 | 1.828 | 1.439 | 2.323 |
| METS-IR | 2.682 | 1.827 | 1.435 | 2.326 |
| METS-IR | 2.687 | 1.825 | 1.430 | 2.330 |
| METS-IR | 2.691 | 1.824 | 1.426 | 2.333 |
| METS-IR | 2.695 | 1.822 | 1.421 | 2.336 |
| METS-IR | 2.700 | 1.820 | 1.416 | 2.339 |
| METS-IR | 2.704 | 1.818 | 1.411 | 2.342 |
| METS-IR | 2.708 | 1.816 | 1.405 | 2.346 |
| METS-IR | 2.713 | 1.813 | 1.400 | 2.349 |
| METS-IR | 2.717 | 1.811 | 1.394 | 2.352 |
| METS-IR | 2.721 | 1.808 | 1.388 | 2.355 |
| METS-IR | 2.726 | 1.805 | 1.382 | 2.359 |
| METS-IR | 2.730 | 1.802 | 1.375 | 2.362 |
| METS-IR | 2.734 | 1.799 | 1.369 | 2.365 |
| METS-IR | 2.739 | 1.796 | 1.362 | 2.369 |
| METS-IR | 2.743 | 1.793 | 1.355 | 2.372 |
| *RR, risk ratio; CI, confidence interval. Splines used 4 knots at the 5th, 35th, 65th, and 95th percentiles.* | | | | |
