# Supplementary material for: Non-traditional metabolic indices predict incident circadian syndrome in middle-aged and older Chinese adults: a nationwide prospective cohort study and machine learning analysis
Source: Lipids Health Dis. 2026 May 13;25:167. doi: 10.1186/s12944-026-02972-9 (PMC13339493; doi:10.1186/s12944-026-02972-9)
Supplement: Supplementary file 1 — Supplementary Material 1. [file 12944_2026_2972_MOESM1_ESM.zip › Table_S06.docx]

**Table S6. Spearman rank correlation coefficients among eight metabolic indices**

| **Index** | **AIP** | **CHG Index** | **RCII** | **hs-CRP/HDL-C** | **CTI** | **TyG-BMI** | **eGDR** | **METS-IR** |
| --- | --- | --- | --- | --- | --- | --- | --- | --- |
| AIP | 1.000 | 0.635 | 0.460 | 0.266 | 0.643 | 0.475 | -0.155 | 0.819 |
| CHG Index | 0.635 | 1.000 | 0.339 | 0.280 | 0.522 | 0.423 | -0.237 | 0.722 |
| RCII | 0.460 | 0.339 | 1.000 | 0.803 | 0.822 | 0.277 | -0.181 | 0.375 |
| hs-CRP/HDL-C | 0.266 | 0.280 | 0.803 | 1.000 | 0.717 | 0.191 | -0.186 | 0.370 |
| CTI | 0.643 | 0.522 | 0.822 | 0.717 | 1.000 | 0.411 | -0.224 | 0.480 |
| TyG-BMI | 0.475 | 0.423 | 0.277 | 0.191 | 0.411 | 1.000 | -0.587 | 0.549 |
| eGDR | -0.155 | -0.237 | -0.181 | -0.186 | -0.224 | -0.587 | 1.000 | -0.265 |
| METS-IR | 0.819 | 0.722 | 0.375 | 0.370 | 0.480 | 0.549 | -0.265 | 1.000 |
| *Abbreviations: AIP, atherogenic index of plasma; CHG, cholesterol–HDL–glucose index; CTI, C-reactive protein triglyceride glucose index; eGDR, estimated glucose disposal rate; METS-IR, metabolic score for insulin resistance; RCII, remnant cholesterol inflammation index; TyG-BMI, triglyceride–glucose–body mass index.* | | | | | | | | |
