# Supplementary material for: Non-traditional metabolic indices predict incident circadian syndrome in middle-aged and older Chinese adults: a nationwide prospective cohort study and machine learning analysis
Source: Lipids Health Dis. 2026 May 13;25:167. doi: 10.1186/s12944-026-02972-9 (PMC13339493; doi:10.1186/s12944-026-02972-9)
Supplement: Supplementary file 1 — Supplementary Material 1. [file 12944_2026_2972_MOESM1_ESM.zip › Table_S07.docx]

**Table S7. Mediation of the metabolic index–CircS association by biological age acceleration**

| **Index** | **Index label** | **N** | **Events** | **ACME** | **ACME lower CI** | **ACME upper CI** | **ACME P** | **ADE** | **ADE lower CI** | **ADE upper CI** | **ADE P** | **Total effect** | **Total lower CI** | **Total upper CI** | **Total P** | **Proportion mediated** | **Prop. lower CI** | **Prop. upper CI** | **Prop. P** |
| --- | --- | --- | --- | --- | --- | --- | --- | --- | --- | --- | --- | --- | --- | --- | --- | --- | --- | --- | --- |
| TyG-BMI | TyG-BMI | 3,263 | 703 | <0.001 | <0.001 | <0.001 | <0.001 | <0.001 | <0.001 | <0.001 | <0.001 | <0.001 | <0.001 | <0.001 | <0.001 | 0.039 | 0.018 | 0.072 | <0.001 |
| eGDR | eGDR | 3,263 | 703 | 0.007 | 0.001 | 0.025 | 0.014 | -0.031 | -0.079 | -0.009 | <0.001 | -0.016 | -0.033 | -0.005 | <0.001 | -0.428 | -0.842 | -0.081 | 0.014 |
| METS-IR | METS-IR | 3,263 | 703 | -<0.001 | -<0.001 | -<0.001 | 0.008 | 0.014 | 0.007 | 0.024 | <0.001 | 0.013 | 0.007 | 0.022 | <0.001 | -0.006 | -0.015 | -0.002 | 0.008 |
| CTI | CTI | 3,263 | 703 | <0.001 | <0.001 | 0.001 | <0.001 | 0.004 | 0.002 | 0.007 | <0.001 | 0.004 | 0.002 | 0.008 | <0.001 | 0.066 | 0.033 | 0.123 | <0.001 |
| *ACME, average causal mediation effect; ADE, average direct effect; CI, confidence interval.* | | | | | | | | | | | | | | | | | | | |
