# Supplementary material for: Non-traditional metabolic indices predict incident circadian syndrome in middle-aged and older Chinese adults: a nationwide prospective cohort study and machine learning analysis
Source: Lipids Health Dis. 2026 May 13;25:167. doi: 10.1186/s12944-026-02972-9 (PMC13339493; doi:10.1186/s12944-026-02972-9)
Supplement: Supplementary file 1 — Supplementary Material 1. [file 12944_2026_2972_MOESM1_ESM.zip › Table_S08.docx]

**Table S8. Cox proportional hazards models as sensitivity analysis**

| **Index** | **Index label** | **HR** | **Lower CI** | **Upper CI** | **P value** | **N** | **Events** |
| --- | --- | --- | --- | --- | --- | --- | --- |
| AIP | AIP | 1.418 | 1.326 | 1.517 | <0.001 | 3,356 | 725 |
| CHG Index | CHG Index | 1.446 | 1.344 | 1.555 | <0.001 | 3,004 | 648 |
| RCII | RCII | 1.104 | 1.044 | 1.167 | <0.001 | 3,354 | 725 |
| hs-CRP/HDL-C | hs-CRP/HDL-C | 1.069 | 1.005 | 1.137 | 0.034 | 3,358 | 725 |
| CTI | CTI | 1.304 | 1.215 | 1.399 | <0.001 | 3,353 | 725 |
| TyG-BMI | TyG-BMI | 2.097 | 1.788 | 2.461 | <0.001 | 3,353 | 725 |
| eGDR | eGDR | 0.492 | 0.415 | 0.583 | <0.001 | 3,345 | 729 |
| METS-IR | METS-IR | 1.405 | 1.316 | 1.499 | <0.001 | 3,353 | 725 |
| *HR, hazard ratio; CI, confidence interval.* | | | | | | | |
