# Supplementary material for: Non-traditional metabolic indices predict incident circadian syndrome in middle-aged and older Chinese adults: a nationwide prospective cohort study and machine learning analysis
Source: Lipids Health Dis. 2026 May 13;25:167. doi: 10.1186/s12944-026-02972-9 (PMC13339493; doi:10.1186/s12944-026-02972-9)
Supplement: Supplementary file 1 — Supplementary Material 1. [file 12944_2026_2972_MOESM1_ESM.zip › Table_S09.docx]

**Table S9. Sensitivity analysis with additional covariate adjustment (per-SD associations with incident CircS)**

| **Index** | **N (A)** | **Model A: RR (95% CI)** | **P (A)** | **N (B)** | **Model B: RR (95% CI)** | **P (B)** | **N (C)** | **Model C: RR (95% CI)** | **P (C)** |
| --- | --- | --- | --- | --- | --- | --- | --- | --- | --- |
| AIP | 3,356 | 1.371 (1.298–1.449) | <0.001 | 3,340 | 1.369 (1.296–1.447) | <0.001 | 1,409 | 1.448 (1.317–1.592) | <0.001 |
| CHG Index | 3,004 | 1.393 (1.314–1.477) | <0.001 | 2,990 | 1.389 (1.310–1.473) | <0.001 | 1,267 | 1.333 (1.225–1.450) | <0.001 |
| RCII | 3,354 | 1.096 (1.046–1.148) | <0.001 | 3,338 | 1.094 (1.044–1.147) | <0.001 | 1,409 | 1.086 (1.013–1.165) | 0.020 |
| hs-CRP/HDL-C | 3,358 | 1.067 (1.010–1.127) | 0.021 | 3,342 | 1.066 (1.009–1.126) | 0.022 | 1,411 | 1.057 (0.982–1.137) | 0.140 |
| CTI | 3,353 | 1.263 (1.190–1.341) | <0.001 | 3,337 | 1.257 (1.184–1.335) | <0.001 | 1,408 | 1.276 (1.161–1.401) | <0.001 |
| TyG-BMI | 3,353 | 1.883 (1.671–2.121) | <0.001 | 3,337 | 1.874 (1.662–2.113) | <0.001 | 1,408 | 1.950 (1.561–2.435) | <0.001 |
| eGDR | 3,345 | 0.540 (0.470–0.620) | <0.001 | 3,329 | 0.541 (0.471–0.622) | <0.001 | 1,410 | 0.565 (0.463–0.689) | <0.001 |
| METS-IR | 3,353 | 1.347 (1.279–1.419) | <0.001 | 3,337 | 1.349 (1.281–1.422) | <0.001 | 1,408 | 1.418 (1.298–1.550) | <0.001 |
| *Model A: adjusted for age, sex, marital status, residence, education, smoking, drinking, BMI, hypertension, diabetes, and lipid-lowering medication. Model B: Model A + self-reported heart disease, stroke, and lung disease. Model C: Model B + physical activity (binary: active vs inactive). RR, risk ratio; CI, confidence interval.* | | | | | | | | | |
