# Supplementary material for: Non-traditional metabolic indices predict incident circadian syndrome in middle-aged and older Chinese adults: a nationwide prospective cohort study and machine learning analysis
Source: Lipids Health Dis. 2026 May 13;25:167. doi: 10.1186/s12944-026-02972-9 (PMC13339493; doi:10.1186/s12944-026-02972-9)
Supplement: Supplementary file 1 — Supplementary Material 1. [file 12944_2026_2972_MOESM1_ESM.zip › Table_S10.docx]

**Table S10. Inverse probability of treatment weighting (IPTW) analysis**

| **Index** | **Index label** | **Method** | **N** | **Events** | **OR** | **Lower CI** | **Upper CI** | **P value** |
| --- | --- | --- | --- | --- | --- | --- | --- | --- |
| AIP | AIP | IPTW logistic | 3,356 | 725 | 1.924 | 1.716 | 2.157 | <0.001 |
| CHG Index | CHG Index | IPTW logistic | 3,004 | 648 | 2.056 | 1.822 | 2.319 | <0.001 |
| eGDR | eGDR | IPTW logistic | 3,345 | 729 | 0.315 | 0.266 | 0.373 | <0.001 |
| *OR, odds ratio; CI, confidence interval; IPTW, inverse probability of treatment weighting.* | | | | | | | | |
