# Supplementary material for: Non-traditional metabolic indices predict incident circadian syndrome in middle-aged and older Chinese adults: a nationwide prospective cohort study and machine learning analysis
Source: Lipids Health Dis. 2026 May 13;25:167. doi: 10.1186/s12944-026-02972-9 (PMC13339493; doi:10.1186/s12944-026-02972-9)
Supplement: Supplementary file 1 — Supplementary Material 1. [file 12944_2026_2972_MOESM1_ESM.zip › Table_S11.docx]

**Table S11. Covariate balance before and after IPTW adjustment**

| **Covariate** | **SMD (unweighted)** | **SMD (IPTW)** | **Index** |
| --- | --- | --- | --- |
| Age | -0.142 | -0.010 | AIP |
| BMI | 0.376 | 0.024 | AIP |
| HTN | 0.100 | 0.016 | AIP |
| DIAB | 0.002 | 0.002 | AIP |
| MED_LIPID | 0.103 | -0.006 | AIP |
| Age | 0.014 | 0.005 | CHG Index |
| BMI | 0.361 | 0.007 | CHG Index |
| HTN | 0.082 | 0.017 | CHG Index |
| DIAB | 0.345 | -0.001 | CHG Index |
| MED_LIPID | 0.116 | <0.001 | CHG Index |
| Age | -0.044 | -0.026 | eGDR |
| BMI | -1.011 | -0.386 | eGDR |
| HTN | -0.607 | -0.455 | eGDR |
| DIAB | -0.167 | -0.082 | eGDR |
| MED_LIPID | -0.200 | -0.115 | eGDR |
| *SMD, standardised mean difference; IPTW, inverse probability of treatment weighting. \|SMD\| < 0.1 indicates adequate balance.* | | | |
