# Supplementary material for: Non-traditional metabolic indices predict incident circadian syndrome in middle-aged and older Chinese adults: a nationwide prospective cohort study and machine learning analysis
Source: Lipids Health Dis. 2026 May 13;25:167. doi: 10.1186/s12944-026-02972-9 (PMC13339493; doi:10.1186/s12944-026-02972-9)
Supplement: Supplementary file 1 — Supplementary Material 1. [file 12944_2026_2972_MOESM1_ESM.zip › Table_S12.docx]

**Table S12. Subgroup analyses with interaction tests for eight metabolic indices**

| **Index** | **Index label** | **RR** | **Lower CI** | **Upper CI** | **P value** | **N** | **Events** | **Subgroup** | **Level** | **P interaction** |
| --- | --- | --- | --- | --- | --- | --- | --- | --- | --- | --- |
| AIP | AIP | 1.320 | 1.228 | 1.420 | <0.001 | 1,973 | 436 | Age group | <60 | 0.443 |
| AIP | AIP | 1.408 | 1.301 | 1.524 | <0.001 | 1,383 | 289 | Age group | >=60 | 0.443 |
| AIP | AIP | 1.329 | 1.236 | 1.430 | <0.001 | 1,793 | 409 | Sex | Female | 0.721 |
| AIP | AIP | 1.384 | 1.283 | 1.494 | <0.001 | 1,563 | 316 | Sex | Male | 0.721 |
| AIP | AIP | 1.321 | 1.248 | 1.398 | <0.001 | 3,154 | 640 | BMI group | <28 | 0.004 |
| AIP | AIP | 1.239 | 1.063 | 1.444 | 0.006 | 202 | 85 | BMI group | >=28 | 0.004 |
| AIP | AIP | 1.375 | 1.288 | 1.468 | <0.001 | 2,779 | 524 | Hypertension | No | 0.397 |
| AIP | AIP | 1.301 | 1.190 | 1.424 | <0.001 | 577 | 201 | Hypertension | Yes | 0.397 |
| AIP | AIP | 1.374 | 1.301 | 1.452 | <0.001 | 3,237 | 677 | Diabetes | No | 0.145 |
| AIP | AIP | 1.114 | 0.933 | 1.331 | 0.234 | 119 | 48 | Diabetes | Yes | 0.145 |
| AIP | AIP | 1.338 | 1.250 | 1.433 | <0.001 | 2,030 | 447 | Smoking | Non-smoker | 0.898 |
| AIP | AIP | 1.370 | 1.258 | 1.492 | <0.001 | 1,326 | 278 | Smoking | Smoker | 0.898 |
| AIP | AIP | 1.363 | 1.235 | 1.504 | <0.001 | 1,042 | 210 | Drinking | Drinker | 0.936 |
| AIP | AIP | 1.351 | 1.268 | 1.440 | <0.001 | 2,314 | 515 | Drinking | Non-drinker | 0.936 |
| CHG Index | CHG Index | 1.308 | 1.207 | 1.416 | <0.001 | 1,775 | 392 | Age group | <60 | 0.195 |
| CHG Index | CHG Index | 1.457 | 1.343 | 1.581 | <0.001 | 1,229 | 256 | Age group | >=60 | 0.195 |
| CHG Index | CHG Index | 1.392 | 1.297 | 1.495 | <0.001 | 1,610 | 371 | Sex | Female | 0.870 |
| CHG Index | CHG Index | 1.346 | 1.227 | 1.477 | <0.001 | 1,394 | 277 | Sex | Male | 0.870 |
| CHG Index | CHG Index | 1.340 | 1.261 | 1.423 | <0.001 | 2,823 | 574 | BMI group | <28 | 0.014 |
| CHG Index | CHG Index | 1.154 | 0.965 | 1.380 | 0.117 | 181 | 74 | BMI group | >=28 | 0.014 |
| CHG Index | CHG Index | 1.416 | 1.327 | 1.511 | <0.001 | 2,490 | 471 | Hypertension | No | 0.041 |
| CHG Index | CHG Index | 1.217 | 1.085 | 1.364 | 0.001 | 514 | 177 | Hypertension | Yes | 0.041 |
| CHG Index | CHG Index | 1.401 | 1.322 | 1.484 | <0.001 | 2,894 | 603 | Diabetes | No | 0.021 |
| CHG Index | CHG Index | 1.176 | 0.958 | 1.443 | 0.121 | 110 | 45 | Diabetes | Yes | 0.021 |
| CHG Index | CHG Index | 1.375 | 1.284 | 1.473 | <0.001 | 1,818 | 405 | Smoking | Non-smoker | 0.759 |
| CHG Index | CHG Index | 1.368 | 1.243 | 1.507 | <0.001 | 1,186 | 243 | Smoking | Smoker | 0.759 |
| CHG Index | CHG Index | 1.434 | 1.292 | 1.591 | <0.001 | 938 | 191 | Drinking | Drinker | 0.346 |
| CHG Index | CHG Index | 1.351 | 1.263 | 1.444 | <0.001 | 2,066 | 457 | Drinking | Non-drinker | 0.346 |
| eGDR | eGDR | 0.500 | 0.425 | 0.588 | <0.001 | 1,964 | 429 | Age group | <60 | 0.176 |
| eGDR | eGDR | 0.608 | 0.476 | 0.776 | <0.001 | 1,381 | 300 | Age group | >=60 | 0.176 |
| eGDR | eGDR | 0.678 | 0.559 | 0.822 | <0.001 | 1,784 | 411 | Sex | Female | 0.138 |
| eGDR | eGDR | 0.395 | 0.315 | 0.496 | <0.001 | 1,561 | 318 | Sex | Male | 0.138 |
| eGDR | eGDR | 0.611 | 0.523 | 0.713 | <0.001 | 3,144 | 645 | BMI group | <28 | <0.001 |
| eGDR | eGDR | 0.821 | 0.553 | 1.220 | 0.329 | 201 | 84 | BMI group | >=28 | <0.001 |
| eGDR | eGDR | 0.683 | 0.629 | 0.741 | <0.001 | 2,770 | 529 | Hypertension | No | <0.001 |
| eGDR | eGDR | 0.912 | 0.791 | 1.051 | 0.204 | 575 | 200 | Hypertension | Yes | <0.001 |
| eGDR | eGDR | 0.522 | 0.450 | 0.605 | <0.001 | 3,228 | 681 | Diabetes | No | 0.424 |
| eGDR | eGDR | 0.660 | 0.421 | 1.034 | 0.070 | 117 | 48 | Diabetes | Yes | 0.424 |
| eGDR | eGDR | 0.640 | 0.529 | 0.775 | <0.001 | 2,005 | 443 | Smoking | Non-smoker | 0.540 |
| eGDR | eGDR | 0.426 | 0.341 | 0.533 | <0.001 | 1,340 | 286 | Smoking | Smoker | 0.540 |
| eGDR | eGDR | 0.468 | 0.371 | 0.590 | <0.001 | 1,027 | 210 | Drinking | Drinker | 0.148 |
| eGDR | eGDR | 0.584 | 0.492 | 0.692 | <0.001 | 2,318 | 519 | Drinking | Non-drinker | 0.148 |
| *RR, risk ratio; CI, confidence interval. P-interaction from multiplicative interaction terms.* | | | | | | | | | | |
