# Supplementary material for: Non-traditional metabolic indices predict incident circadian syndrome in middle-aged and older Chinese adults: a nationwide prospective cohort study and machine learning analysis
Source: Lipids Health Dis. 2026 May 13;25:167. doi: 10.1186/s12944-026-02972-9 (PMC13339493; doi:10.1186/s12944-026-02972-9)
Supplement: Supplementary file 1 — Supplementary Material 1. [file 12944_2026_2972_MOESM1_ESM.zip › Table_S13.docx]

**Table S13. Sensitivity analysis excluding participants with diabetes or hypertension at baseline**

| **Index** | **Index label** | **RR** | **Lower CI** | **Upper CI** | **P value** | **N** | **Events** | **Sensitivity analysis** |
| --- | --- | --- | --- | --- | --- | --- | --- | --- |
| AIP | AIP | 1.356 | 1.285 | 1.430 | <0.001 | 3,356 | 725 | Primary CircS (>=4 components) |
| CHG Index | CHG Index | 1.373 | 1.298 | 1.452 | <0.001 | 3,004 | 648 | Primary CircS (>=4 components) |
| eGDR | eGDR | 0.540 | 0.470 | 0.620 | <0.001 | 3,345 | 729 | Primary CircS (>=4 components) |
| TyG-BMI | TyG-BMI | 1.865 | 1.659 | 2.097 | <0.001 | 3,353 | 725 | Primary CircS (>=4 components) |
| METS-IR | METS-IR | 1.344 | 1.276 | 1.415 | <0.001 | 3,353 | 725 | Primary CircS (>=4 components) |
| CTI | CTI | 1.254 | 1.183 | 1.330 | <0.001 | 3,353 | 725 | Primary CircS (>=4 components) |
| AIP | AIP | 1.374 | 1.301 | 1.452 | <0.001 | 3,237 | 677 | Exclude participants with diabetes |
| CHG Index | CHG Index | 1.401 | 1.322 | 1.484 | <0.001 | 2,894 | 603 | Exclude participants with diabetes |
| eGDR | eGDR | 0.522 | 0.450 | 0.605 | <0.001 | 3,228 | 681 | Exclude participants with diabetes |
| TyG-BMI | TyG-BMI | 1.939 | 1.716 | 2.190 | <0.001 | 3,234 | 677 | Exclude participants with diabetes |
| METS-IR | METS-IR | 1.352 | 1.281 | 1.426 | <0.001 | 3,234 | 677 | Exclude participants with diabetes |
| CTI | CTI | 1.269 | 1.195 | 1.348 | <0.001 | 3,234 | 677 | Exclude participants with diabetes |
| AIP | AIP | 1.375 | 1.288 | 1.468 | <0.001 | 2,779 | 524 | Exclude participants with hypertension |
| CHG Index | CHG Index | 1.416 | 1.327 | 1.511 | <0.001 | 2,490 | 471 | Exclude participants with hypertension |
| eGDR | eGDR | 0.683 | 0.629 | 0.741 | <0.001 | 2,770 | 529 | Exclude participants with hypertension |
| TyG-BMI | TyG-BMI | 1.978 | 1.719 | 2.276 | <0.001 | 2,776 | 524 | Exclude participants with hypertension |
| METS-IR | METS-IR | 1.359 | 1.275 | 1.449 | <0.001 | 2,776 | 524 | Exclude participants with hypertension |
| CTI | CTI | 1.283 | 1.197 | 1.374 | <0.001 | 2,776 | 524 | Exclude participants with hypertension |
| AIP | AIP | 1.363 | 1.291 | 1.439 | <0.001 | 3,288 | 700 | Exclude diabetes medication users |
| CHG Index | CHG Index | 1.395 | 1.317 | 1.477 | <0.001 | 2,940 | 623 | Exclude diabetes medication users |
| eGDR | eGDR | 0.538 | 0.466 | 0.620 | <0.001 | 3,277 | 704 | Exclude diabetes medication users |
| TyG-BMI | TyG-BMI | 1.887 | 1.676 | 2.125 | <0.001 | 3,285 | 700 | Exclude diabetes medication users |
| METS-IR | METS-IR | 1.350 | 1.280 | 1.423 | <0.001 | 3,285 | 700 | Exclude diabetes medication users |
| CTI | CTI | 1.258 | 1.185 | 1.335 | <0.001 | 3,285 | 700 | Exclude diabetes medication users |
| *RR, risk ratio; CI, confidence interval. Modified Poisson regression with robust variance.* | | | | | | | | |
