# Supplementary material for: Non-traditional metabolic indices predict incident circadian syndrome in middle-aged and older Chinese adults: a nationwide prospective cohort study and machine learning analysis
Source: Lipids Health Dis. 2026 May 13;25:167. doi: 10.1186/s12944-026-02972-9 (PMC13339493; doi:10.1186/s12944-026-02972-9)
Supplement: Supplementary file 1 — Supplementary Material 1. [file 12944_2026_2972_MOESM1_ESM.zip › Table_S14.docx]

**Table S14. Performance comparison of ten machine learning algorithms**

| **Model** | **N (test)** | **Events (test)** | **AUC** | **AUC lower CI** | **AUC upper CI** | **Brier score** | **Threshold** | **Sensitivity** | **Specificity** | **PPV** | **NPV** | **F1 score** | **Accuracy** |
| --- | --- | --- | --- | --- | --- | --- | --- | --- | --- | --- | --- | --- | --- |
| Logistic regression | 880 | 189 | 0.746 | 0.710 | 0.784 | 0.151 | 0.176 | 0.783 | 0.640 | 0.373 | 0.915 | 0.505 | 0.670 |
| Naive Bayes | 880 | 189 | 0.737 | 0.697 | 0.777 | 0.214 | 0.052 | 0.767 | 0.653 | 0.377 | 0.911 | 0.505 | 0.677 |
| LASSO logistic | 880 | 189 | 0.737 | 0.698 | 0.777 | 0.160 | 0.229 | 0.561 | 0.799 | 0.433 | 0.869 | 0.488 | 0.748 |
| Random forest | 880 | 189 | 0.731 | 0.688 | 0.767 | 0.149 | 0.166 | 0.794 | 0.575 | 0.338 | 0.911 | 0.474 | 0.622 |
| XGBoost | 880 | 189 | 0.721 | 0.681 | 0.759 | 0.154 | 0.181 | 0.640 | 0.699 | 0.368 | 0.877 | 0.467 | 0.686 |
| k-Nearest neighbors | 880 | 189 | 0.717 | 0.676 | 0.755 | 0.154 | 0.186 | 0.788 | 0.553 | 0.325 | 0.905 | 0.461 | 0.603 |
| Multilayer perceptron | 880 | 189 | 0.714 | 0.665 | 0.758 | 0.154 | 0.323 | 0.587 | 0.787 | 0.430 | 0.875 | 0.497 | 0.744 |
| LightGBM | 880 | 189 | 0.713 | 0.673 | 0.754 | 0.165 | 0.096 | 0.688 | 0.654 | 0.352 | 0.885 | 0.466 | 0.661 |
| Decision tree | 880 | 189 | 0.708 | 0.664 | 0.748 | 0.154 | 0.204 | 0.646 | 0.718 | 0.385 | 0.881 | 0.482 | 0.702 |
| SVM (RBF) | 880 | 189 | 0.659 | 0.611 | 0.706 | 0.158 | 0.192 | 0.571 | 0.741 | 0.376 | 0.863 | 0.454 | 0.705 |
| *AUC, area under the curve; CI, confidence interval; F1, F1 score; PPV, positive predictive value; NPV, negative predictive value.* | | | | | | | | | | | | | |
