# Supplementary material for: Non-traditional metabolic indices predict incident circadian syndrome in middle-aged and older Chinese adults: a nationwide prospective cohort study and machine learning analysis
Source: Lipids Health Dis. 2026 May 13;25:167. doi: 10.1186/s12944-026-02972-9 (PMC13339493; doi:10.1186/s12944-026-02972-9)
Supplement: Supplementary file 1 — Supplementary Material 1. [file 12944_2026_2972_MOESM1_ESM.zip › Table_S15.docx]

**Table S15. LASSO logistic regression coefficients at lambda.min**

| **variable** | **coefficient** | **selected** |
| --- | --- | --- |
| METS_IR_w1 | 1.02750354080870875961 | true |
| AIP_w1 | 0.60818607226742904537 | true |
| CHG_w1 | 0.55632168890968380914 | true |
| HTN | -0.34708937102923459461 | true |
| eGDR_w1 | -0.29100895046842184533 | true |
| Marital_statusWidowed/Never married | 0.24558256861944641192 | true |
| SexFemale | 0.12016593869272479411 | true |
| hsCRP_HDL_w1 | 0.11679107751587612662 | true |
| BMI | 0.08356831246550107395 | true |
| SmokingSmoker | 0.07031029254711247678 | true |
| DrinkingNon-drinker | -0.03172265819136090709 | true |
| Age | 0.00197029282039683012 | true |
| SexMale | -0.00000000000001112811 | true |
| RCII_w1 | 0.00000000000000000000 | false |
| CTI_w1 | 0.00000000000000000000 | false |
| TyG_BMI_w1 | 0.00000000000000000000 | false |
| Marital_statusSeparated/Divorced | 0.00000000000000000000 | false |
| ResidenceUrban | 0.00000000000000000000 | false |
| EducationMiddle school or above | 0.00000000000000000000 | false |
| DIAB | 0.00000000000000000000 | false |
| MED_LIPID | 0.00000000000000000000 | false |
| *LASSO, least absolute shrinkage and selection operator.* | | |
