# Supplementary material for: Non-traditional metabolic indices predict incident circadian syndrome in middle-aged and older Chinese adults: a nationwide prospective cohort study and machine learning analysis
Source: Lipids Health Dis. 2026 May 13;25:167. doi: 10.1186/s12944-026-02972-9 (PMC13339493; doi:10.1186/s12944-026-02972-9)
Supplement: Supplementary file 1 — Supplementary Material 1. [file 12944_2026_2972_MOESM1_ESM.zip › Table_S16.docx]

**Table S16. Optimal cutoff values for each metabolic index determined by Youden index**

| **Index** | **N** | **Events** | **Optimal cutoff** | **Sensitivity (%)** | **Specificity (%)** | **PPV (%)** | **NPV (%)** | **Youden index** | **AUC (95% CI)** |
| --- | --- | --- | --- | --- | --- | --- | --- | --- | --- |
| TyG-BMI | 3,645 | 787 | 184.306 | 76.4 | 55.2 | 31.9 | 89.4 | 0.315 | 0.710 (0.690–0.729) |
| eGDR | 3,645 | 798 | 10.818 | 70.8 | 60.6 | 33.5 | 88.1 | 0.314 | 0.702 (0.682–0.722) |
| METS-IR | 3,637 | 784 | 2.230 | 61.4 | 64.9 | 32.4 | 85.9 | 0.262 | 0.675 (0.654–0.695) |
| CHG Index | 3,791 | 885 | 5.136 | 70.4 | 55.7 | 32.6 | 86.1 | 0.261 | 0.669 (0.648–0.689) |
| AIP | 4,216 | 986 | 0.207 | 70.4 | 54.6 | 32.1 | 85.8 | 0.250 | 0.659 (0.640–0.678) |
| CTI | 4,211 | 985 | 8.546 | 57.5 | 63.9 | 32.7 | 83.1 | 0.214 | 0.634 (0.614–0.654) |
| RCII | 4,214 | 986 | 1.559 | 58.4 | 59.3 | 30.5 | 82.4 | 0.177 | 0.611 (0.591–0.631) |
| hs-CRP/HDL-C | 4,218 | 986 | 0.017 | 60.2 | 55.9 | 29.4 | 82.2 | 0.162 | 0.600 (0.580–0.620) |
| *AUC, area under the curve; CI, confidence interval; NPV, negative predictive value; PPV, positive predictive value; Se, sensitivity; Sp, specificity.* | | | | | | | | | |
