# Supplementary material for: Non-traditional metabolic indices predict incident circadian syndrome in middle-aged and older Chinese adults: a nationwide prospective cohort study and machine learning analysis
Source: Lipids Health Dis. 2026 May 13;25:167. doi: 10.1186/s12944-026-02972-9 (PMC13339493; doi:10.1186/s12944-026-02972-9)
Supplement: Supplementary file 1 — Supplementary Material 1. [file 12944_2026_2972_MOESM1_ESM.zip › Table_S17.docx]

**Table S17. SHAP feature importance from the XGBoost model for incident CircS prediction**

| **Rank** | **Feature** | **Category** | **Mean \|SHAP\|** | **Direction** |
| --- | --- | --- | --- | --- |
| 1 | eGDR | Metabolic index | 0.487 | Lower values → higher risk |
| 2 | TyG-BMI | Metabolic index | 0.359 | Higher values → higher risk |
| 3 | CHG Index | Metabolic index | 0.252 | Higher values → higher risk |
| 4 | METS-IR | Metabolic index | 0.219 | Higher values → higher risk |
| 5 | AIP | Metabolic index | 0.174 | Higher values → higher risk |
| 6 | RCII | Metabolic index | 0.154 | Higher values → higher risk |
| 7 | BMI (kg/m²) | Clinical | 0.142 | Higher values → higher risk |
| 8 | hs-CRP/HDL-C | Metabolic index | 0.110 | Higher values → higher risk |
| 9 | Age (years) | Demographic | 0.110 | Older age → higher risk |
| 10 | CTI | Metabolic index | 0.099 | Higher values → higher risk |
| 11 | Sex (female) | Demographic | 0.063 | Female → higher risk |
| 12 | Education (≥ middle school) | Demographic | 0.043 | Higher education → lower risk |
| 13 | Alcohol consumption (non-drinker) | Lifestyle | 0.027 | Non-drinking → higher risk |
| 14 | Smoking status (smoker) | Lifestyle | 0.021 | Smoking → higher risk |
| 15 | Marital status (widowed/never married) | Demographic | 0.021 | Unmarried → higher risk |
| 16 | Diabetes | Clinical | 0.014 | Diabetes → higher risk |
| 17 | Sex (male) | Demographic | 0.009 | Male → higher risk |
| 18 | Residence (urban) | Demographic | 0.008 | Urban → lower risk |
| 19 | Hypertension | Clinical | 0.007 | Hypertension → higher risk |
| 20 | Lipid-lowering medication | Clinical | 0.002 | Medication use → lower risk |
| 21 | Marital status (separated/divorced) | Demographic | 0.000 | Separated → higher risk |
| *Features are ranked by mean absolute SHAP value in descending order. Mean \|SHAP\| represents the average magnitude of each feature's contribution to the model's prediction across the test set. Direction of effect was determined from SHAP dependence and beeswarm plots (Fig. S6). Abbreviations: AIP, atherogenic index of plasma; BMI, body mass index; CHG, cholesterol–HDL–glucose index; CircS, circadian syndrome; CTI, C-reactive protein triglyceride glucose index; eGDR, estimated glucose disposal rate; hs-CRP, high-sensitivity C-reactive protein; HDL-C, high-density lipoprotein cholesterol; METS-IR, metabolic score for insulin resistance; RCII, remnant cholesterol inflammation index; SHAP, Shapley additive explanations; TyG-BMI, triglyceride–glucose–body mass index; XGBoost, extreme gradient boosting.* | | | | |
