# Supplementary figures and images for: Non-traditional metabolic indices predict incident circadian syndrome in middle-aged and older Chinese adults: a nationwide prospective cohort study and machine learning analysis
Source: Lipids Health Dis. 2026 May 13;25:167. doi: 10.1186/s12944-026-02972-9 (PMC13339493; doi:10.1186/s12944-026-02972-9)

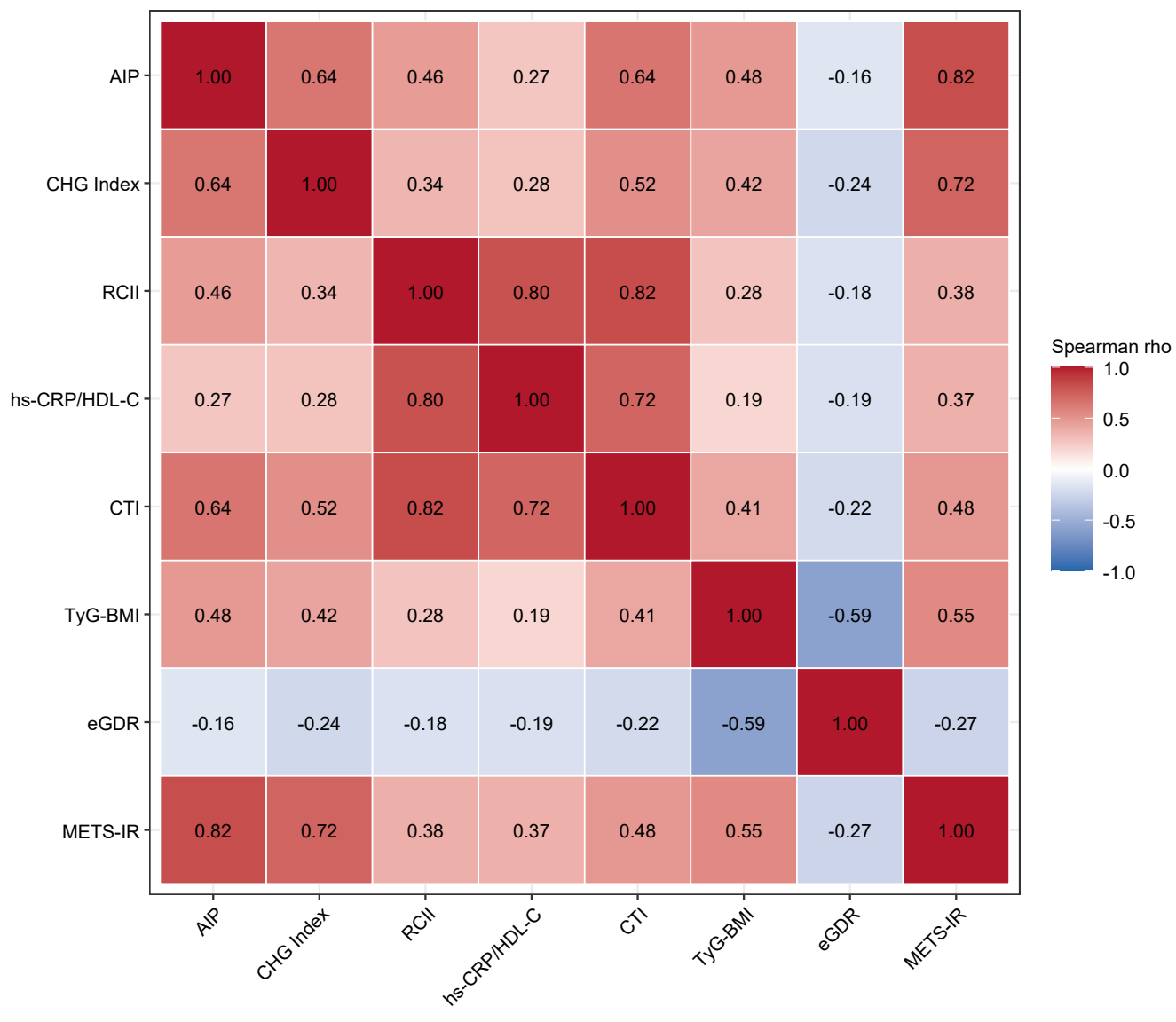

Supplement: Supplementary file 1 — Supplementary Material 1. [file 12944_2026_2972_MOESM1_ESM.zip › Figure_S01.pdf]

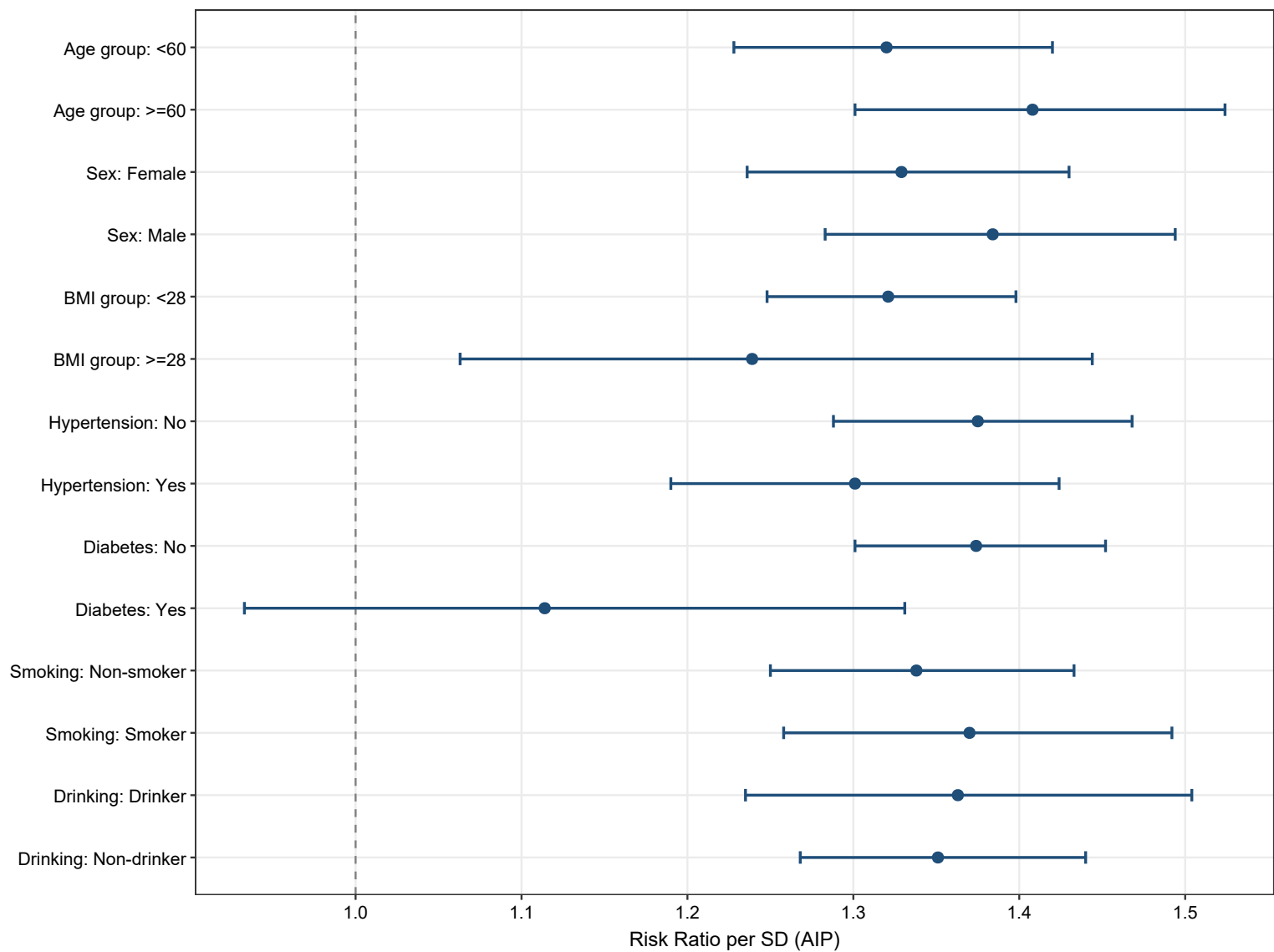

Supplement: Supplementary file 1 — Supplementary Material 1. [file 12944_2026_2972_MOESM1_ESM.zip › Figure_S02.pdf]

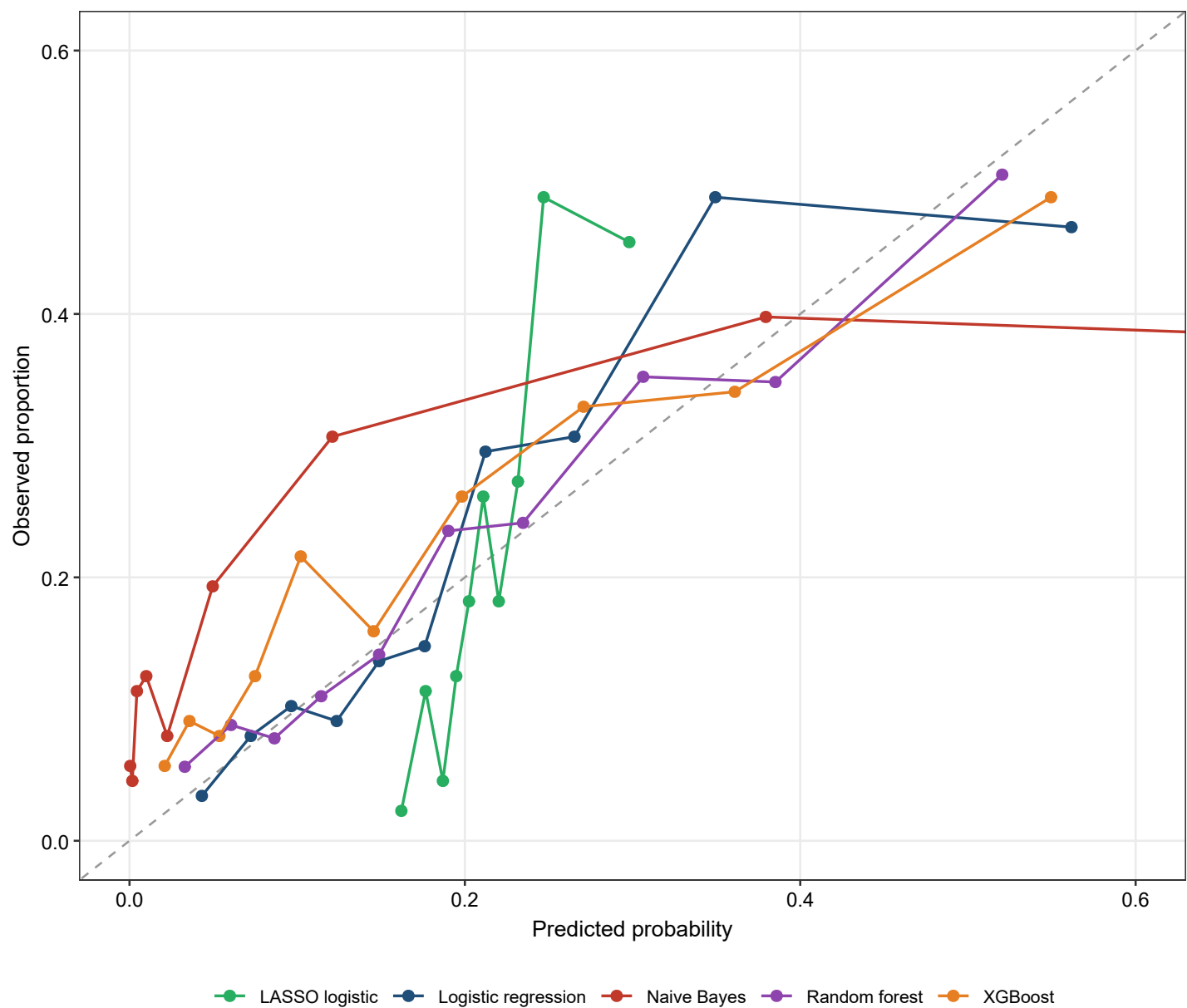

Supplement: Supplementary file 1 — Supplementary Material 1. [file 12944_2026_2972_MOESM1_ESM.zip › Figure_S03.pdf]

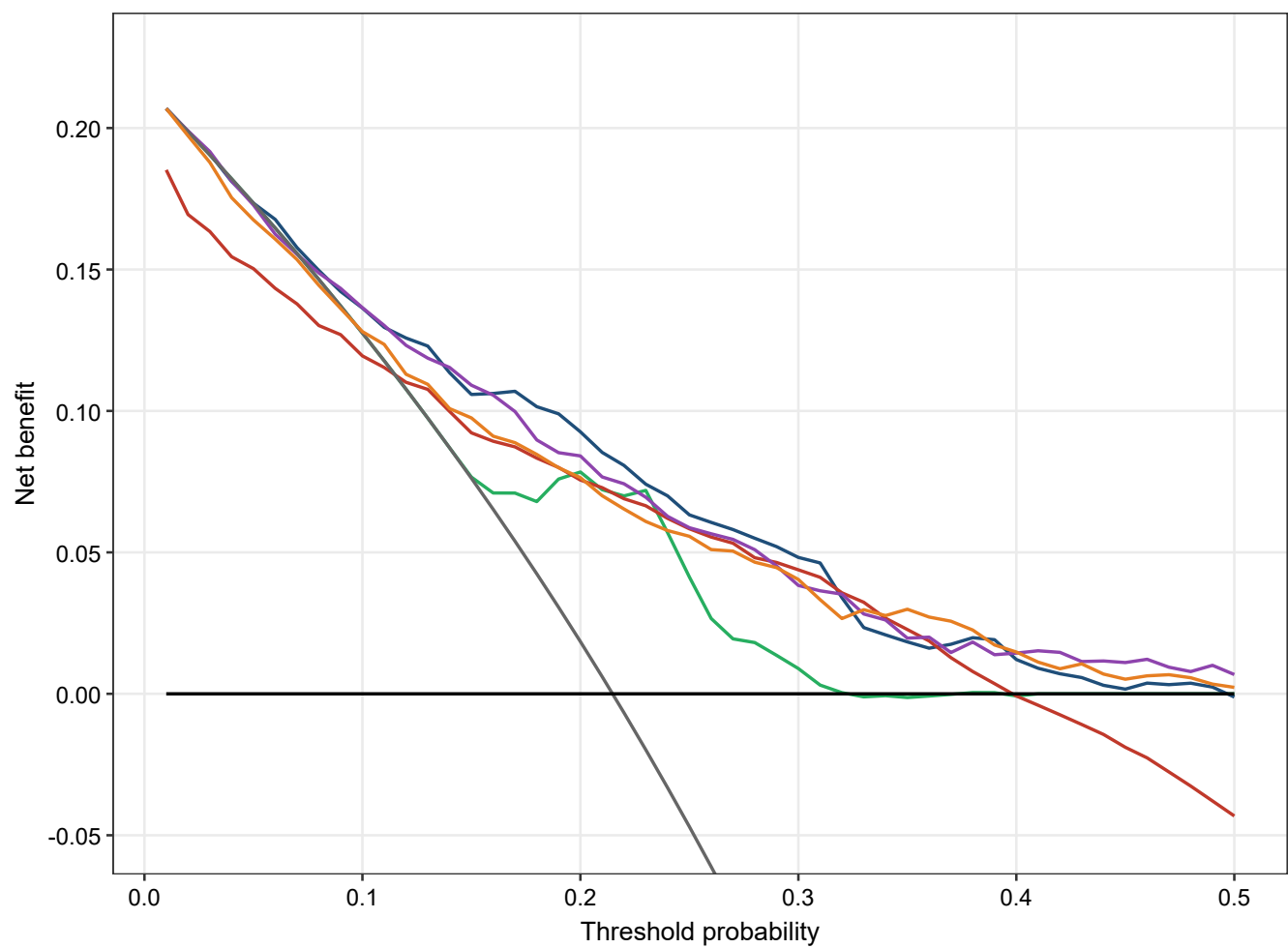

— LASSO logistic — Naive Bayes — Treat All — XGBoost  
— Logistic regression — Random forest — Treat None

Supplement: Supplementary file 1 — Supplementary Material 1. [file 12944_2026_2972_MOESM1_ESM.zip › Figure_S04.pdf]

### A. LASSO coefficient paths

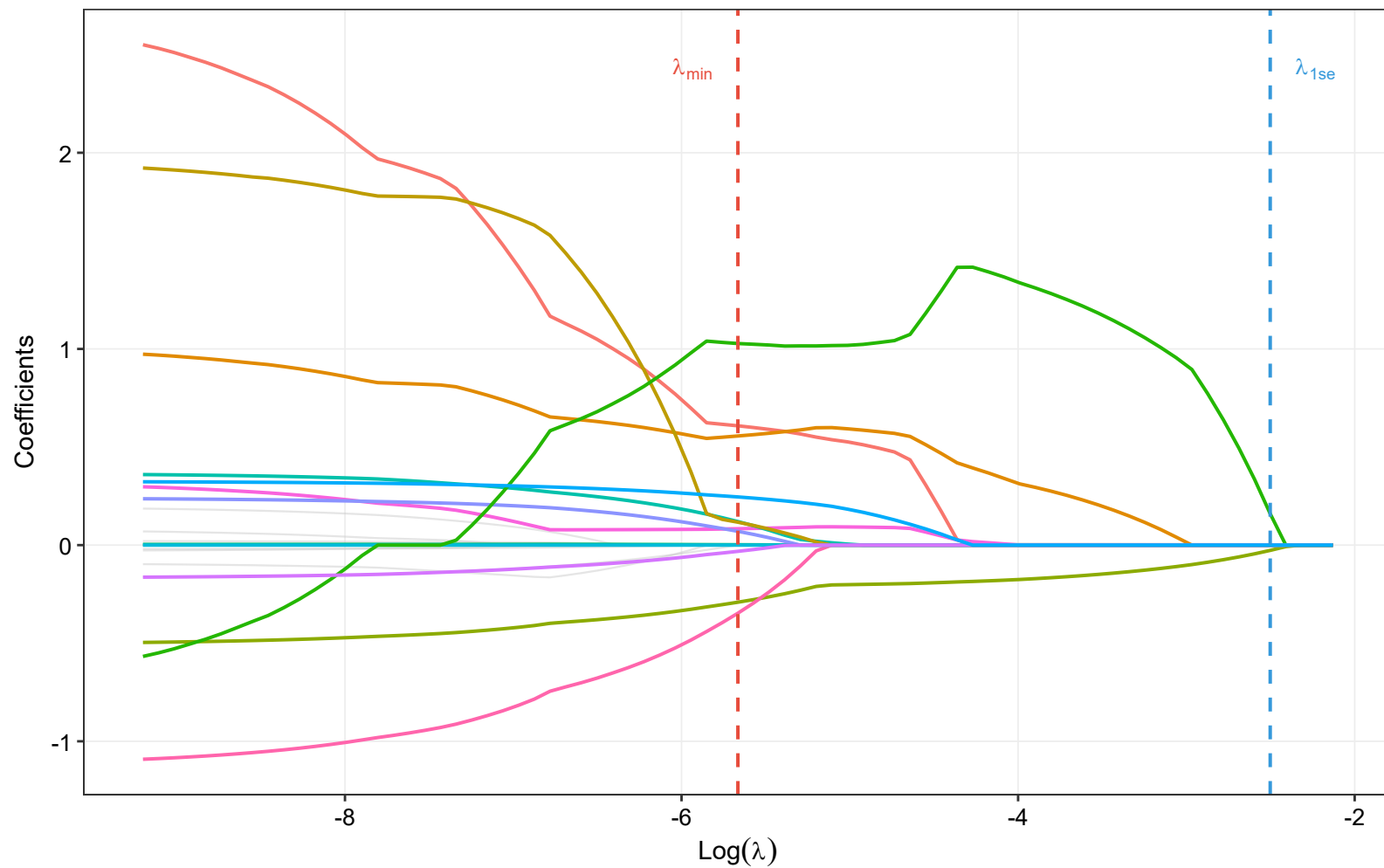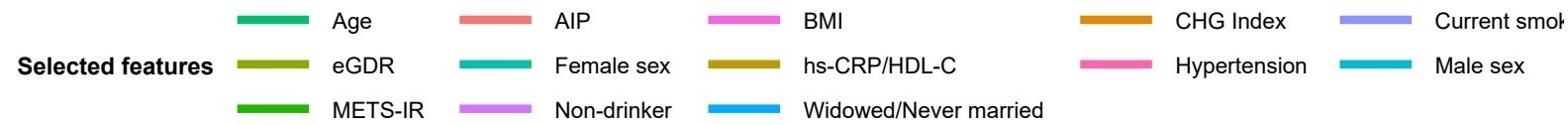

### B. Cross-validation for LASSO

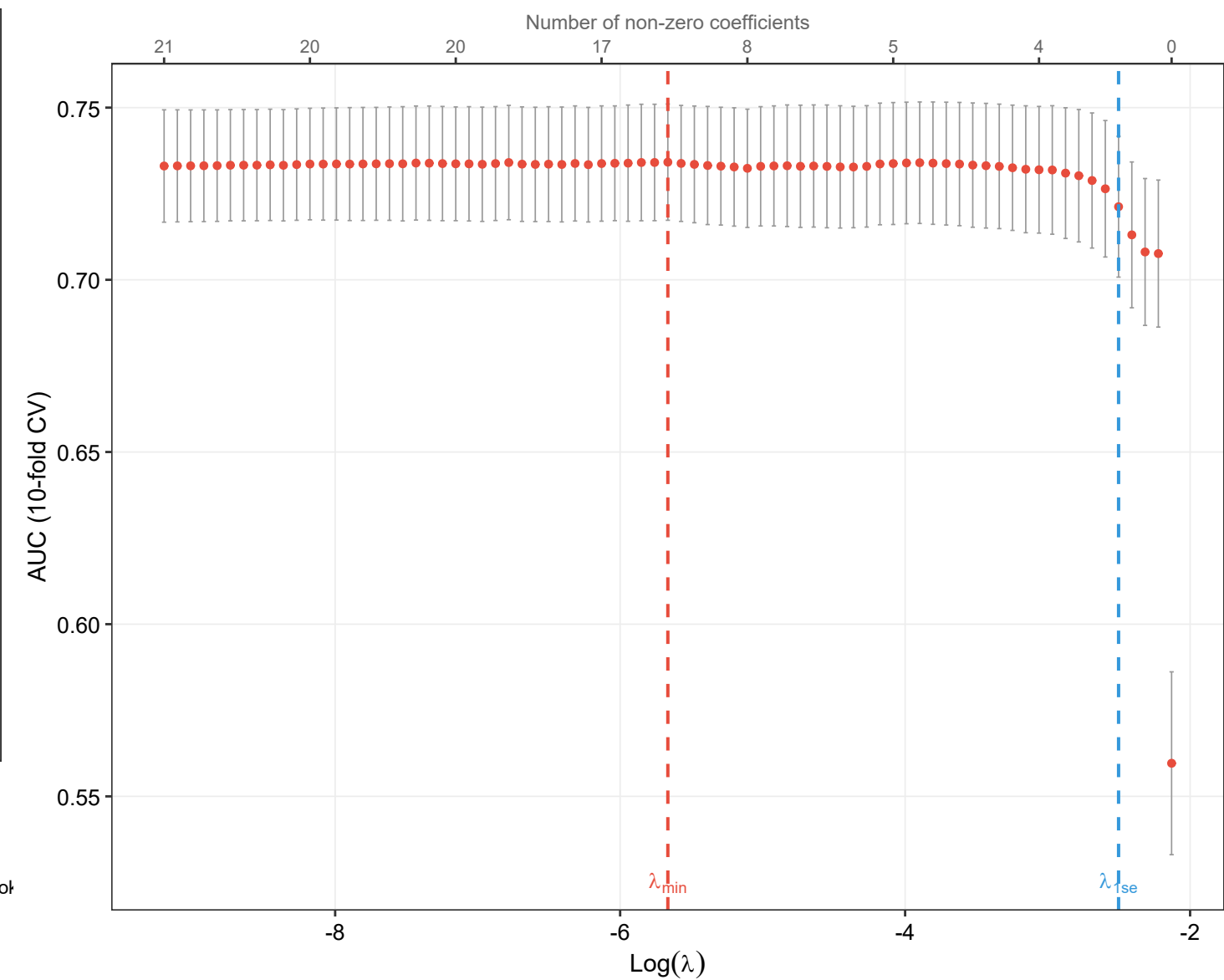

Supplement: Supplementary file 1 — Supplementary Material 1. [file 12944_2026_2972_MOESM1_ESM.zip › Figure_S05.pdf]

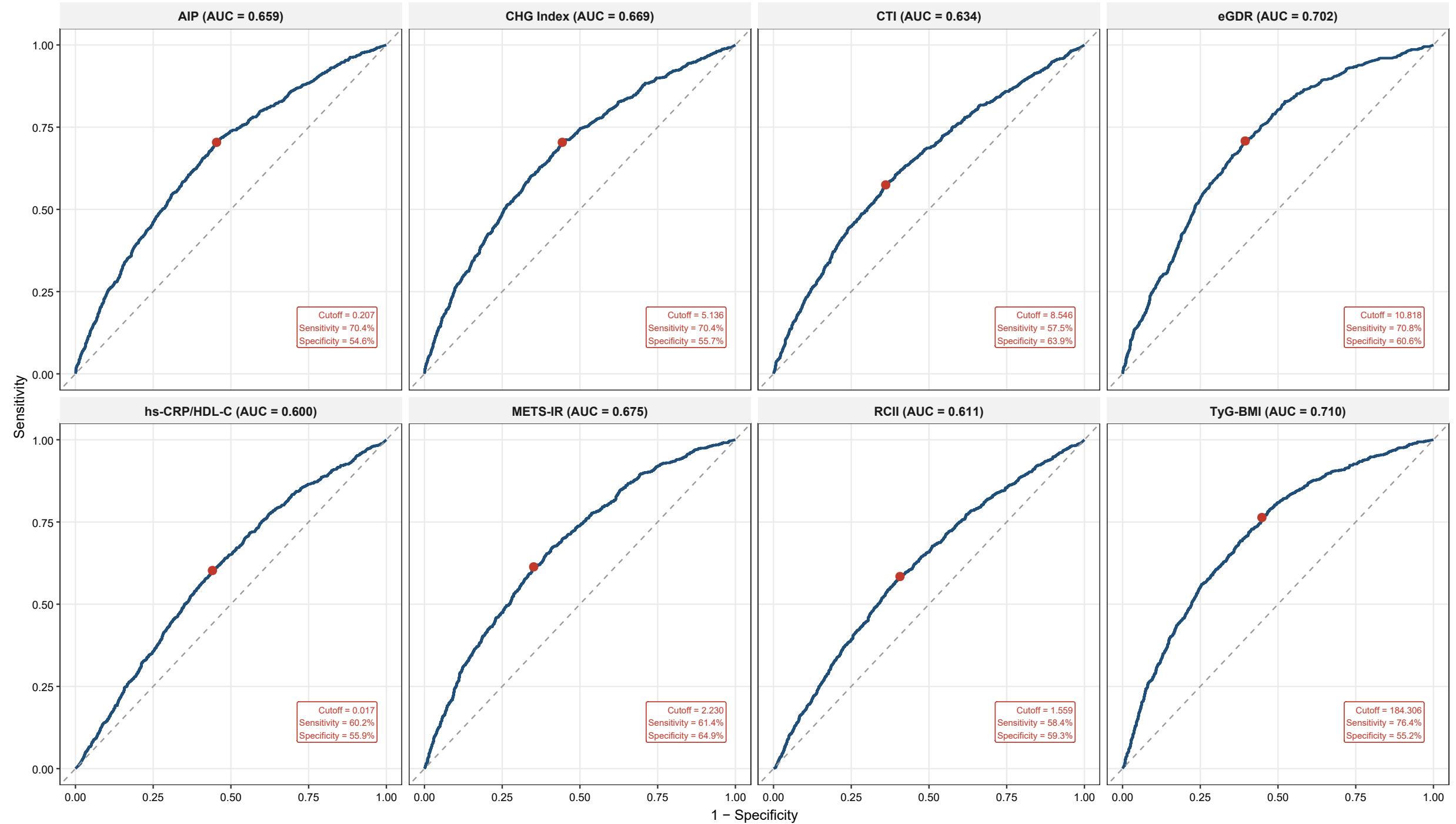

Supplement: Supplementary file 1 — Supplementary Material 1. [file 12944_2026_2972_MOESM1_ESM.zip › Figure_S06.pdf]

**SHAP Summary Plot (XGBoost)**

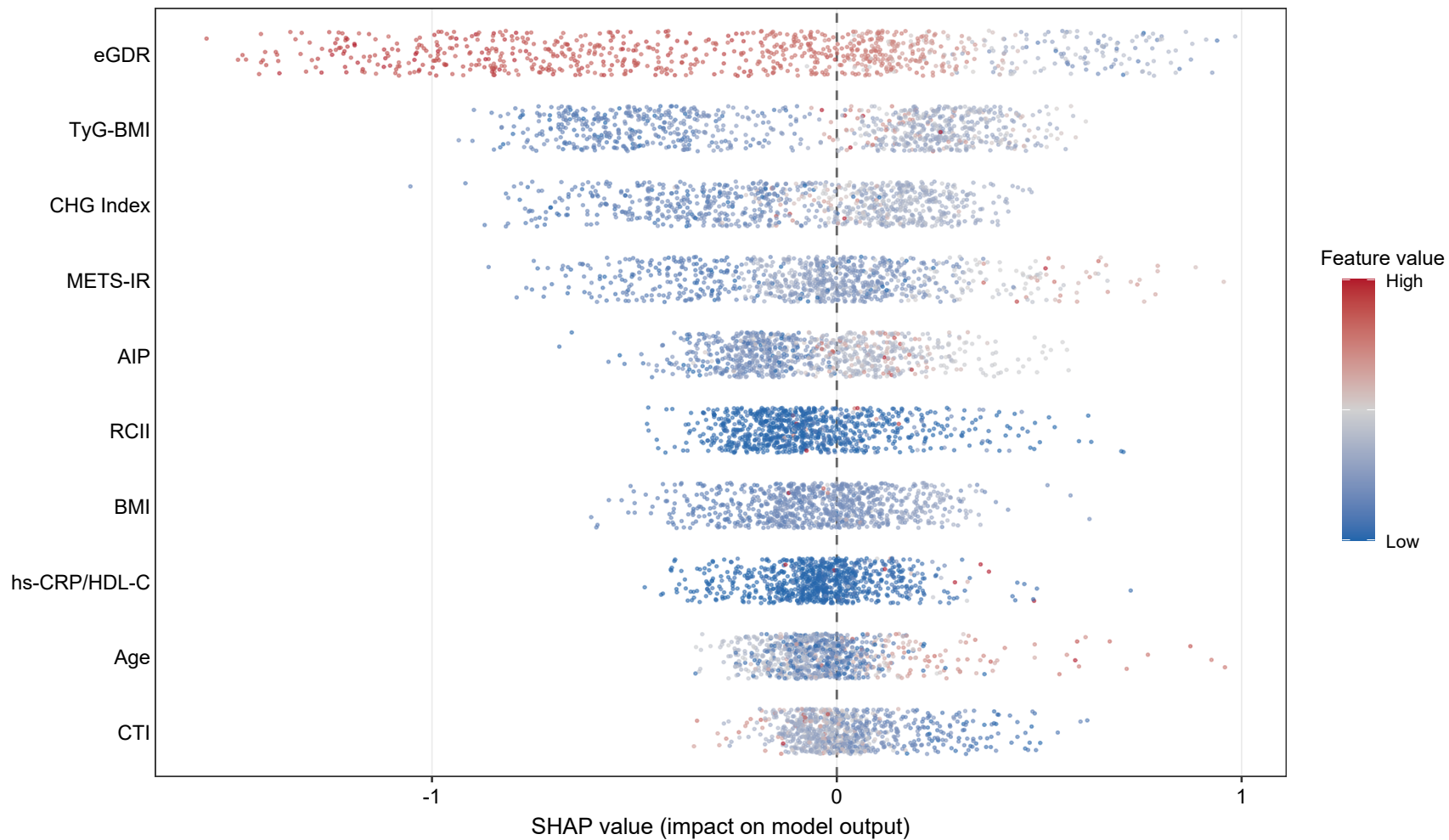

Supplement: Supplementary file 1 — Supplementary Material 1. [file 12944_2026_2972_MOESM1_ESM.zip › Figure_S07.pdf]
